# 4′-Methylflavanone Glycosides Obtained Using Biotransformation in the Entomopathogenic Filamentous Fungi Cultures as Potential Anticarcinogenic, Antimicrobial, and Hepatoprotective Agents

**DOI:** 10.3390/ijms23105373

**Published:** 2022-05-11

**Authors:** Agnieszka Krawczyk-Łebek, Monika Dymarska, Tomasz Janeczko, Edyta Kostrzewa-Susłow

**Affiliations:** Department of Food Chemistry and Biocatalysis, Faculty of Biotechnology and Food Science, Wrocław University of Environmental and Life Sciences, 50-375 Wrocław, Poland; monika.dymarska@upwr.edu.pl (M.D.); tomasz.janeczko@upwr.edu.pl (T.J.)

**Keywords:** biotransformations, 4′-methylflavanone, glycosylation, *O*-methylglucosides, *Beauveria bassiana*, *Isaria fumosorosea*, *Isaria farinosa*

## Abstract

Flavonoid compounds exhibit numerous biological activities and significantly impact human health. The presence of methyl or glucosyl moieties attached to the flavonoid core remarkably modifies their physicochemical properties and improves intestinal absorption. Combined chemical and biotechnological methods can be applied to obtain such derivatives. In the presented study, 4′-methylflavanone was synthesized and biotransformed in the cultures of three strains of entomopathogenic filamentous fungi, i.e., *Isaria fumosorosea* KCH J2, *Beauveria bassiana* KCH J1.5, and *Isaria farinosa* KCH J2.1. The microbial transformation products in the culture of *I. fumosorosea* KCH J2, flavanone 4′-methylene-*O*-*β*-D-(4″-*O*-methyl)-glucopyranoside, 2-phenyl-(4′-hydroxymethyl)-4-hydroxychromane, and flavanone 4′-carboxylic acid were obtained. Biotransformation of 4′-methylflavanone in the culture of *B. bassiana* KCH J1.5 resulted in the formation of one main product, i.e., flavanone 4′-methylene-*O*-*β*-D-(4″-*O*-methyl)-glucopyranoside. In the case of *I. farinosa* KCH J2.6 as a biocatalyst, three products, i.e., flavanone 4′-methylene-*O*-*β*-D-(4″-*O*-methyl)-glucopyranoside, flavanone 4′-carboxylic acid, and 4′-hydroxymethylflavanone 4*-O*-*β*-D-(4″-*O*-methyl)-glucopyranoside were obtained. The Swiss-ADME online simulations confirmed the increase in water solubility of 4′-methylflavanone glycosides and analyses performed using the Way2Drug Pass Online prediction tool indicated that flavanone 4′-methylene-*O*-*β*-D-(4″-*O*-methyl)-glucopyranoside and 4′-hydroxymethylflavanone 4*-O*-*β*-D-(4″-*O*-methyl)-glucopyranoside, which had not been previously reported in the literature, are promising anticarcinogenic, antimicrobial, and hepatoprotective agents.

## 1. Introduction

The beneficial effect of flavonoid compounds on human health makes them the subject of special research interest. Their basic structure consists of C-6-C-3-C-6 rings with two aromatic A and B rings connected by three carbon-ring C. Among a great variety of flavonoids, we can distinguish such subclasses as flavanones, flavones, flavanols, and isoflavones [1,2]. The substitution of the core structure of flavonoids with various functional groups affects their biological activity and physicochemical properties. These secondary polyphenolic metabolites of plants are usually present in nature as glycosides with one or more sugar moieties attached to a flavonoid aglycone via *O*- or *C*-glycosidic bonds. In this form, they are more stable and water-soluble than their aglycones [3,4,5]. However, the bioavailability of flavonoids is affected by many factors, including molecular weight, the structure of the flavonoid core, the position of the sugar attachment and the number of sugar groups, and the interaction with colonic microflora [4,5,6,7].

Flavonoids are known as valuable diet components in preventing many diseases such as cardiovascular, arteriosclerosis, and diabetes. They have also been described as potential therapeutic agents for microbial infections, inflammation, and cancer [8,9,10], and in addition, hepatoprotective agents [11,12,13]. Their anticancer activity relies on inter alia arresting of the cell cycle, inducing cell apoptosis, and suppressing cancer cell proliferation. Flavonoids are involved in regulating the function of non-coding microRNAs, which leads to modulation of inflammation, angiogenesis, metastasis, and epithelial-to-mesenchymal transition (EMT) [9,14]. These compounds also show synergistic enhancement of anti-glioblastoma effects of conventional chemotherapeutic drugs [15]. In spite of their effective properties in counteracting tumor growth, the detailed mechanisms of action, the potential for adverse interactions, and their safety require further research.

**Scheme 1:**
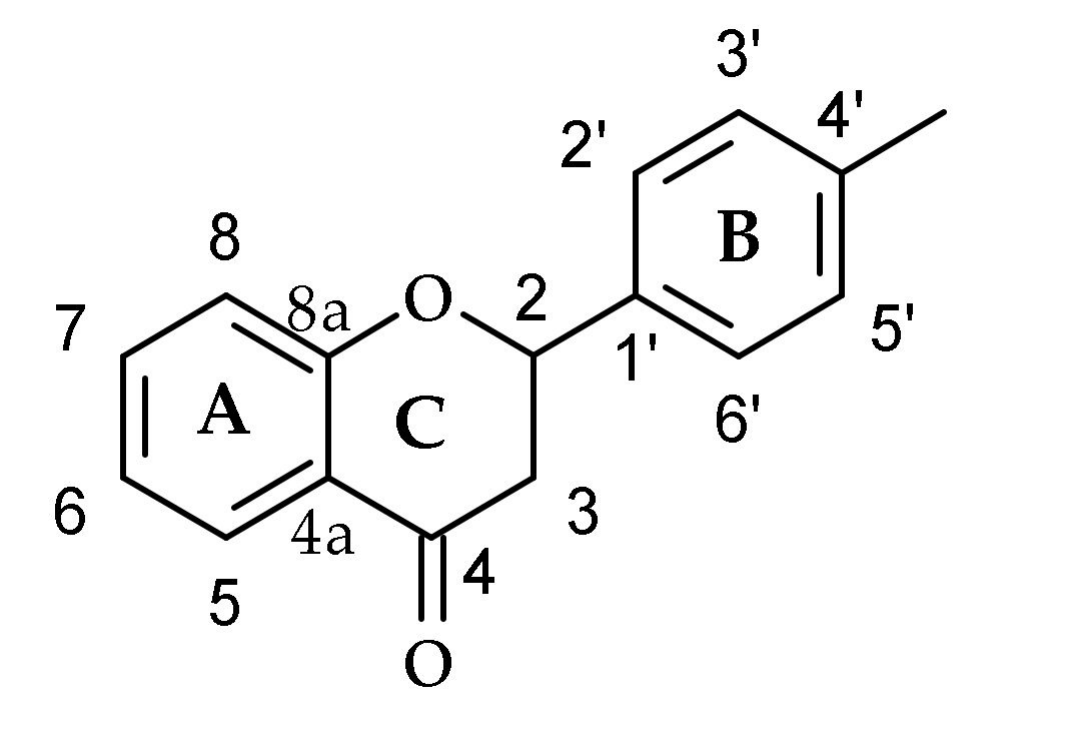
The chemical structure of flavonoid compound, 4′-methylflavanone (**4**).

The methyl and hydroxyl moieties attached to the flavonoid core also modulate their properties. Methylation of flavonoids via their free hydroxyl groups or C atom positively impacts their metabolic stability and membrane transport. Consequently, methylated flavonoids are better absorbed and have increased oral bioavailability [16,17,18]. Methylated flavonoids have been tested as potential inhibitors of aldose reductase, an enzyme whose increased activity is associated with hyperglycemia and diabetes. Most of the tested derivatives, including 4′-methylflavanone (Scheme 1), exhibited moderate activity with IC_50_ in the range of 6.09–7.89 μM [19]. Methylated flavanones have also been evaluated as potential inhibitors of monoamine oxidase isoforms (MAO-A and -B), which play a vital role in the metabolism of monoamine neurotransmitters [20].

The pharmacological application of flavonoid compounds is limited by their relatively low concentration in plants and the low yield of chemical synthesis of flavonoid glycosides [3,21,22]. However, combined chemical and biotechnological methods can overcome these obstacles. The presented studies are a continuation of our previous work on glycosylation of C-methylated flavonoids using biotransformations in the cultures of entomopathogenic filamentous fungi [23,24,25]. The main aim of this work was to obtain glycosylated derivatives of 4′-methylflavanone. For this purpose, microbial transformations of 4′-methylflavanone were performed using three strains of entomopathogenic filamentous fungi, i.e., *Isaria fumosorosea* KCH J2, *Beauveria bassiana* KCH J1.5, and *Isaria farinosa* KCH J2.6. According to our best knowledge, obtained glycosylated derivatives, i.e., flavanone 4′-methylene-*O*-*β*-D-(4″-*O*-methyl)-glucopyranoside, 4′-hydroxymethylflavanone 4*-O*-*β*-D-(4″-*O*-methyl)-glucopyranoside, and 2-phenyl-(4′-hydroxymethyl)-4-hydroxychromane have not been described in the literature. Using computer-aided simulations, the structure–activity, druglike nature, and medicinal chemistry friendliness showed that obtained biotransformation products should be considered potentially bioactive anticancer, antimicrobial, and hepatoprotective agents.

## 2. Results and Discussion

The biotransformation substrate—4′-methylflavanone (**4**)—was obtained by our team in a two-step synthesis [26]. The products of biotransformation were isolated and purified using preparative thin-layer chromatography (PTLC). The chemical structures of all biotransformation products were determined based on nuclear magnetic resonance spectroscopy (NMR spectroscopy) and confirmed by liquid chromatography–mass spectrometry (LC–MS).

### 2.1. Biotransformation of 4′-Methylflavanone (***4***) in the Culture of I. fumosorosea KCH J2

**Scheme 2:**
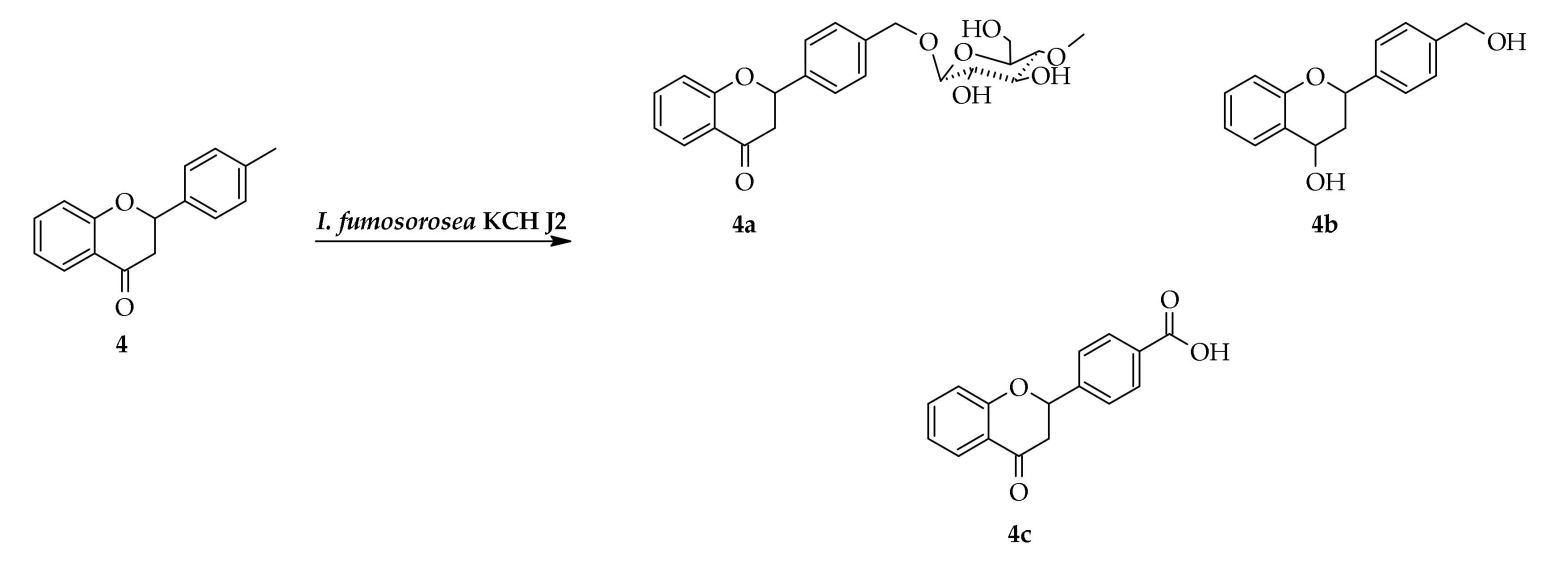
Microbial transformation of 4′-methylflavanone (**4**) in *I. fumosorosea* KCH J2 culture.

As a result of biotransformation of 4′-methylflavanone in the culture of *I. fumosorosea* KCH J2, three products, i.e., flavanone 4′-methylene-*O*-*β*-D-(4″-*O*-methyl)-glucopyranoside (**4a**) with 13.7% yield (12.4 mg), 2-phenyl-(4′-hydroxymethyl)-4-hydroxychromane (**4b**) with 5.6% yield (3.0 mg), and flavanone 4′-carboxylic acid (**4c**) with 6.0% yield (3.4 mg) were obtained (Scheme 2).

**Scheme 3:**
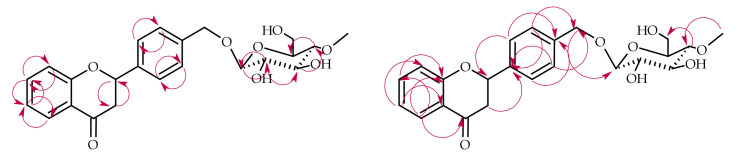
Key COSY (**on the left**) and HMBC (**on the right**) correlations for the structure elucidation of product **4a**.

In the case of product **4a**, 4″-*O*-methyl-glycosylation, which occurred at C-4′-CH_3_ was observed (Scheme 3). The characteristic carbon signals visible in the region from about δ = 80 ppm to about δ = 62 ppm in the carbon-13 nuclear magnetic resonance (^13^C-NMR) spectrum (Supplementary Materials: Figure S28) and protons H2″–H6″ signals ranging from about δ = 3.8 ppm to about δ = 3.1 ppm in the proton nuclear magnetic resonance (^1^H-NMR) spectrum (Supplementary Materials: Figure S25) evidenced the presence of a glycosidic moiety. Additionally, the attachment of a glucose unit to substrate **4** was confirmed by the presence of a one-proton doublet from proton 1″ at the anomeric carbon atom at δ = 4.39 ppm in the ^1^H-NMR spectrum with the coupling constant (*J* = 7.7 Hz) characteristic for its *β*-configuration (Supplementary Materials: Figure S25). A three-proton singlet at δ = 3.51 ppm in the ^1^H NMR spectrum with the corresponding signal at δ = 60.5 ppm in the ^13^C-NMR spectrum evidenced the presence of the -OCH_3_ moiety (Supplementary Materials: Figures S25 and S28). Based on the HMBC experiment, we concluded that *O*-methylation occurred at the C-4″ hydroxyl group of the sugar moiety, because the proton signal due to -OCH_3_ was correlated with the signal of C-4″ (about δ = 80 ppm) (Supplementary Materials: Figure S37). The position of the substitution with the glucose unit was established as C-4′-CH_3_ because the proton singlet of the methyl group at C-4′ (δ = 2.36 ppm) disappeared in product **4a**, and two one-proton signals from the –CH_2_ moiety appeared in the ^1^H NMR spectrum at δ = 4.92 ppm (*J* = 12.3 Hz) and δ = 4.66 ppm (*J* = 12.3 Hz) with the corresponding carbon signal at δ = 70.6 ppm in the ^13^C NMR spectrum (Supplementary Materials: Figures S23, S25, and S28). Furthermore, the signals from the flavanone A-ring remained intact. The characteristic AA’BB’ coupling system with the signals from protons at C-2′, C-6′ and signals from protons at C-3′, C-5′ only slightly shifted, which confirmed substitution at C-4′-CH_3_ (Supplementary Materials: Figures S24 and S27). Additionally, in the HMBC experiment, the proton at the anomeric carbon atom (δ = 4.39 ppm) was coupled with the C-4′-CH_2_ signal (δ = 70.6 ppm) (Supplementary Materials: Figure S37).

**Scheme 4:**
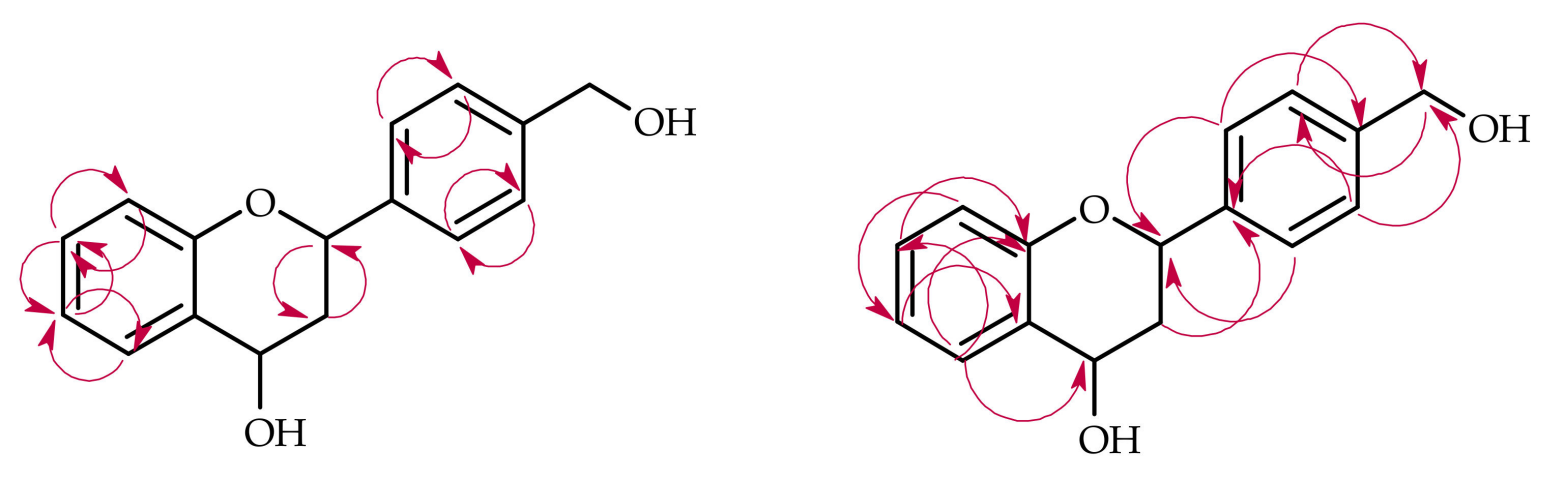
Key COSY (**on the left**) and HMBC (**on the right**) correlations for the structure elucidation of product **4b**.

The structure of product **4b** was established as 2-phenyl-(4′-hydroxymethyl)-4-hydroxychromane (Scheme 4). The disappearance of the 4′-methyl group signal at 2.36 ppm and the appearance of the methylene group signal at 4.66 ppm in the ^1^H NMR spectrum proved that substitution occurred at 4′-CH_3_ of the substrate **4** (Supplementary Materials: Figure S46). In addition, the signal of two protons at C-3′ and C-5′ shifted towards the lower field from δ = 7.26 ppm (in **4**) to δ = 7.41 ppm (in **4b**) (Supplementary Materials: Figures S4 and S45). In the HMBC experiment, the protons at C-4′-CH_2_- coupled with the signal from C-3′, C-5′, which also confirmed substitution at 4′-CH_3_ (Supplementary Materials: Figure S56). However, the shifts of all the A-ring protons and 3_ax_ (pseudo-axial) and 3_eq_ (pseudo-equatorial) protons indicated another change that occurred in the C-ring at C-4. A triplet appeared in the ^1^H NMR spectrum at δ = 4.78 ppm, indicating that the carbonyl group at C-4 had reduced. As a result, the signals from two protons at C-3 shifted towards the higher field from δ = 3.15 ppm (3_ax_ in **4**) and δ = 2.83 ppm (3_eq_ in **4**) to δ = 2.21 ppm (3_ax_ in **4b**) and δ = 2.12 ppm (3_eq_ in **4b**), which proved reduction reaction at C-4. Similarly, the signal from the proton at C-2 shifted slightly from δ = 5.59 ppm (in **4**) to δ = 5.30 ppm (in **4b**) (Supplementary Materials: Figures S3 and S44).

**Scheme 5:**
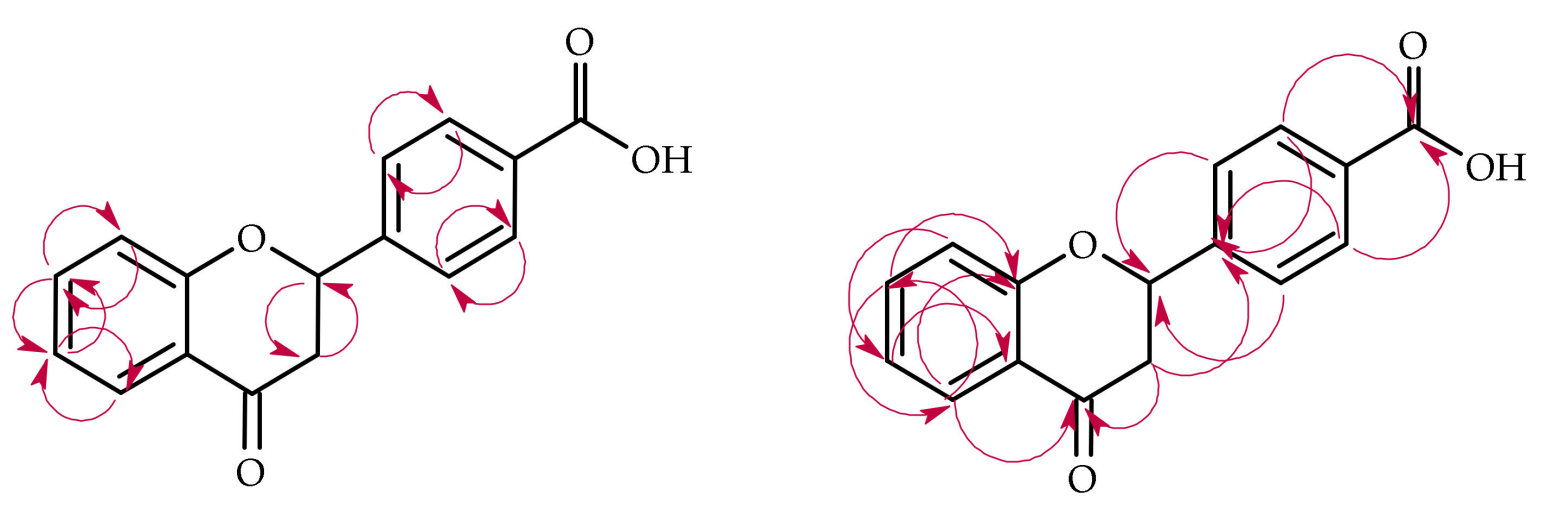
Key COSY (**on the left**) and HMBC (**on the right**) correlations for the structure elucidation of product **4c**.

Another biotransformation product was determined as flavanone 4′-carboxylic acid (Scheme 5). In the ^1^H NMR spectrum, the signal from the C-4′ methyl group was absent and no other signal appeared. Moreover, the signals from the protons at C-2′, C-6′ and C-3′, C-5′ were shifted towards the lower field from δ = 7.47 ppm (C-2′, C-6′ in **4**) and δ = 7.26 ppm (C-3′, C-5′ in **4**) to δ = 7.75 ppm (C-2′, C-6′ in **4c**) and δ = 8.12 ppm (C-3′, C-5′ in **4c**) (Supplementary Materials: Figures S4 and S65). Based on these data, it can be concluded that the C-4′ methyl group was oxidized to the C-4′ carboxylic group. Moreover, in the HMBC experiment, the signal from protons at C-3′, C-5′ correlated with the signal from carboxylic carbon at δ = 167.3 ppm, and the signal from protons at C-2′, C-6′ correlated with the signal from C-4′ at δ = 131.6 ppm (in **4c**), which was shifted from δ = 137.4 ppm (in **4**) (Supplementary Materials: Figures S7 and S75).

### 2.2. Biotransformation of 4′-Methylflavanone (***4***) in the Culture of B. bassiana KCH J1.5

**Scheme 6:**
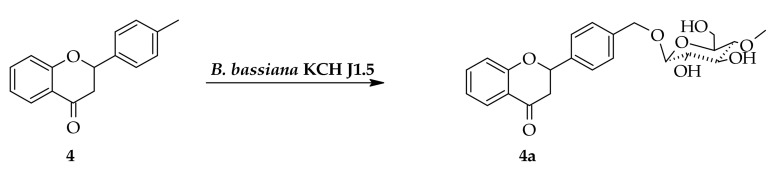
Microbial transformation of 4′-methylflavanone (**4**) in *B. bassiana* KCH J1.5 culture.

The biotransformation of 4′-methylflavanone (**4**) in the culture of *B. bassiana* KCH J1.5 resulted in the formation of the one main product, i.e., flavanone 4′-methylene-*O*-*β*-D-(4″-*O*-methyl)-glucopyranoside (**4a**) with 52.0% yield (47.0 mg), which was also obtained as the main product in the culture of *I. fumosorosea* KCH J2 (Scheme 6).

### 2.3. Biotransformation of 4′-Methylflavanone (***4***) in the Culture of I. farinosa KCH J2.1

**Scheme 7:**
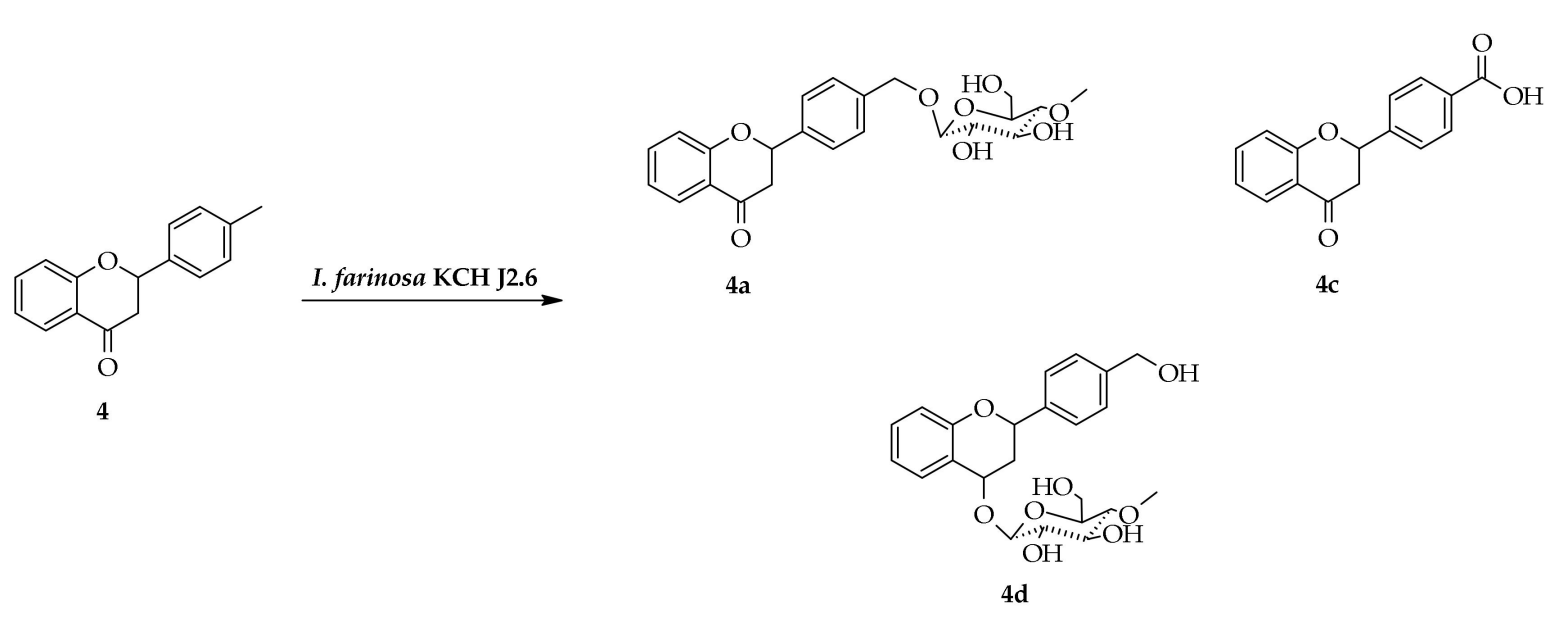
Microbial transformation of 4′-methylflavanone (**4**) in I. farinosa KCH J2.6 culture.

4′-Methylflavanone (**4**) was also used in the biotransformation performed in the culture of *I. farinosa* KCH J2.6. As a result, the following products were obtained: flavanone 4′-methylene-*O*-*β*-D-(4″-*O*-methyl)-glucopyranoside (**4a**) with a 15.3% yield (13.8 mg), flavanone 4′-carboxylic acid (**4c**) with a 6.6% yield (3.7 mg), and 4′-hydroxymethylflavanone 4*-O*-*β*-D-(4″-*O*-methyl)-glucopyranoside (**4d**) with 3.7% yield (3.4 mg) (Scheme 7).

**Scheme 8:**
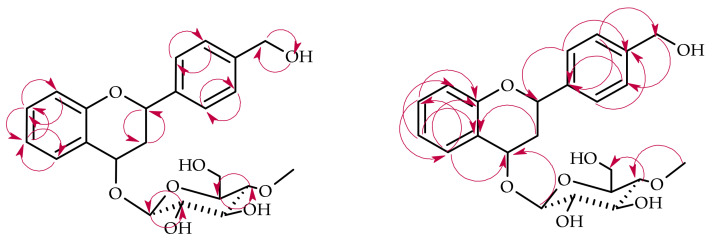
Key COSY (**on the left**) and HMBC (**on the right**) correlations for the structure elucidation of product **4d**.

The observed **4d** product had been 4″-*O*-methyl glycosylated (Scheme 8). The characteristic signals from the protons H2″–H6″ could be seen in the ^1^H NMR spectrum from about δ = 3.9 ppm to about δ = 3.1 ppm, as well as the corresponding carbon signals in the ^13^C-NMR from about δ = 80 ppm to about δ = 62 ppm (Supplementary Materials: Figures S86 and S89). A three-proton singlet at δ = 3.54 ppm in the ^1^H NMR spectrum with the corresponding signal at δ = 60.5 ppm in the ^13^C-NMR spectrum evidenced *O*-methylation, which occurred at the C-4″ hydroxyl group of the sugar moiety, because the proton signal due to -OCH_3_ correlated with the signal of C-4″ (about δ = 80 ppm) in the HMBC experiment (Supplementary Materials: Figures S86, S89, and S98). The attachment of a glucose unit to substrate **4** by the anomeric carbon atom C-1″ (at δ = 101.2 ppm in the ^13^C-NMR spectrum) was confirmed in the HMBC experiment because the signal from H-1″ correlated with the signal from flavonoid skeleton C-4. The coupling constant of one-proton doublet from proton at the anomeric carbon (*J* = 7.9 Hz) is characteristic of glucose *β*-configuration (Supplementary Materials: Figures S86, S89, and S98). In the ^1^H NMR spectrum, the protons of the A-ring and the C-ring were shifted, which pointed out the reduction of the carbonyl group at C-4. Moreover, at δ = 4.91 ppm a one-proton signal appeared, which corresponded with the C-4 signal at δ = 69.9 ppm in the HSQC (heteronuclear single quantum coherence) experiment (Supplementary Materials: Figures S86 and S95). Additionally, in the HMBC experiment, the signal from the proton at C-5 correlated with the signal from C-4 (Supplementary Materials: Figure S97). However, the absence of the C-4′-CH_3_ group signal, the presence of a new two-proton signal at δ = 4.65 ppm, and the shift of the signal from protons at C-3′, C-5′ indicated that C-4′-CH_3_ had been substituted with the hydroxyl moiety, similar to the product **4b** (Supplementary Materials: Figure S86). In the HMBC experiment, the protons at C-4′-CH_2_- coupled with the signal from C-3′, C-5′ also evidence substitution at 4′-CH_3_ (Supplementary Materials: Figure S98).

The presented studies were conducted to investigate the usability of selected strains of entomopathogenic filamentous fungi to biotransform 4′-methylflavanone into glycosylated and hydroxylated derivatives with potentially improved bioactivity and bioavailability. Our next aim is to run in vitro studies of the obtained compounds focused on their interaction with biological membranes, liposomes, and human albumin. These studies will be a continuation of our previous studies with 2′-hydroxy-4-methylchalcone, 4′-methylflavanone, and 4′-methylflavone, and will allow us to compare results for flavonoid aglycones and glycosides [26].

Both our previous and other teams’ studies showed that entomopathogenic filamentous fungi can be a valuable tool in the biotransformation of phenolic compounds, especially flavonoids, including 4″-*O*-methylglucosylation and hydroxylation [21,22,23,24,25,27,28,29,30,31,32,33].

**Scheme 9:**
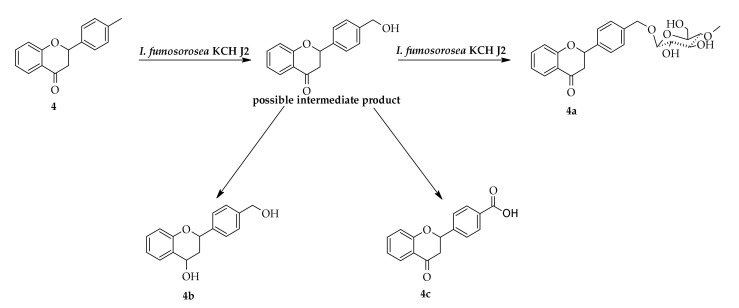
The probable course of 4′-methylflavanone (**4**) biotransformation in the culture of *I. fumosorosea* KCH J2.

The entomopathogenic filamentous fungi are known for their extensive enzymatic systems. For instance, *Beauveria bassiana* is able to perform reactions such as hydroxylation and glycosylation or deglycosylation, acetylation, and reduction, but most of the enzymes catalyzing these reactions have not been isolated and described. The glycosylated derivatives observed in this paper (**4a** and **4d**) were formed by the methyglucosylation functional module. Xie and coworkers identified a glycosyltransferase–methyltransferase gene pair that encodes these enzymes [34]. On the other hand, the observed enzymatic oxidations in products **4b** and **4c** were probably catalyzed by cytochrome P450 monooxygenases [35]. Our earlier studies revealed that *B. bassiana* KCH J1.5 enzymatic systems catalyze the hydroxylation and subsequent glycosylation of the methyl moiety at C-6 in the A ring of the flavanone skeleton [23]. However, it was not observed in the case of the methyl group at C-2′ in the B-ring [25]. The current studies showed that hydroxylation and subsequent glycosylation of the methyl group are possible at the C-4′ position of the B ring. Moreover, the product of these reactions, 4′-methylene-*O*-*β*-D-(4″-*O*-methyl)-glucopyranoside, was also observed in the post-reaction mixtures in the experiments with two other strains, *I. fumosorosea* KCH J2 and *I. farinosa* J2.6. Further analysis of the structures of microbial transformation products performed in the culture of *I. fumosorosea* KCH J2 allowed us to propose the probable course of reactions. It can be assumed that reaction of the hydroxylation at C-4′-CH_3_ occurred first and that this led to 4′-hydroxymethylflavanone. Subsequently, three different reaction pathways occurred. The first one led to the creation of 4″-O-methylglucosyl derivative after glycosylation of a newly formed hydroxyl group at C-4′-CH_3_ (**4a**—main product), the second one led to the formation of 2-phenyl-(4′-hydroxymethyl)-4-hydroxychromane (**4b**) with the reduced carbonyl group at C-4, and the third one resulted in the formation of flavanone 4′-carboxylic acid (**4c**)—a product of further oxidation of the hydroxyl group at C-4′-CH_3_. The products of the carbonyl group reduction were also observed in 2′-methoxyflavanone and 3′-methoxyflavanone biotransformations [30]. The probable course of 4′-methylflavanone biotransformation in the culture of *I. fumosorosea* KCH J2 is shown in Scheme 9.

**Scheme 10:**
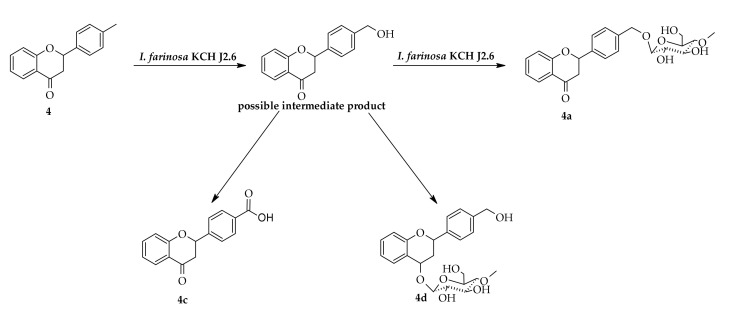
The probable course of 4′-methylflavanone (**4**) biotransformation in the culture of *I. farinosa* KCH J2.6.

In the case of microbial transformation in the culture of *B. bassiana* KCH J1.5 only one main product (**4a**) with a high isolated yield (52%) was observed, which suggests that glycosyltransferase, responsible for the glycosylation of the hydroxyl moiety, was very efficient. On the other hand, biotransformation of 4′-methylflavanone with the use of *I. farinosa* KCH J2.6 led to the formation of products **4a**, **4c**, and **4d**. It can be assumed that 4′-hydroxymethylflavanone can be a possible intermediate product, which was further transformed by the three competing enzymes into products **4a**, **4c,** and **4d**. The analogous products of glycosylation at C-4 by the strain *I. fumosorosea* KCH J2 enzymatic system were previously observed in the case of 6-methylflavanone and 2′-methylflavanone biotransformations [23,25]. The presumable course of 4′-methylflavanone biotransformation in the culture of *I. farinosa* KCH J2.6 is shown in Scheme 10.

### 2.4. Pharmacokinetics, Drug-Likeness, and Biological-Activity Prediction

#### 2.4.1. SwissADME

Pharmacokinetics, water solubility, and drug-likeness of 4′-methylflavanone (**4**) and its biotransformation products were predicted using the Swiss-ADME online tool (Supplementary Materials: Figures S17, S38, S58, S78, and S99). The brain or intestinal estimated permeation method (BOILED-Egg), as a predictive model that computes the lipophilicity and polarity of small molecules, showed that the gastrointestinal absorption of all molecules tested was high, which is crucial for any potential drug meant to be taken orally. The biotransformation products, **4a**, **4b**, **4c**, and **4d**, were more soluble in water than biotransformation substrate **4**. An especially high increase in aqueous solubility was observed for glycosylated products **4a** and **4d**. These biotransformation products did not show an ability to permeate passively through the blood–brain barrier but gained the ability to be actively transported by the P-glycoprotein. In contrast, compounds **4**, **4b**, and **4c** were predicted to permeate passively through the blood–brain barrier. Moreover, **4c** was predicted to be effluated from the central nervous system by P-glycoprotein. Biotransformation products **4a**, **4b**, **4c**, and **4d** did not inhibit cytochrome P450 enzymes involved in drug metabolism (CYP1A2, CYP2C9, CYP2C19, CYP2D6, and CYP3A4), which eliminates the risk of adverse drug effects when using these molecules as drugs [36]. All molecules tested passed Swiss-ADME platform drug-likeness estimators with zero violations. The Abbott bioavailability score (ABS), which is formulated as the probability that a compound will have >10% bioavailability in rats or measurable Caco-2 permeability, for all tested compounds was 0.55, except molecule **4c** showing ABS 0.85.

#### 2.4.2. Way2Drug Pass Online

The Way2Drug PASS Online and accompanying services, which predict biological activity, including pharmacological effects, mechanisms of action, interaction with metabolic enzymes, antimicrobial activity, etc., were used to assess the activity of 4′-methylflavanone and its biotransformation products (Supplementary Materials: Figures S18–S21, S39–S42, S52–S59, S79–S82, and S100–S103).

4′-Methylflavanone (**4**) could be used as a membrane integrity agonist (*Pa* = 0.927), a cytochrome P450 stimulant (*Pa* = 0.896), a mucomembranous protector (*Pa* = 0.842), or an antifungal agent against *Candida dubliniensis* (*Pa* = 0.709).

The predicted biological activities of flavanone 4′-methylene-*O*-*β*-D-(4″-*O*-methyl)-glucopyranoside (**4a**) include hepatoprotective (*Pa* = 0.910), antimicrobial as CDP-glycerol glycerophosphotransferase inhibitor (*Pa* = 0.900), antihypercholesterolemic (*Pa* = 0.881), anticarcinogenic (*Pa* = 0.866), and chemopreventive (*Pa* = 0.848).

2-Phenyl-(4′-hydroxymethyl)-4-hydroxychromane (**4b**) was predicted as a potential membrane integrity agonist (*Pa* = 0.908) and antifungal agent against *Candida dubliniensis* (*Pa* = 0.704). Its activity includes inhibition of enzymes such as pullulanase (*Pa* = 0.828), sugar-phosphatase (*Pa* = 0.815), and alkenylglycerophosphocholine hydrolase (*Pa* = 0.805).

Flavanone 4′-carboxylic acid (**4c**) is expected to act as membrane integrity agonist, and an antifungal agent against *Candida dubliniensis* (*Pa* = 0.652), and an antiviral agent against human immunodeficiency virus 2 (*Pa* = 0.729). This compound is highly likely to inhibit methylenetetrahydrofolate reductase (NADPH) (*Pa* = 0.899), 4-nitrophenol 2-monooxygenase (*Pa* = 0.884), and testosterone 17beta-dehydrogenase (NADP+) (*Pa* = 0.889).

The possible biological activity of 4′-hydroxymethylflavanone 4*-O*-*β*-D-(4″-*O*-methyl)-glucopyranoside (**4d**) was predicted as antimicrobial as CDP-glycerol glycerophosphotransferase inhibitor (*Pa* = 0.926) and replicase polyprotein 1ab from severe acute respiratory syndrome coronavirus-2 inhibitor (*Pa* = 0.817), and as anticarcinogenic (*Pa* = 0.881), antineoplastic (*Pa* = 0.838), hepatoprotective (*Pa* = 0.820), and chemopreventive (*Pa* = 0.807).

Membrane integrity is crucial for normal cell homeostasis and function [7]. The predicted role of 4′-methylflavanone (**4**), 2-phenyl-(4′-hydroxymethyl)-4-hydroxychromane (**4b**), and flavanone 4′-carboxylic acid (**4c**) as membrane integrity agonists seems to be justified by previous studies showing that the interaction between flavonoids and membranes could protect their integrity by reducing the oxidation of the membrane lipids [37,38]. In addition, our earlier in vitro studies also showed that 4′-methylflavanone (**4**) affects the hydrophilic region of the lipid–protein membrane (RBCMs) and the lipid model membrane (PC) [26].

Some previous studies have observed the protection of the mucomembrane by flavonoids other than 4′-methylflavanone (**4**), but the mechanism needs further investigation [39,40].

*Candida dubliniensis* is an opportunistic fungal pathogen that can become pathogenic when the host’s immune system fails and can seriously affect human health. The potential activity of 4′-methylflavanone (**4**), 2-phenyl-(4′-hydroxymethyl)-4-hydroxychromane (**4b**), and flavanone 4′-carboxylic acid (**4c**) as antifungal agents is promising, especially as the development of a new effective therapy for fungal diseases remains one of the major challenges of modern medicine [41].

The glycosylated derivatives of 4′-methylflavanone, i.e., 4′-methylene-*O*-*β*-D-(4″-*O*-methyl)-glucopyranoside (**4a**) and 4′-hydroxymethylflavanone 4*-O*-*β*-D-(4″-*O*-methyl)-glucopyranoside (**4d**) are supposed to exhibit hepatoprotective activity, which had been observed in many previous in vitro and in vivo experiments with various flavonoids. The flavanol glycosides from the leaves of *Cleome viscosa* L. showed hepatoprotective activity against CCl_4_-induced hepatotoxicity on HepG2 cells [42]. Hepatoprotective activity was also observed for many other natural extracts of flavonoids [11,12,43,44].

CDP-glycerol glycerophosphotransferase catalyzes the teichoic acid chain synthesis, a key component of the Gram-positive bacterial cell wall involved in its pathogenesis [45]. Since this synthesis is essential for bacterial cell wall integrity, it has become a potential antibacterial target. The predicted activities of 4′-methylene-*O*-*β*-D-(4″-*O*-methyl)-glucopyranoside (**4a**) (*Pa* = 0.900) and 4′-hydroxymethylflavanone 4*-O*-*β*-D-(4″-*O*-methyl)-glucopyranoside (**4d**) (*Pa* = 0.926) are much higher than those predicted for, e.g., procyanidin and catechin using the same PASS online tool [46].

Compounds **4a** and **4d** also have a high possibility of anticarcinogenic and chemopreventive activity. As reported by Forni, more than 23,000 scientific publications have described the potential anticancer activity of flavonoids. Many pieces of evidence confirm such activity. However, the mechanisms of action are still not fully understood [8].

## 3. Materials and Methods

### 3.1. Substrates

The biotransformation substrate, 4′-methylflavanone (**4**) was obtained by the chemical synthesis that was previously described by our team [26]. The physical data, including, color and form, melting point (°C), molecular ion mass, molecular formula, retention time t_R_ (min), retardation factor Rf, optical rotation ${\left[\alpha \right]}_{\;D}^{20}$ with concentration c (M), and NMR spectral data of the resulting compound **4** are presented in Table 1 and Table 2, and in the Supplementary Materials.

#### 4′-Methylflavanone (**4**)

Light-yellow crystals, mp = 61–62 °C, ESISMS *m*/*z* 239.1 ([M + H]^+^, C_16_H_14_O_2_), t_R_ = 17.51, Rf = 0.96,$\;{\left[\alpha \right]}_{\;D}^{20}$ = 0.0 (c = 1.22, acetone); ^1^H-NMR, see Table 1, ^13^C-NMR, see Table 2 and Supplementary Materials: Figures S1–S16.

### 3.2. Microorganisms

In the performed biotransformations, three strains of entomopathogenic filamentous fungi, *I. fumosorosea* KCH J2, *I. farinosa* J2.6, and *B. bassiana* KCH J1.5 were utilized. These microorganism strains were collected from the Department of Food Chemistry and Biocatalysis of the Wrocław University of Environmental and Life Sciences, Poland.

Our previous works have already provided a detailed description of fungal collection, propagation, and genetic identification [31,33]. The entomopathogenic filamentous fungi were maintained on potato slants at 4 °C and were subcultured before use in microbial transformations [31,33].

### 3.3. Analysis

Chromatographic methods (TLC and HPLC) were used to assess the course of the biotransformations. TLC analysis was carried out using TLC Silica gel 60/Kieselguhr F254 (0.2 mm thick) plates (Merck, Darmstadt, Germany) with a mixture of chloroform (Stanlab, Lublin, Poland) and methanol (Chempur, Piekary Śląskie, Poland) (9:1 *v*/*v*) as eluent. The products were observed without additional visualization under the ultraviolet lamp at λ = 254 nm and λ = 365 nm [24,25].

HPLC analyses were performed on a Dionex Ultimate 3000 instrument (Thermo Fisher Scientific, Waltham, MA, USA) with a DAD-3000 diode array detector using an analytical octadecylsilica (ODS) 2 column (4.6 mm × 250 mm, Waters, Milford, MA, USA) and pre-column. The mobile phase was a mixture of 0.1% aqueous acid formic acid *v*/*v* (A) and acetonitrile (B). The gradient program was as follows: initial conditions—32.5% B in A, 4 min—40% B in A, 8 min—40% B in A, 10 min—45% B in A, 15 min—95% B in A, 18 min—95% B in A, 19 min—32.5% B in A, and 23 min—32.5% B in A. The flow rate was 1 mL/min, the injection volume was 5 µL, and the detection wavelength was 280 nm [23].

Scale-up biotransformation product separation was attained using 500 µm and 1000 µm preparative TLC silica gel plates (Analtech, Gehrden, Germany) with a mixture of chloroform and methanol (9:1 *v*/*v*) as eluent. The compounds were extracted from the selected gel fractions using 20 mL of ethyl acetate (Stanlab, Lublin, Poland) three times, and the solvent was evaporated using a rotary evaporator [24,25].

NMR analyses (^1^H-NMR, ^13^C-NMR, COSY, heteronuclear single quantum correlation (HSQC), and HMBC) were performed using a DRX Avance^TM^ 600 MHz NMR spectrometer (Bruker, Billerica, MA, USA). The prepared samples of biotransformation substrates and products were dissolved in deuterated acetone.

Optical rotation was measured using a digital polarimeter P-2000-Na (ABL&E-JASCO, Kraków, Poland).

Molecular formulae of all products were confirmed by analysis performed on the LC–MS 8045 Shimadzu triple quadrupole liquid chromatograph mass spectrometer with electrospray ionization (ESI) source (Shimadzu, Kyoto, Japan), as described previously, with minor modifications [29]. The separation was achieved on the Kinetex column (2.6 µm C18 100 Å, 100 mm × 3 mm, Phenomenex, Torrance, CA, USA) operated at 30 °C. The mobile phase was a mixture of 0.1% aqueous formic acid *v*/*v* (A) and acetonitrile (B). The flow rate was 0.4 mL min^−1^ and the injection volume was 5 µL. The gradient program was as follows: initial conditions—80% B in A, 6.5 min—100% B, and 7 min—80% B in A. The principal operating parameters for the LC–MS were set as follows: nebulizing gas flow: 3 L min^−1^, heating gas flow: 10 L min^−1^, interface temperature: 300 °C, drying gas flow: 10 L min^−1^, data acquisition range, *m*/*z* 100–1000 Da; ionization mode, negative and positive. Data were collected with LabSolutions version 5.97 (Shimadzu, Kyoto, Japan) software [24,25].

### 3.4. Screening Procedure

The reason for performing biotransformations in the screening procedure was to assess the time needed for the complete conversion of substrate **4**. As a growth medium for entomopathogenic filamentous fungi, modified Sabouraud medium (10g aminobac (purchased from BTL, Warsaw, Poland), 30 g saccharose (purchased from Chempur, Piekary Śląskie, Poland), and 1 L distilled water) was used. Firstly, the cultures of the microorganisms strains were transferred from the potato slants to 300 mL Erlenmeyer flasks with 100 mL modified Sabouraud liquid medium. The cultures were bred on a rotary shaker (DHN, Warsaw, Poland) (140 rpm) at 25 °C for 72 h. Secondly, 0.5 mL of the pre-grown cultures were transferred to another 300 mL Erlenmeyer flasks with 100 mL modified Sabouraud liquid medium and also incubated at 25 °C for 72 h. Afterwards, 10 mg of substrate **4** (dissolved in 0.5 mL of dimethyl sulfoxide (Chempur, Piekary Śląskie, Poland)) was added to each flask with entomopathogenic filamentous fungi. The molar concentration of substrates **4** was 0.42 mM. The samples were collected after 3, 6, and 9 days of substrate incubation and extracted with 30 mL of ethyl acetate. Subsequently, the extracts were dried for 5 min with anhydrous magnesium sulfate (Chempur, Piekary Śląskie, Poland) and concentrated using a rotary evaporator (Heidolph, Schwabach, Germany) at 55 °C. All biotransformations were terminated after the confirmation of complete substrate conversion or lack of further substrate conversion, which occurred after 9 days of the experiment. The collected samples were analyzed by TLC and HPLC methods to check substrate conversion and the appearance of flavonoid products (visible on the TLC plates under an ultraviolet lamp). At the same time, the stability of the substrate under biotransformation conditions was investigated. Microorganism cultivation with no substrate added was performed as a negative control [24,25].

### 3.5. The Semipreparative Biotransformations

The scale of the semipreparative biotransformation was chosen to obtain products in amounts sufficient to perform spectroscopic analyses and to determine chemical structures. Because of that, for each biotransformation, a 2 L flask with 500 mL of the modified Sabouraud medium was used [24,25].

In the first step, 1 mL of the preincubation culture of the microorganism was transferred to the flask and then incubated for 72 h under the same conditions as during the screening procedure. In the next step, 50 mg of substrate **4** (dissolved in 1.5 mL of dimethyl sulfoxide) was added to the flask. The molar concentration of substrate **4** was 0.42 mM. The incubation of the biotransformation mixture on the rotary shaker was continued to the 9th day. After confirming complete substrate conversion (or lack of further substrate conversion), the experiment was ended. The post-reaction mixture (mostly consisting of flavonoid products) was extracted three times with 300 mL portions of ethyl acetate. Afterwards, the joined extracts were dried for 5 min with anhydrous magnesium sulfate, filtered, and dried on a rotary evaporator. The separation and purification of the biotransformation products were achieved using preparative TLC plates. The product fractions, which were visible under an ultraviolet lamp, were marked, separated, extracted (three times with portions of 20 mL of the ethyl acetate), and finally analyzed by spectroscopic methods. The biotransformation yields were determined based on the masses of the isolated products [24,25].

The physical data, including color and form, melting point (°C), molecular ion mass, molecular formula, retention time t_R_ (min), retardation factor Rf, optical rotation ${\left[\alpha \right]}_{\;D}^{20}$ with concentration c (M), and NMR spectral data of the resulting compounds **4a**–**4d** are presented below, in Table 1 and Table 2, and in the Supplementary Materials.

#### 3.5.1. Flavanone 4′-methylene-O-*β*-D-(4″-O-methyl)-glucopyranoside (**4a**)

Light-yellow crystals, mp = 75–77 °C, ESIMS *m*/*z* 431.3 ([M + H]^+^, C_23_H_26_O_8_), t_R_ = 6.17, Rf = 0.61,$\;{\left[\alpha \right]}_{\;D}^{20}$ = −27.7 (c = 1.82, acetone); ^1^H-NMR, see Table 1, ^13^C-NMR, see Table 2, Supplementary Materials: Figures S22–S37.

#### 3.5.2. 2-Phenyl-(4′-hydroxymethyl)-4-hydroxychromane (**4b**)

Light-yellow crystals, mp = 171–172 °C, ESIMS *m*/*z* 298.3 (adduct [M + 31 + H]^+^ ([M + CH_3_CN + H]^+^), C_16_H_16_O_3_), t_R_ = 7.15, Rf = 0.80, ${\left[\alpha \right]}_{\;D}^{20}$ = −4.7 (c = 0.30, acetone); ^1^H-NMR, see Table 1, ^13^C-NMR, see Table 2, Supplementary Materials: Figures S43–S57.

#### 3.5.3. Flavanone 4′-carboxylic Acid (**4c**)

Light-yellow crystals, mp = 193–195 °C, ESIMS *m*/*z* 269.0 ([M + H]^+^, C_16_H_12_O_4_), t_R_ = 12.80, Rf = 0.75, ${\left[\alpha \right]}_{\;D}^{20}$ = 3.1 (c = 0.34, acetone); ^1^H-NMR, see Table 1, ^13^C-NMR, see Table 2, Supplementary Materials: Figures S63–S77.

#### 3.5.4. 4′-Hydroxymethylflavanone 4-O-*β*-D-(4″-O-methyl)-glucopyranoside (**4d**)

Light-yellow crystals, mp = 204–206 °C ESIMS *m*/*z* 479.3 (adduct [M + H]^+^, ([M + HCOOH + H]^+^), C_23_H_28_O_8_), t_R_ = 4.37, Rf = 0.47, ${\left[\alpha \right]}_{\;D}^{20}$ = 4.0 (c = 0.27, acetone); ^1^H-NMR, see Table 1, ^13^C-NMR, see Table 2, Supplementary Materials: Figures S83–S98.

### 3.6. Pharmacokinetics, Drug-Likeness, Biological Activity Prediction

The evaluation of general pharmacokinetic and physicochemical properties, medicinal chemistry friendliness, and potential biological activity for small molecules based on their structural formulae was performed with two computer tools, i.e., SwissADME and Way2Drug Pass Online with accompanying services. All the structures of the biotransformation substrate and products were built by ACD Chemsketch 2021.2.0 and saved in a .mol format, which can be imported into the SwissADME and Pass Online. The presented list of biological activity types in Pass Online is shown as the probability to be revealed (*Pa*) and not to be revealed (*Pi*). These values are independent and range from 0 to 1. The prediction results are shown in the Supplementary Materials: Figures S17–S21, S38–S42, S58–S62, S78–S82, and S99–S103.

## 4. Conclusions

In the presented study, 4′-methylflavanone was synthesized and biotransformed in the cultures of three entomopathogenic filamentous fungi strains, i.e., *I. fumosorosea* KCH J2, *B. bassiana* KCH J1.5, and *I. farinosa* KCH J2.1. The first strain used, *I. fumosorosea* KCH J2, was able to hydroxylate 4′-methylflavanone at C-4′-CH_3_ and reduce the carbonyl group at C-4. In the post-reaction mixture, a product of further oxidation of the hydroxyl group at C-4′-CH_3_—flavanone 4′-carboxylic acid was also observed. The main biotransformation product was 4″-*O*-methylglycosylated at C-4′-CH_3_. It can be assumed that the formation of this product was preceded by the intermediate product—4′-hydroxymethylflavanone.

The strain *B. bassiana* KCH J1.5 glycosylated 4′-methylflavanone at C-4′-CH_3_ with a very high isolated yield—52%. The last strain *I. farinosa* KCH J2.1 formed two glycosylated products, i.e., flavanone 4′-methylene-*O*-*β*-D-(4″-*O*-methyl)-glucopyranoside and 4′-hydroxymethylflavanone 4*-O*-*β*-D-(4″-*O*-methyl)-glucopyranoside. Furthermore, in the biotransformation mixture flavanone 4′-carboxylic acid was also identified.

The three obtained biotransformation products, i.e., flavanone 4′-methylene-*O*-*β*-D-(4″-*O*-methyl)-glucopyranoside, 2-phenyl-(4′-hydroxymethyl)-4-hydroxychromane, and 4′-hydroxymethylflavanone 4*-O*-*β*-D-(4″-*O*-methyl)-glucopyranoside have not been described in the literature until now. The Swiss-ADME simulations confirmed the increased water solubility and high gastrointestinal absorption of these compounds. The analyses using the Way2Drug Pass Online tool for biological activity prediction showed that both flavonoid derivatives exhibit great potential as anticarcinogen, hepatoprotectant, antimicrobial, and chemopreventive agents. However, the biological activity and bioavailability of these compounds require further research.

## Figures and Tables

**Table 1:** ^1^H-NMR chemical shifts δ (ppm) and coupling constants *J* (Hz) of 4′-methylflavanone (**4**) and products of its biotransformations, **4a**–**4d**, in acetone-d6, 600 MHz (Supplementary Materials: Figures S3, S4, S23–S25, S44–S46, S64, S65, and S84–S86).

Proton	Compound				
	4	4a	4b	4c	4d
H-2	5.59 (dd) *J* = 13.0, *J* = 2.8	5.63 (dd) *J* = 12.9, *J* = 2.9	5.30 (dd) *J* = 11.7, *J* = 2.2	5.77 (dd) *J* = 13.1, *J* = 2.9	5.30 (dd) *J* = 12.0, *J* = 1.5
H-3_ax_	3.15 (dd) *J* = 16.7, *J* = 13.0	3.14 (m)	2.21 (dt) *J* = 14.0, *J* = 2.4	3.16 (dd) *J* = 16.8, *J* = 13.1	2.45 (dt) *J* = 14.5, *J* = 2.0
H-3_eq_	2.83 (dd) *J* = 16.7, *J* = 2.6	2.86 (dd) *J* = 16.8, *J* = 3.0	2.12 (ddd) *J* = 14.1, *J* = 11.7, *J* = 3.5	2.94 (dd) *J* = 16.7, *J* = 3.0	2.01 (dd) *J* = 3.1, *J* = 2.5
H-4	-	-	4.78 (t) *J* = 2.9	-	4.91 (t) *J* = 2.6
H-5	7.84 (dd) *J* = 7.8, *J* = 1.2	7.84 (dd) *J* = 7.8, *J* = 1.7	7.35 (dd) *J* = 7.6, *J* = 1.6	7.86 (dd) *J* = 7.7, *J* = 1.8	7.44 (dd) *J* = 7.7, *J* = 1.6
H-6	7.08 (dd) *J* = 14.0, *J* = 7.8	7.09 (m)	6.91 (td) *J* = 7.5, *J* = 1.1	7.12 (m)	6.92 (td) *J* = 7.5, *J* = 1.1
H-7	7.57 (m)	7.58 (m)	7.20 (ddd) *J* = 8.4, *J* = 7.4, *J* = 1.7	7.61 (ddd) *J* = 8.8, *J* = 7.2, *J* = 1.7	7.23 (m)
H-8	7.08 (dd) *J* = 14.0, *J* = 7.8	7.09 (m)	6.86 (dd) *J* = 8.4, *J* = 0.8	7.12 (m)	6.87 (dd) *J* = 8.2, *J* = 0.7
H-2′	7.47 (d) *J* = 8.0	7.56 (d) *J* = 7.9	7.45 (d) *J* = 8.1	7.75 (d) *J* = 8.2	7.46 (d) *J* = 8.0
H-3′	7.26 (d) *J* = 7.9	7.49 (d) *J* = 8.1	7.41 (d) *J* = 8.2	8.12 (d) *J* = 8.4	7.40 (d) *J* = 8.1
H-5′	7.26 (d) *J* = 7.9	7.49 (d) *J* = 8.1	7.41 (d) *J* = 8.2	8.12 (d) *J* = 8.4	7.40 (d) *J* = 8.1
H-6′	7.47 (d) *J* = 8.0	7.56 (d) *J* = 7.9	7.45 (d) *J* = 8.1	7.75 (d) *J* = 8.2	7.46 (d) *J* = 8.0
H-1″	-	4.39 (d) *J* = 7.7	-	-	4.62 (d) *J* = 7.9
H-2″	-	3.27 (m)	-	-	3.21 (td) *J* = 8.8, *J* = 4.0
H-3″	-	3.52 (m)	-	-	3.56 (dd) *J* = 5.9, *J* = 2.6
H-4″	-	3.14 (m)	-	-	3.13 (dd) *J* = 9.5, *J* = 9.0
H-5″	-	3.27 (m)	-	-	3.35 (m)
H-6″	-	3.82 (dd) *J* = 11.6, *J* = 1.1 3.67 (dd) *J* = 11.6, *J* = 5.1	-	-	3.86 (dt) *J* = 8.3, *J* = 6.0, 3.70 (m)
C4″-OCH_3_	-	3.53 (s)	-	-	3.54 (s)
C4′-CH_3_	2.36 (s)	-	-	-	-
2″-OH	-	-	-	-	4.54 (d) *J* = 4.0
3″-OH	-	-	-	-	4.27 (d) *J* = 3.8
4′-CH_2_-	-	4.92 (d) *J* = 12.3 4.66 (d) *J* = 12.3	4.66 (s)	-	4.65 (d) *J* = 5.5
4′-CH_2_-OH		-	-	-	4.25 (t) *J* = 5.7

**Table 2:** ^13^C-NMR chemical shifts δ (ppm) and coupling constants *J* (Hz) of 4′-methylflavanone (**4**) and products of its biotransformations, **4a**–**4d,** in acetone-d6, 151 MHz (Supplementary Materials: Figures S6, S7, S26–S28, S47, S48, S66, S67, and S87–S89).

Carbon	Compound				
	4	4a	4b	4c	4d
C-2	80.3	80.2	73.8	79.9	73.8
C-3	44.9	44.9	39.7	44.9	36.2
C-4	191.9	191.9	63.7	191.4	69.9
C-4a	122.0	121.9	125.7	122.0	122.7
C-5	127.4	127.4	131.5	127.4	132.8
C-6	122.2	122.2	121.1	122.5	121.0
C-7	136.8	136.9	129.9	137.0	130.3
C-8	118.9	118.9	117.6	119.0	117.5
C-8a	162.5	162.4	155.8	162.2	156.3
C-1′	139.1	139.4	143.1	145.3	141.1
C-2′	127.3	127.2	127.0	127.3	127.4
C-3′	130.1	128.8	127.5	130.8	126.9
C-4′	137.4	139.7	141.2	131.6	143.1
C-5′	130.1	128.8	127.5	130.8	126.9
C-6′	127.3	127.2	127.0	127.3	127.4
C-1″	-	103.1	-	-	101.2
C-2″	-	75.2	-	-	75.0
C-3″	-	78.0	-	-	78.1
C-4″	-	80.5	-	-	80.6
C-5″	-	76.9	-	-	77.0
C-6″	-	62.4	-	-	62.6
4″-OCH_3_	-	60.5	-	-	60.5
4′-CH_3_	21.2	-	-	-	-
4′-CH_2_-	-	70.6	64.4	-	64.5
4′-COOH	-	-	-	167.3	-

## Data Availability

Samples of the compounds **4** and **4a**–**4d** are available from the authors.

## Supplementary Materials

The following supporting information can be downloaded at: https://www.mdpi.com/article/10.3390/ijms23105373/s1.
