# Supplementary material for: 4′-Methylflavanone Glycosides Obtained Using Biotransformation in the Entomopathogenic Filamentous Fungi Cultures as Potential Anticarcinogenic, Antimicrobial, and Hepatoprotective Agents
Source: Int J Mol Sci. 2022 May 11;23(10):5373. doi: 10.3390/ijms23105373 (PMC9140535; doi:10.3390/ijms23105373)
Supplement: Supplementary file 1 [file ijms-23-05373-s001.zip › ijms-1702503-supplementary.pdf]

Supplementary materials

4′-Methylflavanone glycosides obtained using biotransformation in the entomopathogenic filamentous fungi cultures as potential anticarcinogenic, antimicrobial, and hepatoprotective agents

Agnieszka Krawczyk-Łebek*, Monika Dymarska, Tomasz Janeczko and Edyta Kostrzewa-Susłow*

Department of Food Chemistry and Biocatalysis, Faculty of Biotechnology and Food Science, Wrocław University of Environmental and Life Sciences, Wrocław, Poland

*Correspondence: agnieszka.krawczyk-lebek@upwr.edu.pl, edyta.kostrzewa-suslow@upwr.edu.pl

Content

Figure S1. MS analysis of 4′-methylflavanone (**4**)
Figure S2. ^{1}H NMR spectrum (*δ,* acetone-d6, 600 MHz) of 4′-methylflavanone (**4**)
Figure S3. ^{1}H NMR spectrum expansion (*δ,* acetone-d6, 600 MHz) of 4′-methylflavanone (**4**)
Figure S4. ^{1}H NMR spectrum expansion (*δ,* acetone-d6, 600 MHz) of 4′-methylflavanone (**4**)
Figure S5. ^{13}C NMR spectrum (*δ,* acetone-d6, 151 MHz) of 4′-methylflavanone (**4**)
Figure S6. ^{13}C NMR spectrum expansion (*δ,* acetone-d6, 151 MHz) of 4′-methylflavanone (**4**)
Figure S7. ^{13}C NMR spectrum expansion (*δ,* acetone-d6, 151 MHz) of 4′-methylflavanone (**4**)
Figure S8. COSY contour map – ^{1}H x ^{1}H of 4′-methylflavanone (**4**)
Figure S9. COSY contour map – ^{1}H x ^{1}H expansion of 4′-methylflavanone (**4**)
Figure S10. COSY contour map – ^{1}H x ^{1}H expansion of 4′-methylflavanone (**4**)
Figure S11. HSQC contour map – ^{1}H x ^{13}C of 4′-methylflavanone (**4**)
Figure S12. HSQC contour map – ^{1}H x ^{13}C expansion of 4′-methylflavanone (**4**)
Figure S13. HSQC contour map – ^{1}H x ^{13}C expansion of 4′-methylflavanone (**4**)
Figure S14. HMBC contour map – ^{1}H x ^{13}C of 4′-methylflavanone (**4**)
Figure S15. HMBC contour map – ^{1}H x ^{13}C expansion of 4′-methylflavanone (**4**)
Figure S16. HMBC contour map – ^{1}H x ^{13}C expansion of 4′-methylflavanone (**4**)
Figure S17. 4′-Methylflavanone (**4**) physicochemical and ADME parameters prediction using the SwissADME modelling
Figure S18. 4′-Methylflavanone (**4**) biological activity prediction using the Way2Drug Pass online modelling
Figure S19. 4′-Methylflavanone (**4**) antibacterial activity prediction using the Way2Drug AntiBac-Pred modelling
Figure S20. 4′-Methylflavanone (**4**) antifungal activity prediction using the Way2Drug AntiFun-Pred modelling
Figure S21. 4′-Methylflavanone (**4**) antiviral activity prediction using the Way2Drug AntiVir-Pred modelling
Figure S22. MS analysis of flavanone 4′-methylene-*O*-*β*-D-(4′′-*O*-methyl)-glucopyranoside (**4a**)

Figure S23. ^{1}H NMR spectrum (*δ,* acetone-d6, 600 MHz) of flavanone 4′-methylene-*O*-*β*-D-(4′′-*O*-methyl)-glucopyranoside (**4a**)
Figure S24. ^{1}H NMR spectrum expansion (*δ,* acetone-d6, 600 MHz) of flavanone 4′-methylene-*O*-*β*-D-(4′′-*O*-methyl)-glucopyranoside (**4a**)
Figure S25. ^{1}H NMR spectrum expansion (*δ,* acetone-d6, 600 MHz) of flavanone 4′-methylene-*O*-*β*-D-(4′′-*O*-methyl)-glucopyranoside (**4a**)
Figure S26. ^{13}C NMR spectrum (*δ,* acetone-d6, 151 MHz) of flavanone 4′-methylene-*O*-*β*-D-(4′′-*O*-methyl)-glucopyranoside (**4a**)
Figure S27. ^{13}C NMR spectrum expansion (*δ,* acetone-d6, 151 MHz) of flavanone 4′-methylene-*O*-*β*-D-(4′′-*O*-methyl)-glucopyranoside (**4a**)
Figure S28. ^{13}C NMR spectrum expansion (*δ,* acetone-d6, 151 MHz) of flavanone 4′-methylene-*O*-*β*-D-(4′′-*O*-methyl)-glucopyranoside (**4a**)
Figure S29. COSY contour map – ^{1}H x ^{1}H of flavanone 4′-methylene-*O*-*β*-D-(4′′-*O*-methyl)-glucopyranoside (**4a**)
Figure S30. COSY contour map – ^{1}H x ^{1}H expansion of flavanone 4′-methylene-*O*-*β*-D-(4′′-*O*-methyl)-glucopyranoside (**4a**)
Figure S31. COSY contour map – ^{1}H x ^{1}H expansion of flavanone 4′-methylene-*O*-*β*-D-(4′′-*O*-methyl)-glucopyranoside (**4a**)
Figure S32. HSQC contour map – ^{1}H x ^{13}C of flavanone 4′-methylene-*O*-*β*-D-(4′′-*O*-methyl)-glucopyranoside (**4a**)
Figure S33. HSQC contour map – ^{1}H x ^{13}C expansion of flavanone 4′-methylene-*O*-*β*-D-(4′′-*O*-methyl)-glucopyranoside (**4a**)
Figure S34. HSQC contour map – ^{1}H x ^{13}C expansion of flavanone 4′-methylene-*O*-*β*-D-(4′′-*O*-methyl)-glucopyranoside (**4a**)
Figure S35. HMBC contour map – ^{1}H x ^{13}C of flavanone 4′-methylene-*O*-*β*-D-(4′′-*O*-methyl)-glucopyranoside (**4a**)
Figure S36. HMBC contour map – ^{1}H x ^{13}C expansion of flavanone 4′-methylene-*O*-*β*-D-(4′′-*O*-methyl)-glucopyranoside (**4a**)
Figure S37. HMBC contour map – ^{1}H x ^{13}C expansion of flavanone 4′-methylene-*O*-*β*-D-(4′′-*O*-methyl)-glucopyranoside (**4a**)
Figure S38. Flavanone 4′-methylene-*O*-*β*-D-(4′′-*O*-methyl)-glucopyranoside (**4a**) physicochemical and ADME parameters prediction using the SwissADME modelling
Figure S39. Flavanone 4′-methylene-*O*-*β*-D-(4′′-*O*-methyl)-glucopyranoside (**4a**) biological activity prediction using the Way2Drug Pass online modelling
Figure S40. Flavanone 4′-methylene-*O*-*β*-D-(4′′-*O*-methyl)-glucopyranoside (**4a**) antibacterial activity prediction using the Way2Drug AntiBac-Pred modelling
Figure S41. Flavanone 4′-methylene-*O*-*β*-D-(4′′-*O*-methyl)-glucopyranoside (**4a**) antifungal activity prediction using the Way2Drug AntiFun-Pred modelling
Figure S42. Flavanone 4′-methylene-*O*-*β*-D-(4′′-*O*-methyl)-glucopyranoside (**4a**) antiviral activity prediction using the Way2Drug AntiVir-Pred modelling
Figure S43. MS analysis of 2-phenyl-(4′-hydroxymethyl)-4-hydroxychromane (**4b**)

Figure S44. ^{1}H NMR spectrum (*δ,* acetone-d6, 600 MHz) of 2-phenyl-(4′-hydroxymethyl)-4-hydroxychromane (**4b**)
Figure S45. ^{1}H NMR spectrum expansion (*δ,* acetone-d6, 600 MHz) of 2-phenyl-(4′-hydroxymethyl)-4-hydroxychromane (**4b**)
Figure S46. ^{1}H NMR spectrum expansion (*δ,* acetone-d6, 600 MHz) of 2-phenyl-(4′-hydroxymethyl)-4-hydroxychromane (**4b**)
Figure S47. ^{13}C NMR spectrum (*δ,* acetone-d6, 151 MHz) of 2-phenyl-(4′-hydroxymethyl)-4-hydroxychromane (**4b**)
Figure S48. ^{13}C NMR spectrum expansion (*δ,* acetone-d6, 151 MHz) of 2-phenyl-(4′-hydroxymethyl)-4-hydroxychromane (**4b**)
Figure S49. COSY contour map – ^{1}H x ^{1}H of 2-phenyl-(4′-hydroxymethyl)-4-hydroxychromane (**4b**)
Figure S50. COSY contour map – ^{1}H x ^{1}H expansion of 2-phenyl-(4′-hydroxymethyl)-4-hydroxychromane (**4b**)
Figure S51. COSY contour map – ^{1}H x ^{1}H expansion of 2-phenyl-(4′-hydroxymethyl)-4-hydroxychromane (**4b**)
Figure S52. HSQC contour map – ^{1}H x ^{13}C of 2-phenyl-(4′-hydroxymethyl)-4-hydroxychromane (**4b**)
Figure S53. HSQC contour map – ^{1}H x ^{13}C expansion of 2-phenyl-(4′-hydroxymethyl)-4-hydroxychromane (**4b**)
Figure S54. HSQC contour map – ^{1}H x ^{13}C expansion of 2-phenyl-(4′-hydroxymethyl)-4-hydroxychromane (**4b**)
Figure S55. HMBC contour map – ^{1}H x ^{13}C 2-phenyl-(4′-hydroxymethyl)-4-hydroxychromane (**4b**)
Figure S56. HMBC contour map – ^{1}H x ^{13}C expansion of 2-phenyl-(4′-hydroxymethyl)-4-hydroxychromane (**4b**)
Figure S57. HMBC contour map – ^{1}H x ^{13}C expansion of 2-phenyl-(4′-hydroxymethyl)-4-hydroxychromane (**4b**)
Figure S58. 2-Phenyl-(4′-hydroxymethyl)-4-hydroxychromane (**4b**) physicochemical and ADME parameters prediction using the SwissADME modelling
Figure S59. 2-Phenyl-(4′-hydroxymethyl)-4-hydroxychromane (**4b**) biological activity prediction using the Way2Drug Pass online modelling
Figure S60. 2-Phenyl-(4′-hydroxymethyl)-4-hydroxychromane (**4b**) antibacterial activity prediction using the Way2Drug AntiBac-Pred modelling
Figure S61. 2-Phenyl-(4′-hydroxymethyl)-4-hydroxychromane (**4b**) antifungal activity prediction using the Way2Drug AntiFun-Pred modelling
Figure S62. 2-Phenyl-(4′-hydroxymethyl)-4-hydroxychromane (**4b**) antiviral activity prediction using the Way2Drug AntiVir-Pred modelling
Figure S63. MS analysis of flavanone 4′-carboxylic acid (**4c**)
Figure S64. ^{1}H NMR spectrum (*δ,* acetone-d6, 600 MHz) of flavanone 4′-carboxylic acid (**4c**)
Figure S65. ^{1}H NMR spectrum expansion (*δ,* acetone-d6, 600 MHz) of flavanone 4′-carboxylic acid (**4c**)

Figure S66. ^{13}C NMR spectrum (*δ,* acetone-d6, 151 MHz) of flavanone 4′-carboxylic acid (**4c**)
Figure S67. ^{13}C NMR spectrum expansion (*δ,* acetone-d6, 151 MHz) of flavanone 4′-carboxylic acid (**4c**)
Figure S68. COSY contour map – ^{1}H x ^{1}H of flavanone 4′-carboxylic acid (**4c**)
Figure S69. COSY contour map – ^{1}H x ^{1}H expansion of flavanone 4′-carboxylic acid (**4c**)
Figure S70. COSY contour map – ^{1}H x ^{1}H expansion of flavanone 4′-carboxylic acid (**4c**)
Figure S71. HSQC contour map – ^{1}H x ^{13}C of flavanone 4′-carboxylic acid (**4c**)
Figure S72. HSQC contour map – ^{1}H x ^{13}C expansion of flavanone 4′-carboxylic acid (**4c**)
Figure S73. HSQC contour map – ^{1}H x ^{13}C expansion of flavanone 4′-carboxylic acid (**4c**)
Figure S74. HMBC contour map – ^{1}H x ^{13}C of flavanone 4′-carboxylic acid (**4c**)
Figure S75. HMBC contour map – ^{1}H x ^{13}C expansion of flavanone 4′-carboxylic acid (**4c**)
Figure S76. HMBC contour map – ^{1}H x ^{13}C expansion of flavanone 4′-carboxylic acid (**4c**)
Figure S77. HMBC contour map – ^{1}H x ^{13}C expansion of flavanone 4′-carboxylic acid (**4c**)
Figure S78. Flavanone 4′-carboxylic acid (**4c**) physicochemical and ADME parameters prediction using the SwissADME modelling
Figure S79. Flavanone 4′-carboxylic acid (**4c**) biological activity prediction using the Way2Drug Pass online modelling
Figure S80. Flavanone 4′-carboxylic acid (**4c**) antibacterial activity prediction using the Way2Drug AntiBac-Pred modelling
Figure S81. Flavanone 4′-carboxylic acid (**4c**) antifungal activity prediction using the Way2Drug AntiFun-Pred modelling
Figure S82. Flavanone 4′-carboxylic acid (**4c**) antiviral activity prediction using the Way2Drug AntiVir-Pred modelling
Figure S83. MS analysis of and 4′-hydroxymethylflavanone 4-*O*-*β*-D-(4′′-*O*-methyl)-glucopyranoside (**4d**)
Figure S84. ^{1}H NMR spectrum (*δ,* acetone-d6, 600 MHz) of 4′-hydroxymethylflavanone 4-*O*-*β*-D-(4′′-*O*-methyl)-glucopyranoside (**4d**)
Figure S85. ^{1}H NMR spectrum expansion (*δ,* acetone-d6, 600 MHz) of 4′-hydroxymethylflavanone 4-*O*-*β*-D-(4′′-*O*-methyl)-glucopyranoside (**4d**)
Figure S86. ^{1}H NMR spectrum expansion (*δ,* acetone-d6, 600 MHz) of 4′-hydroxymethylflavanone 4-*O*-*β*-D-(4′′-*O*-methyl)-glucopyranoside (**4d**)
Figure S87. ^{13}C NMR spectrum (*δ,* acetone-d6, 151 MHz) of 4′-hydroxymethylflavanone 4-*O*-*β*-D-(4′′-*O*-methyl)-glucopyranoside (**4d**)
Figure S88. ^{13}C NMR spectrum expansion (*δ,* acetone-d6, 151 MHz) of 4′-hydroxymethylflavanone 4-*O*-*β*-D-(4′′-*O*-methyl)-glucopyranoside (**4d**)
Figure S89. ^{13}C NMR spectrum expansion (*δ,* acetone-d6, 151 MHz) of 4′-hydroxymethylflavanone 4-*O*-*β*-D-(4′′-*O*-methyl)-glucopyranoside (**4d**)
Figure S90. COSY contour map – ^{1}H x ^{1}H of 4′-hydroxymethylflavanone 4-*O*-*β*-D-(4′′-*O*-methyl)-glucopyranoside (**4d**)
Figure S91. COSY contour map – ^{1}H x ^{1}H expansion of 4′-hydroxymethylflavanone 4-*O*-*β*-D-(4′′-*O*-methyl)-glucopyranoside (**4d**)

Figure S92. COSY contour map – ^{1}H x ^{1}H expansion of 4′-hydroxymethylflavanone 4-*O*-*β*-D-(4′′-*O*-methyl)-glucopyranoside (**4d**)
Figure S93. HSQC contour map – ^{1}H x ^{13}C of 4′-hydroxymethylflavanone 4-*O*-*β*-D-(4′′-*O*-methyl)-glucopyranoside (**4d**)
Figure S94. HSQC contour map – ^{1}H x ^{13}C expansion of 4′-hydroxymethylflavanone 4-*O*-*β*-D-(4′′-*O*-methyl)-glucopyranoside (**4d**)
Figure S95. HSQC contour map – ^{1}H x ^{13}C expansion of 4′-hydroxymethylflavanone 4-*O*-*β*-D-(4′′-*O*-methyl)-glucopyranoside (**4d**)
Figure S96. HMBC contour map – ^{1}H x ^{13}C of 4′-hydroxymethylflavanone 4-*O*-*β*-D-(4′′-*O*-methyl)-glucopyranoside (**4d**)
Figure S97. HMBC contour map – ^{1}H x ^{13}C expansion of 4′-hydroxymethylflavanone 4-*O*-*β*-D-(4′′-*O*-methyl)-glucopyranoside (**4d**)
Figure S98. HMBC contour map – ^{1}H x ^{13}C expansion of 4′-hydroxymethylflavanone 4-*O*-*β*-D-(4′′-*O*-methyl)-glucopyranoside (**4d**)
Figure S99. 4′-Hydroxymethylflavanone 4-*O*-*β*-D-(4′′-*O*-methyl)-glucopyranoside (**4d**) physicochemical and ADME parameters prediction using the SwissADME modelling
Figure S100. 4′-Hydroxymethylflavanone 4-*O*-*β*-D-(4′′-*O*-methyl)-glucopyranoside (**4d**) biological activity prediction using the Way2Drug Pass online modelling
Figure S101. 4′-Hydroxymethylflavanone 4-*O*-*β*-D-(4′′-*O*-methyl)-glucopyranoside (**4d**) antibacterial activity prediction using the Way2Drug AntiBac-Pred modelling
Figure S102. 4′-Hydroxymethylflavanone 4-*O*-*β*-D-(4′′-*O*-methyl)-glucopyranoside (**4d**) antifungal activity prediction using the Way2Drug AntiFun-Pred modelling
Figure S103. 4′-Hydroxymethylflavanone 4-*O*-*β*-D-(4′′-*O*-methyl)-glucopyranoside (**4d**) antiviral activity prediction using the Way2Drug AntiVir-Pred modelling

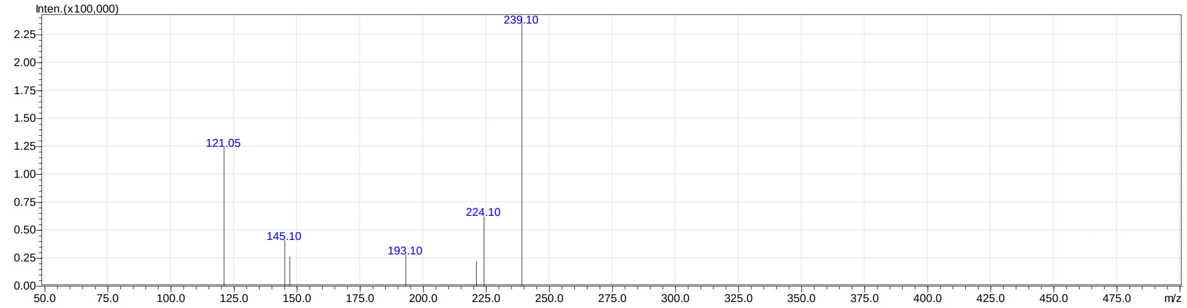

Figure S1. MS analysis of 4′-methylflavanone (**4**)

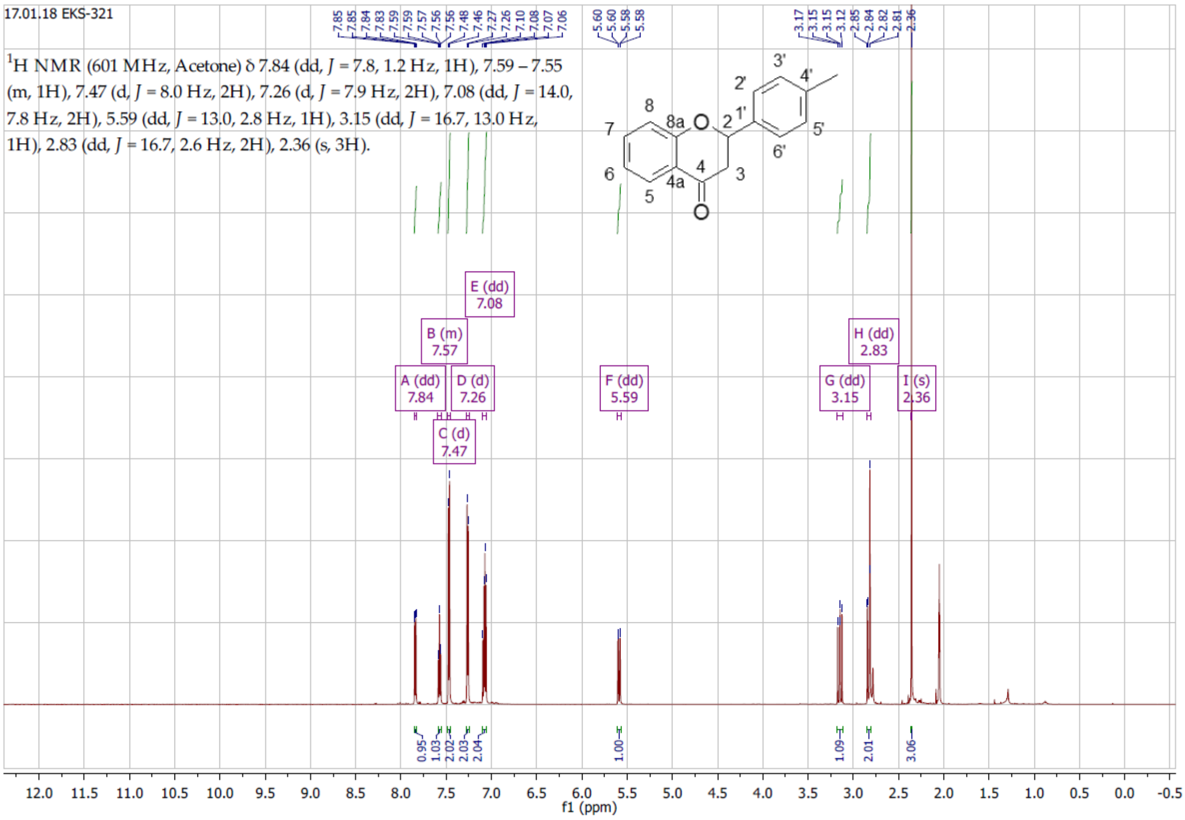

Figure S2. ^{1}H NMR spectrum (*δ*, acetone-d6, 600 MHz) of 4′-methylflavanone (**4**)

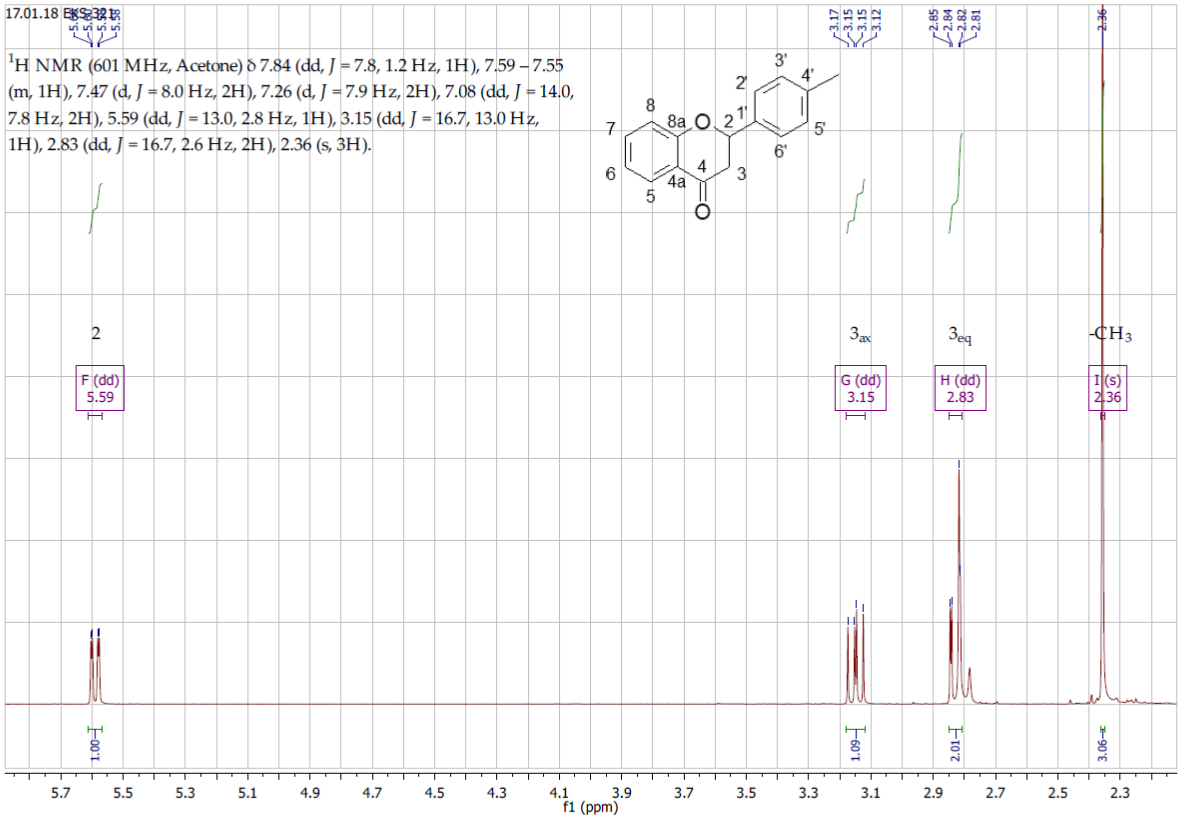

Figure S3. ^{1}H NMR spectrum expansion (*δ*, acetone-d6, 600 MHz) of 4′-methylflavanone (**4**)

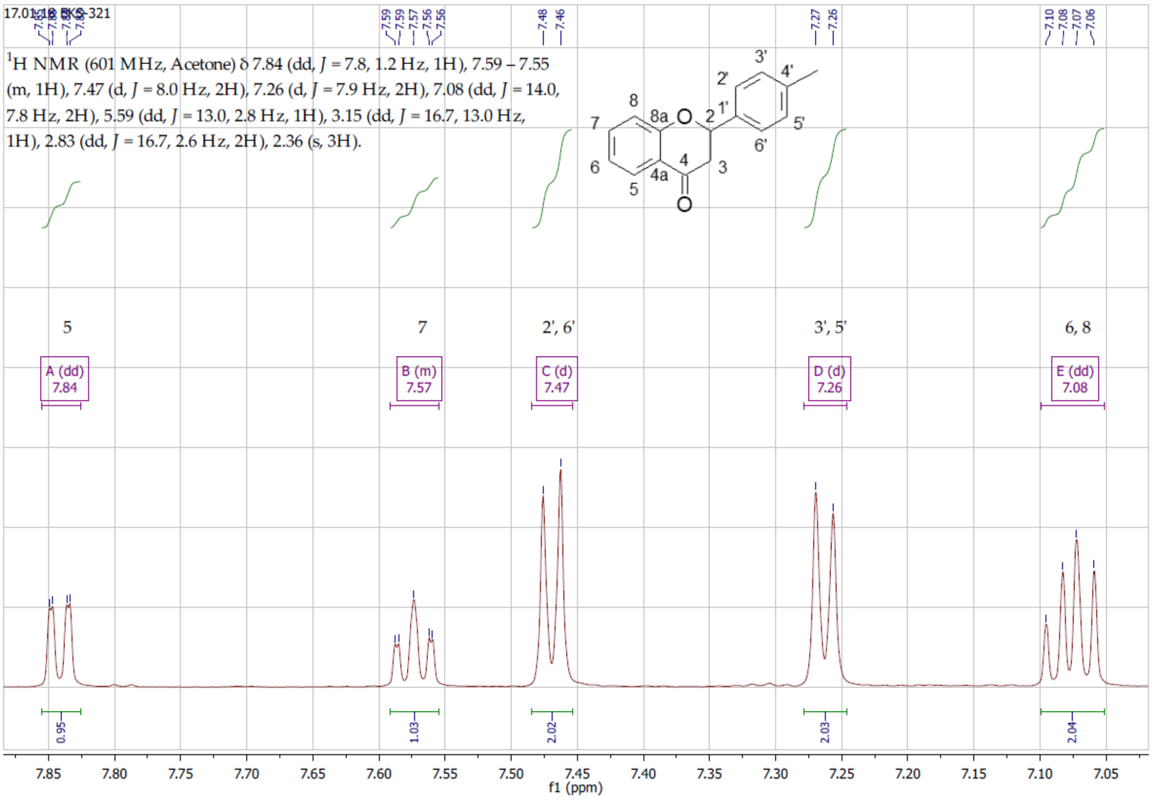

Figure S4. ^{1}H NMR spectrum expansion (*δ*, acetone-d6, 600 MHz) of 4′-methylflavanone (**4**)

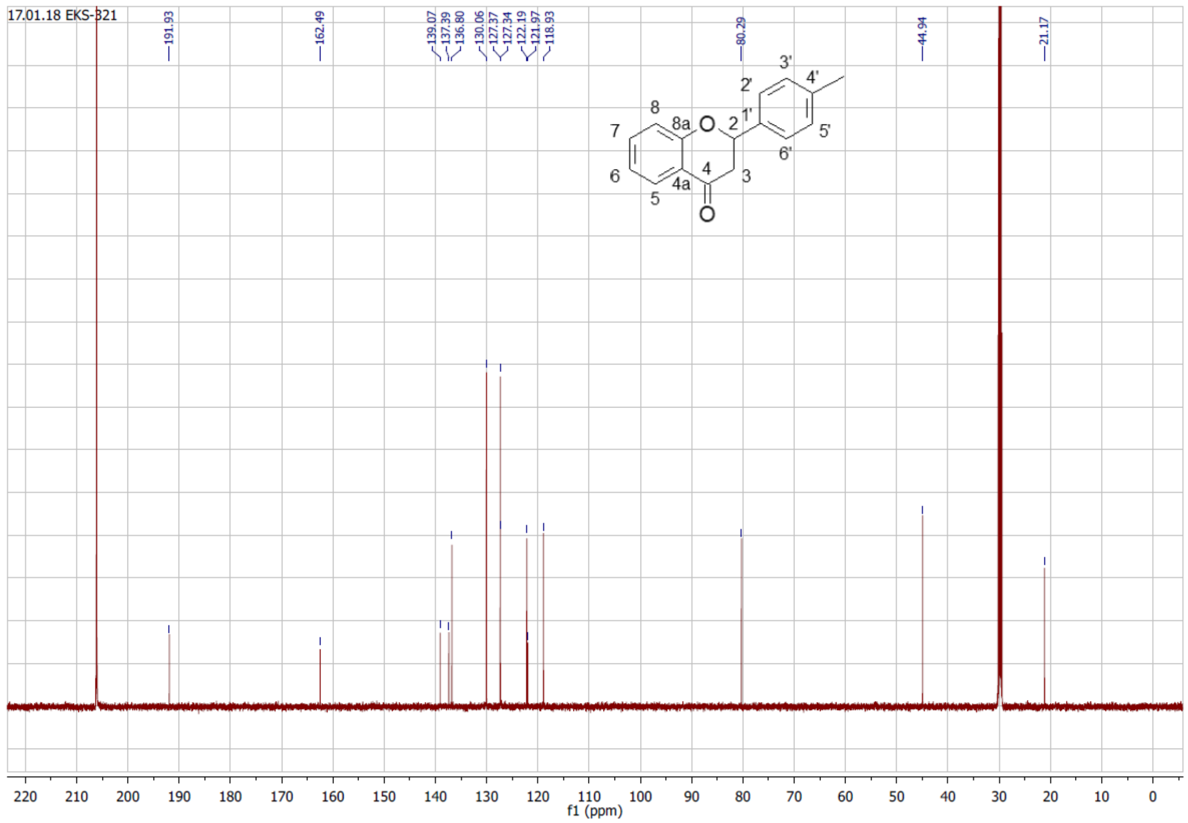

Figure S5. ^{13}C NMR spectrum (δ, acetone-d6, 151 MHz) of 4′-methylflavanone (**4**)

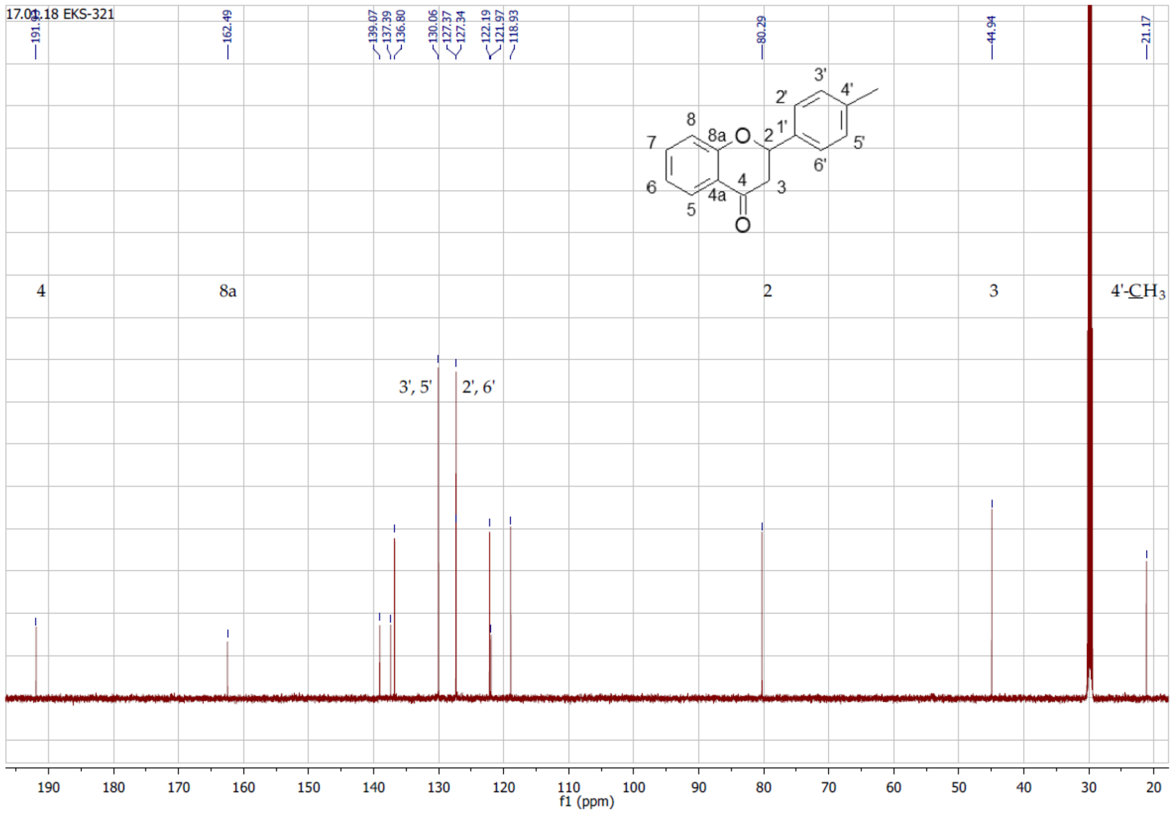

Figure S6. ^{13}C NMR spectrum expansion (δ, acetone-d6, 151 MHz) of 4′-methylflavanone (**4**)

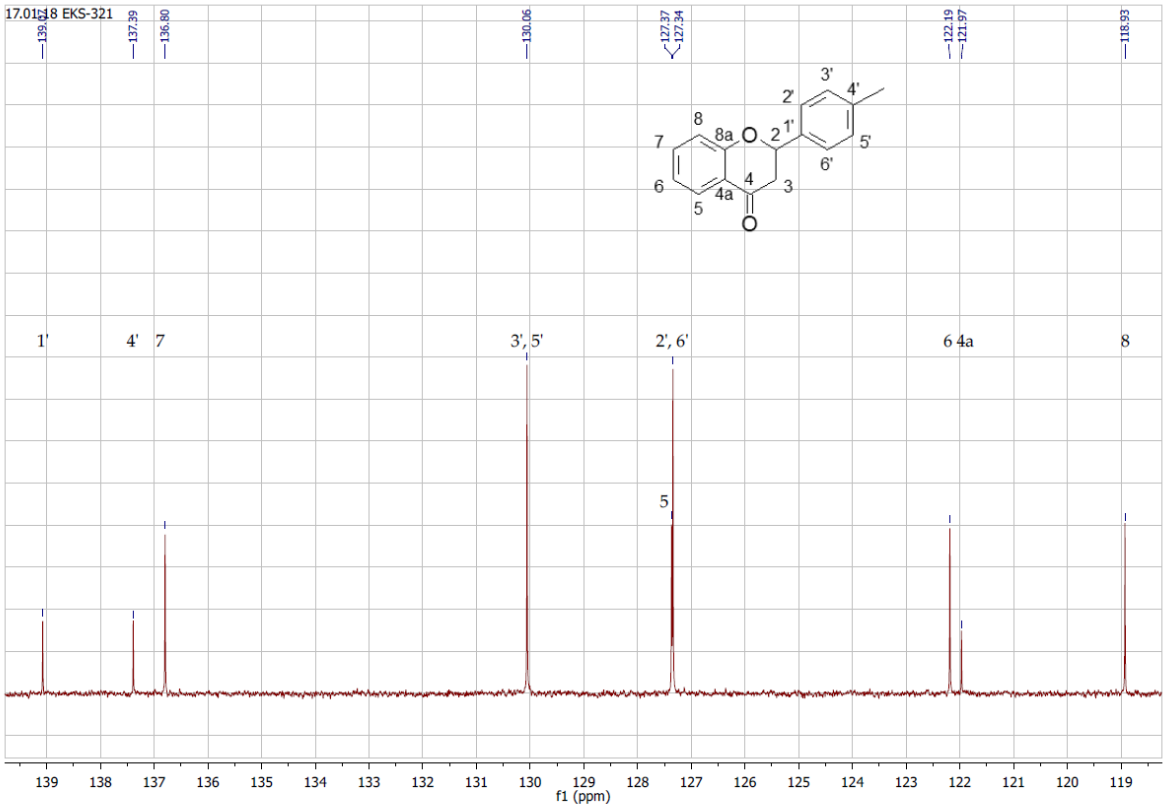

Figure S7. ^{13}C NMR spectrum expansion (δ, acetone-d6, 151 MHz) of 4′-methylflavanone (**4**)

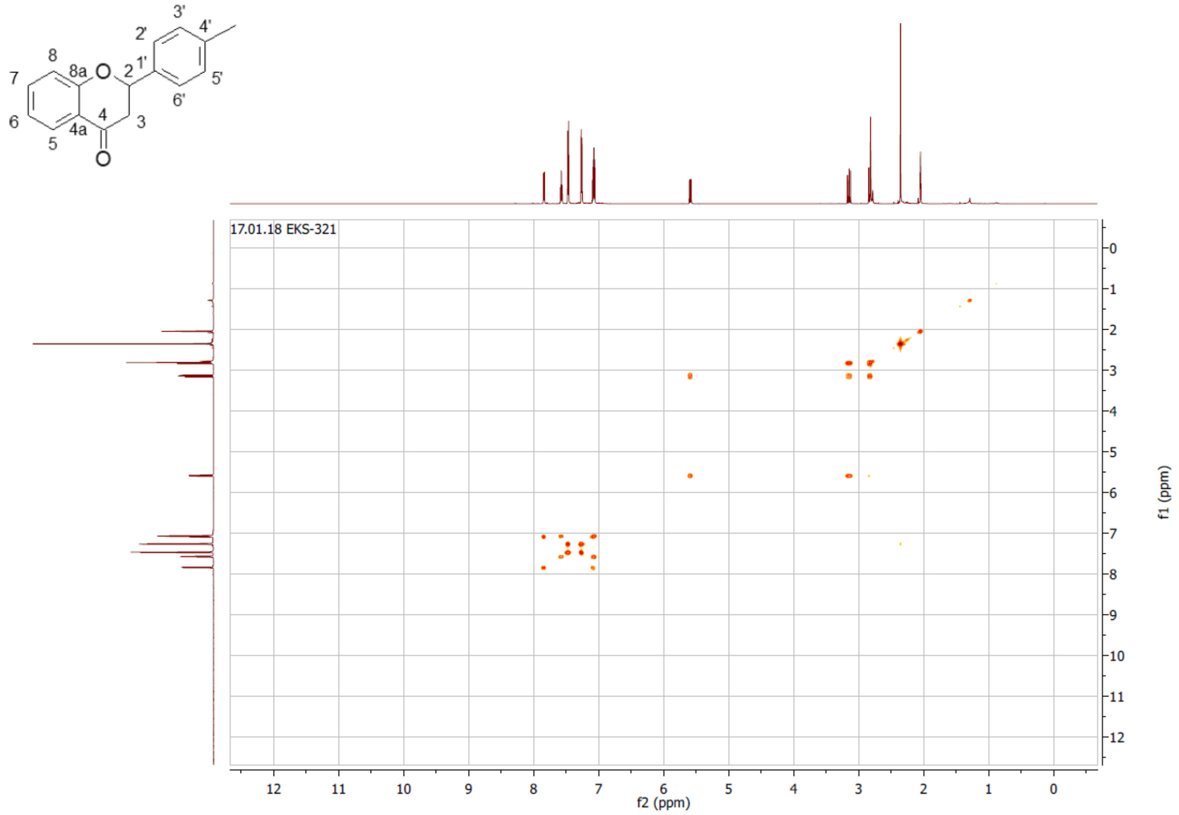

Figure S8. COSY contour map – ^{1}H x ^{1}H of 4′-methylflavanone (**4**)

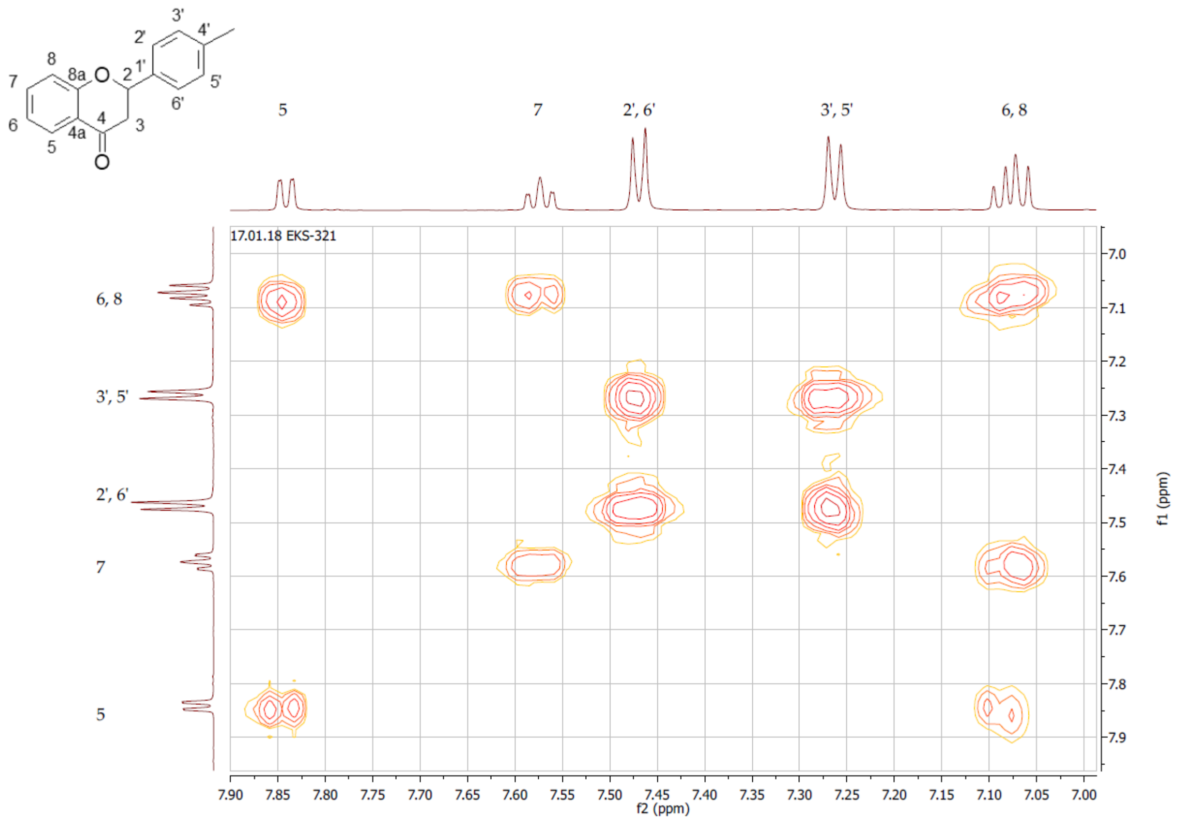

Figure S9. COSY contour map – ^1H x ^1H expansion of 4′-methylflavanone (**4**)

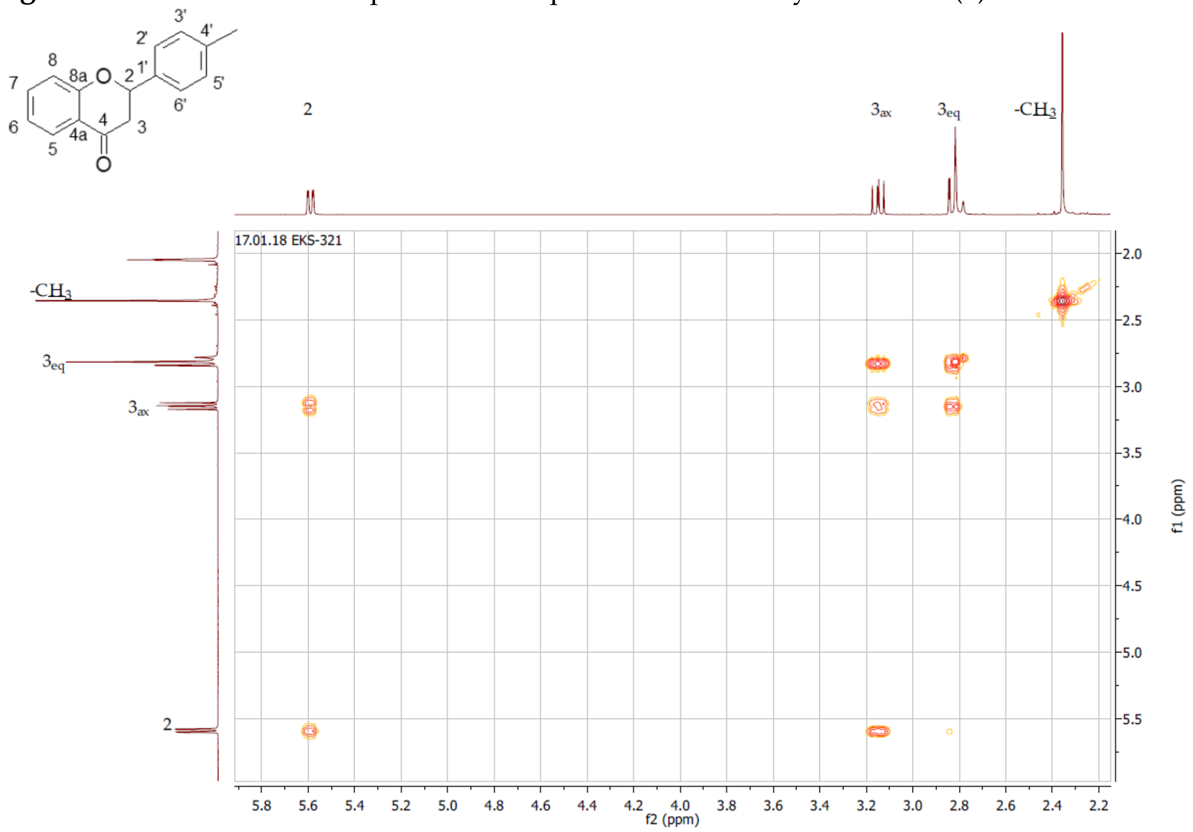

Figure S10. COSY contour map – ^1H x ^1H expansion of 4′-methylflavanone (**4**)

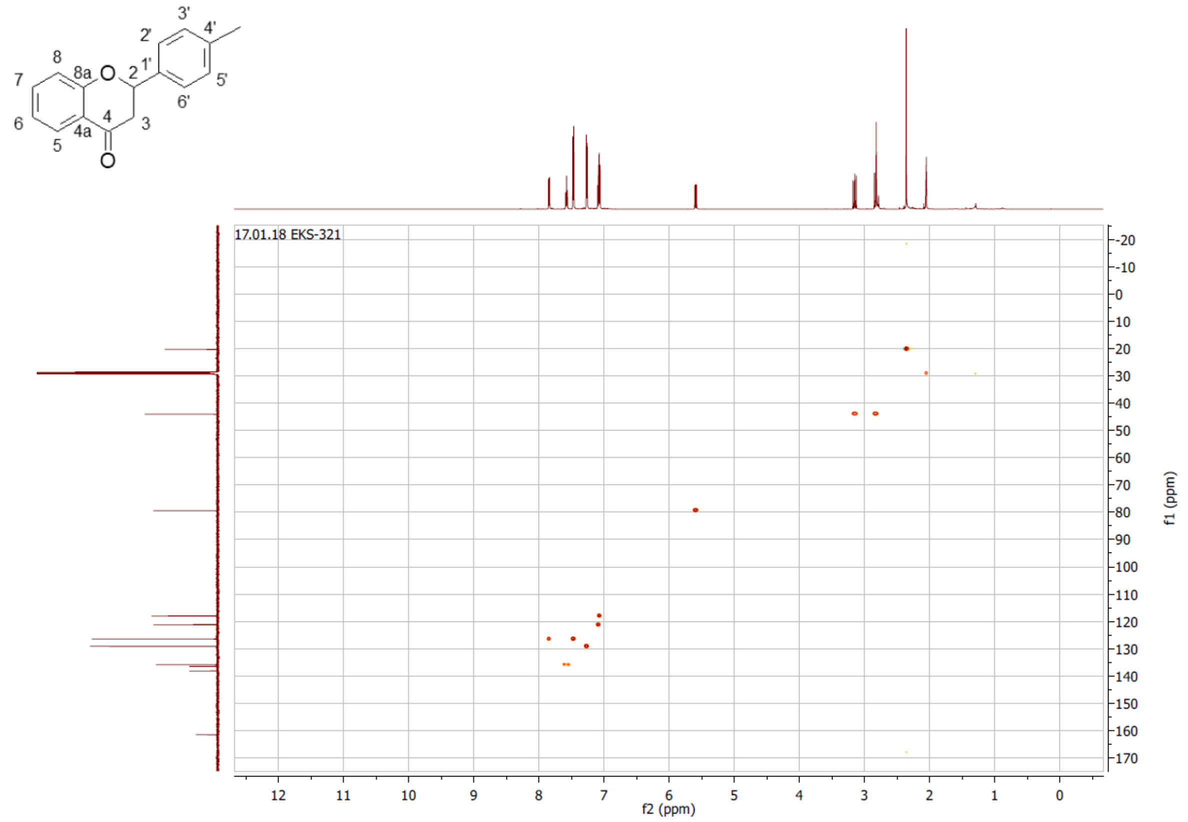

Figure S11. HSQC contour map – ^{1}H x ^{13}C of 4′-methylflavanone (**4**)

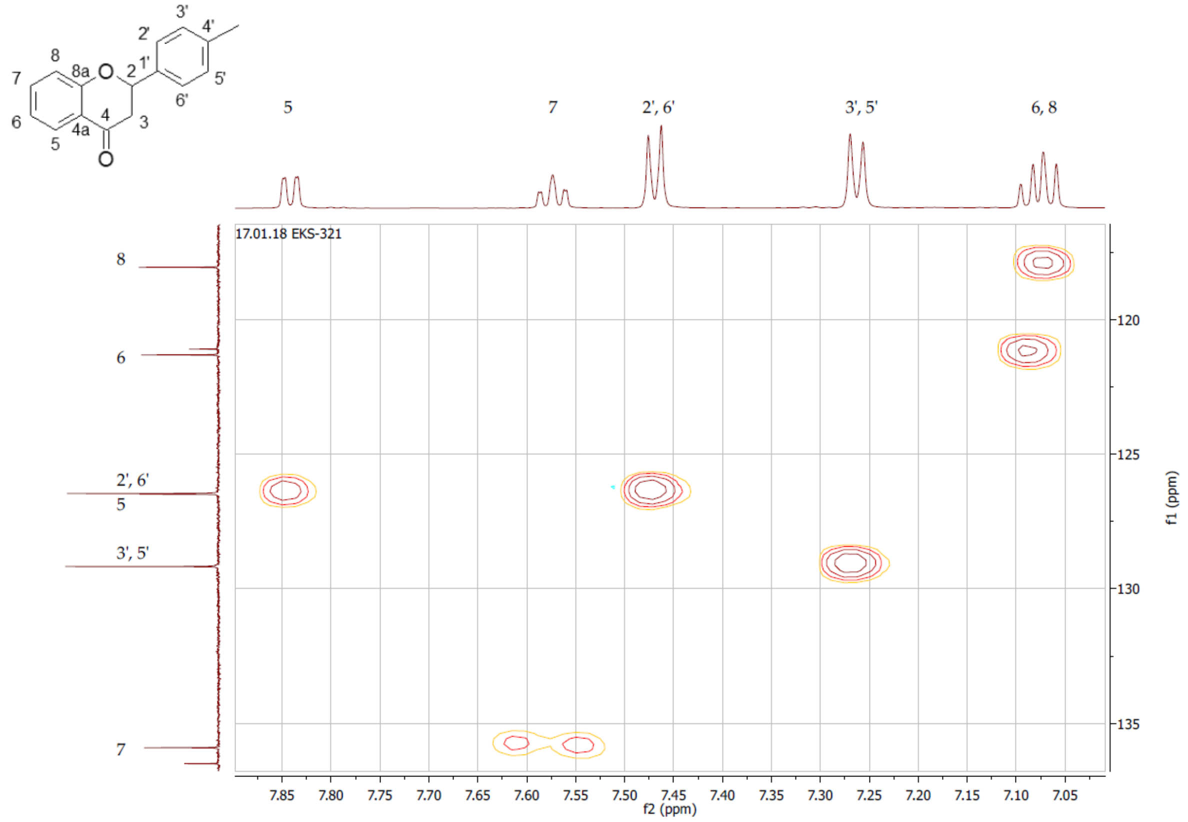

Figure S12. HSQC contour map – ^{1}H x ^{13}C expansion of 4′-methylflavanone (**4**)

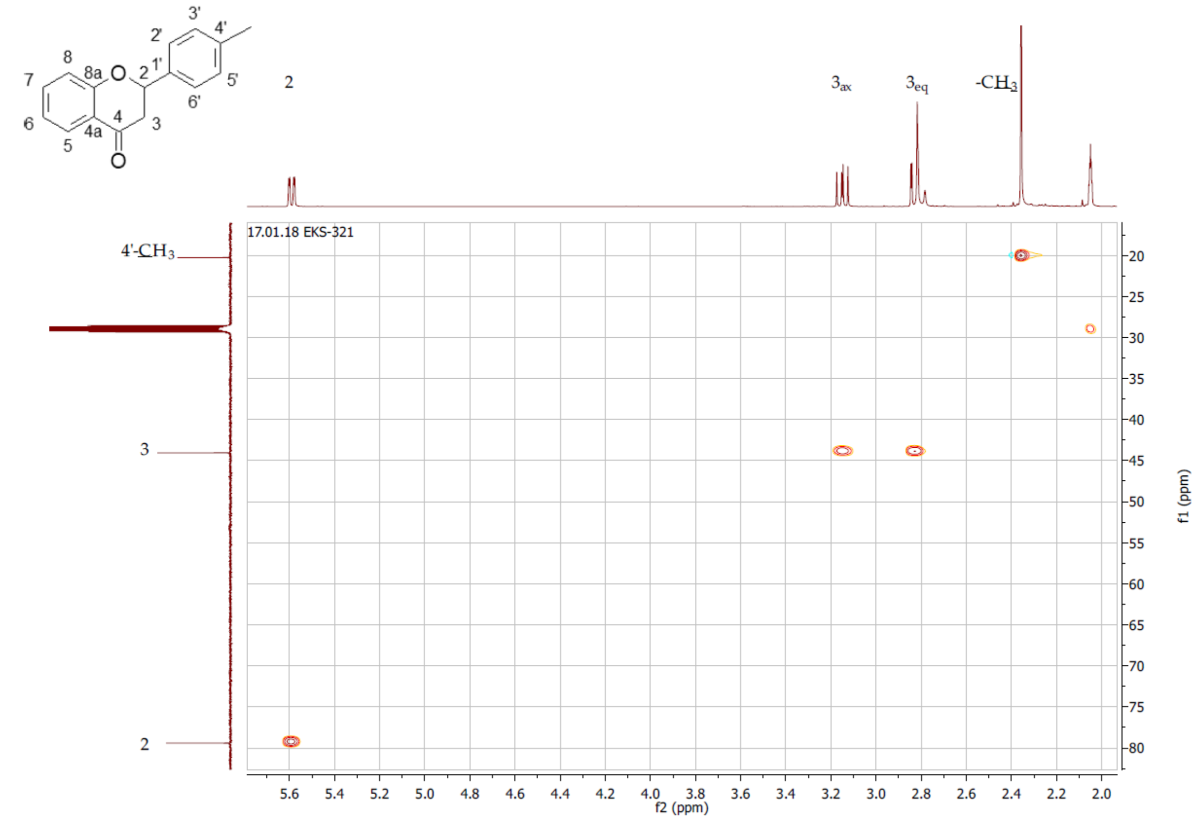

Figure S13. HSQC contour map – ^{1}H x ^{13}C expansion of 4′-methylflavanone (**4**)

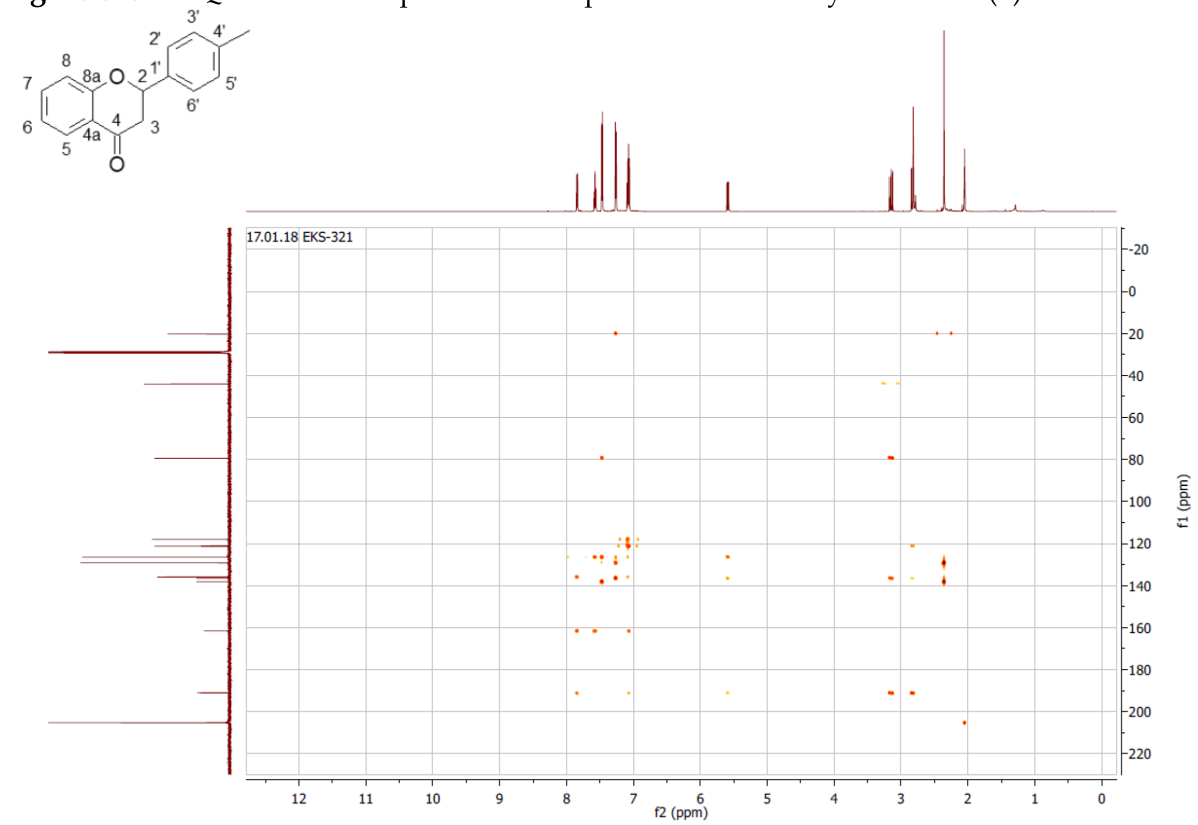

Figure S14. HMBC contour map – ^{1}H x ^{13}C of 4′-methylflavanone (**4**)

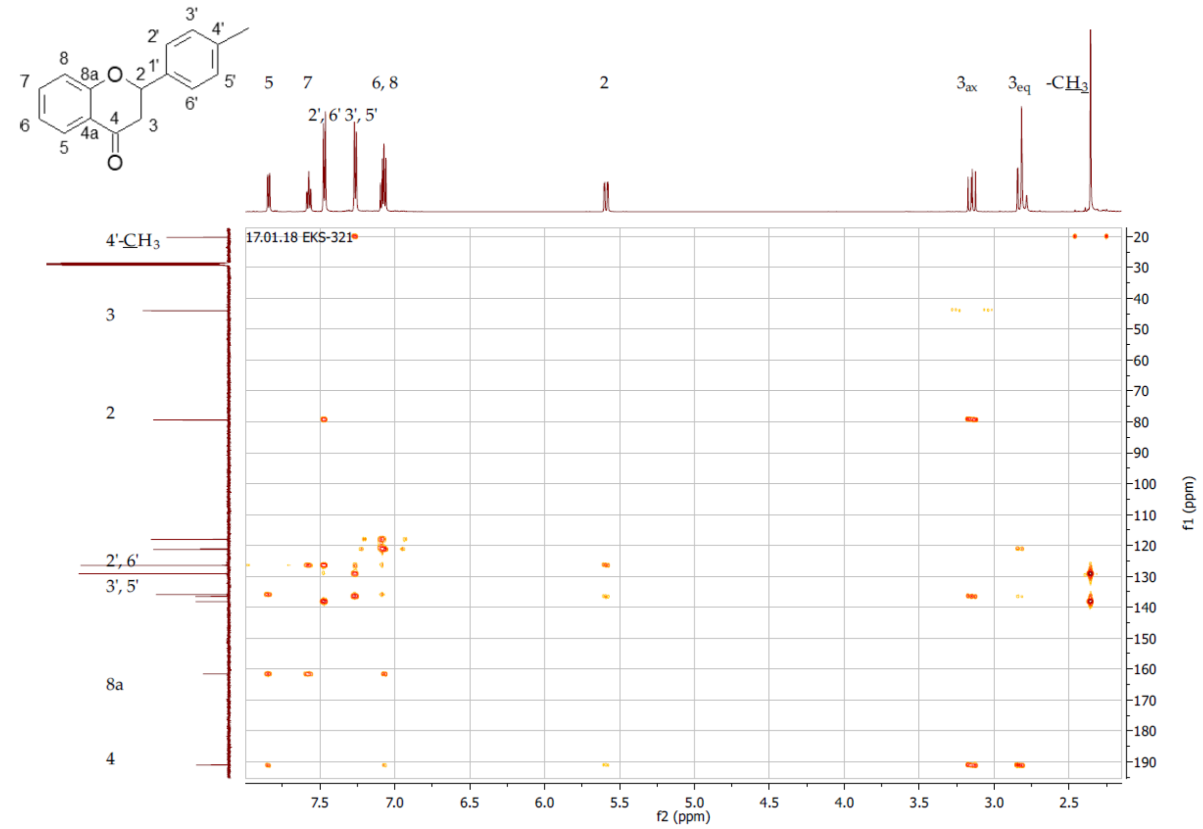

Figure S15. HMBC contour map – ^{1}H x ^{13}C expansion of 4′-methylflavanone (**4**)

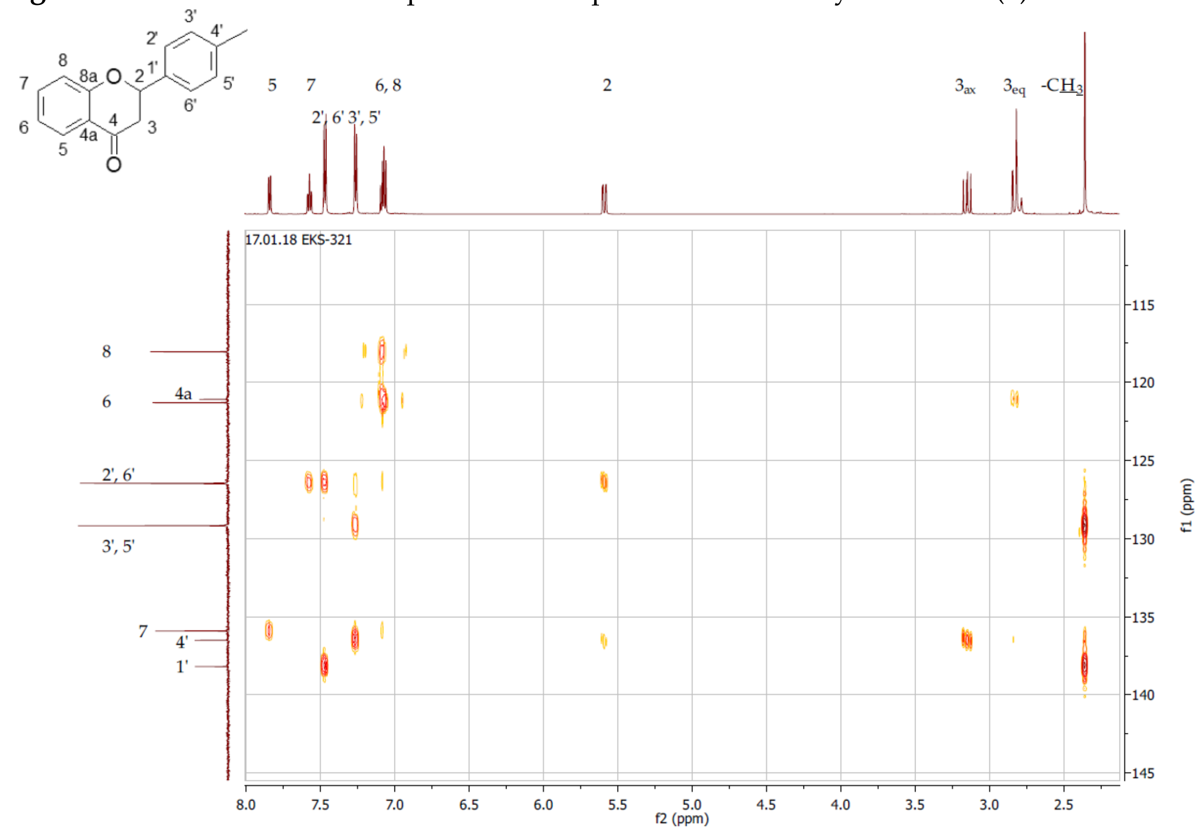

Figure S16. HMBC contour map – ^{1}H x ^{13}C expansion of 4′-methylflavanone (**4**)

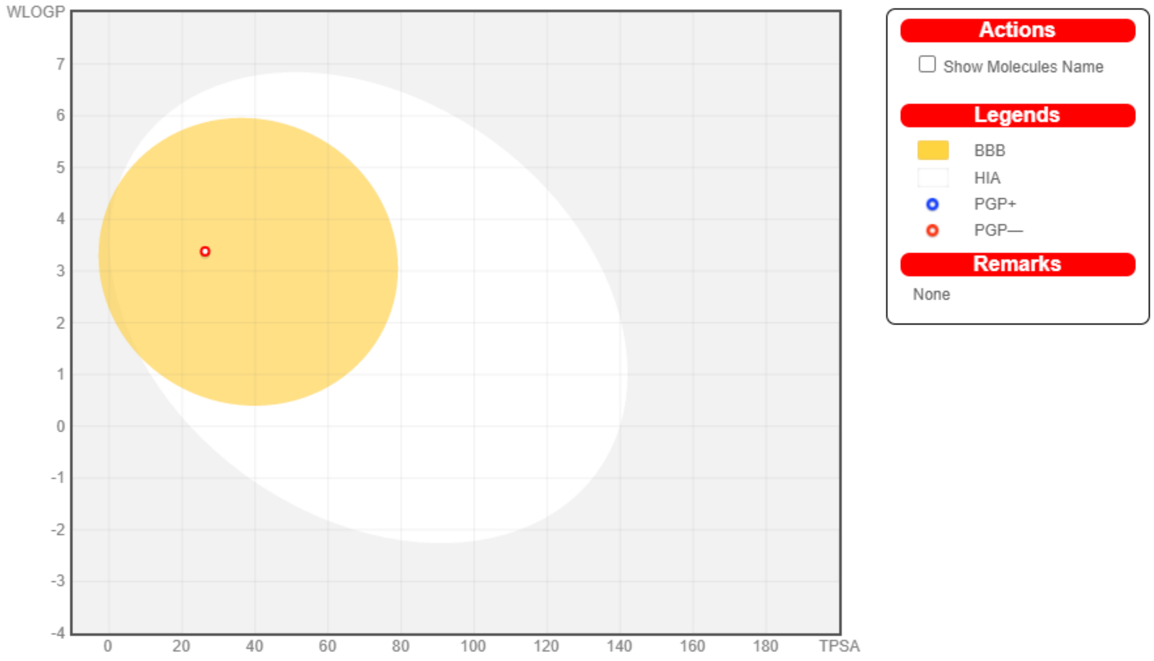

Molecule 1

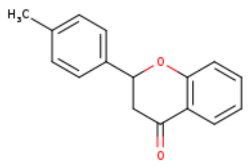

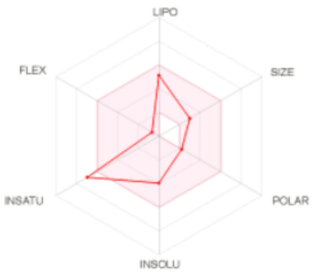

SMILES Cc1ccc(cc1)C1CC(=O)c2c(O1)cccc2

Physicochemical Properties	
Formula	C16H14O2
Molecular weight	238.28 g/mol
Num. heavy atoms	18
Num. arom. heavy atoms	12
Fraction Csp3	0.19
Num. rotatable bonds	1
Num. H-bond acceptors	2
Num. H-bond donors	0
Molar Refractivity	70.47
TPSA	26.30 Å²

Lipophilicity	
Log $P_{o/w}$ (iLOGP)	2.70
Log $P_{o/w}$ (XLOGP3)	3.50
Log $P_{o/w}$ (WLOGP)	3.38
Log $P_{o/w}$ (MLOGP)	2.71
Log $P_{o/w}$ (SILICOS-IT)	4.05
Consensus Log $P_{o/w}$	3.27

Water Solubility	
Log *S* (ESOL)	-3.95
Solubility	2.68e-02 mg/ml ; 1.12e-04 mol/l
Class	Soluble
Log *S* (Ali)	-3.74
Solubility	4.38e-02 mg/ml ; 1.84e-04 mol/l
Class	Soluble
Log *S* (SILICOS-IT)	-5.54
Solubility	6.92e-04 mg/ml ; 2.90e-06 mol/l
Class	Moderately soluble

Pharmacokinetics	
GI absorption	High
BBB permeant	Yes
P-gp substrate	No
CYP1A2 inhibitor	Yes
CYP2C19 inhibitor	Yes
CYP2C9 inhibitor	No
CYP2D6 inhibitor	Yes
CYP3A4 inhibitor	No
Log K_p (skin permeation)	-5.27 cm/s

Druglikeness	
Lipinski	Yes; 0 violation
Ghose	Yes
Veber	Yes
Egan	Yes
Muegge	Yes
Bioavailability Score	0.55

Medicinal Chemistry	
PAINS	0 alert
Brenk	0 alert
Leadlikeness	No; 1 violation: MW<250
Synthetic accessibility	2.88

Figure S17. 4′-Methylflavanone (**4**) physicochemical and ADME parameters prediction using the SwissADME modelling

Pa	Pi	Activity
0,927	0,005	Membrane integrity agonist
0,896	0,002	Cytochrome P450 stimulant
0,885	0,005	CYP1A substrate
0,863	0,004	CYP1A1 substrate
0,843	0,005	CYP2B6 substrate
0,842	0,010	Mucomembranous protector
0,834	0,003	HMOX1 expression enhancer
0,824	0,005	CYP1A2 substrate
0,807	0,035	CYP2C12 substrate
0,771	0,008	CYP2B substrate

Figure S18. 4′-Methylflavanone (**4**) biological activity prediction using the Way2Drug Pass online modelling

Name	Confidence	ChEMBL ID
Listeria monocytogenes	0.4989	CHEMBL614974
Yersinia pestis	0.3724	CHEMBL614597
Bacillus subtilis	0.2393	CHEMBL359
Pseudomonas fluorescens	0.2343	CHEMBL612500
Bacillus subtilis subsp. subtilis str. 168	0.2137	CHEMBL613315
Mycobacterium	0.1674	CHEMBL614981
RESISTANT Burkholderia pseudomallei	0.1401	CHEMBL3140323
Mycobacterium aurum	0.1399	CHEMBL612952
RESISTANT Staphylococcus aureus subsp. aureus MW2	0.0902	CHEMBL612531
Salmonella enterica subsp. enterica	0.0894	CHEMBL613044
Neisseria gonorrhoeae	0.0879	CHEMBL614430

Figure S19. 4′-Methylflavanone (**4**) antibacterial activity prediction using the Way2Drug AntiBac-Pred modelling

Name	Confidence	ChEMBL ID
Candida dubliniensis	0.7087	CHEMBL613334
Epidermophyton floccosum	0.1832	CHEMBL612386
Trichophyton mentagrophytes	0.0304	CHEMBL613162

Figure S20. 4′-Methylflavanone (**4**) antifungal activity prediction using the Way2Drug AntiFun-Pred modelling

Virus	Protein target	Confidence
Human immunodeficiency virus 2	Human immunodeficiency virus type 2 integrase	0.5891
Severe acute respiratory syndrome coronavirus 2	Replicase polyprotein 1ab	0.3771
Dengue virus type 2	Genome polyprotein	0.2886
Vaccinia virus (strain Western Reserve) (VACV) (Vaccinia virus (strainWR))	DNA polymerase	0.2734
Varicella-zoster virus (strain Dumas) (HHV-3) (Human herpesvirus 3)	DNA polymerase	0.1501
Herpes simplex virus (type 1 / strain 17)	Human herpesvirus 1 DNA polymerase	0.1501

Figure S21. 4′-Methylflavanone (**4**) antiviral activity prediction using the Way2Drug AntiVir-Pred modelling

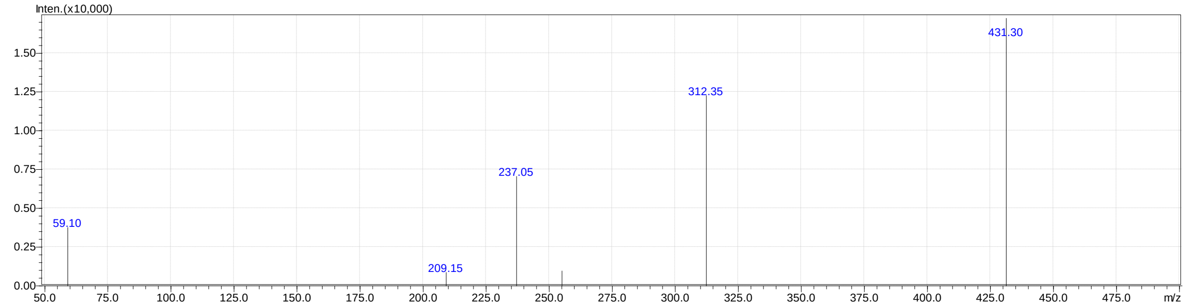

Figure S22. MS analysis of flavanone 4′-methylene-*O*-*β*-D-(4′′-*O*-methyl)-glucopyranoside (**4a**)

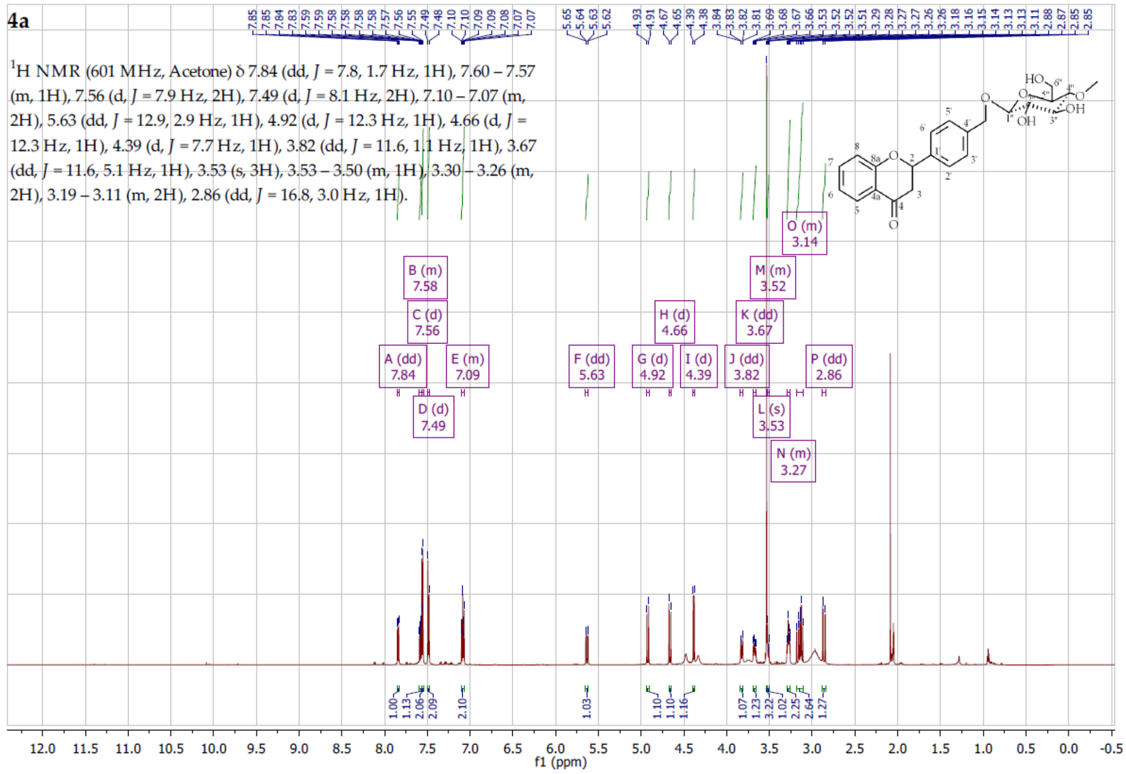

Figure S23. ^{1}H NMR spectrum (*δ*, acetone-d6, 600 MHz) of flavanone 4′-methylene-*O*-*β*-D-(4′′-*O*-methyl)-glucopyranoside (**4a**)

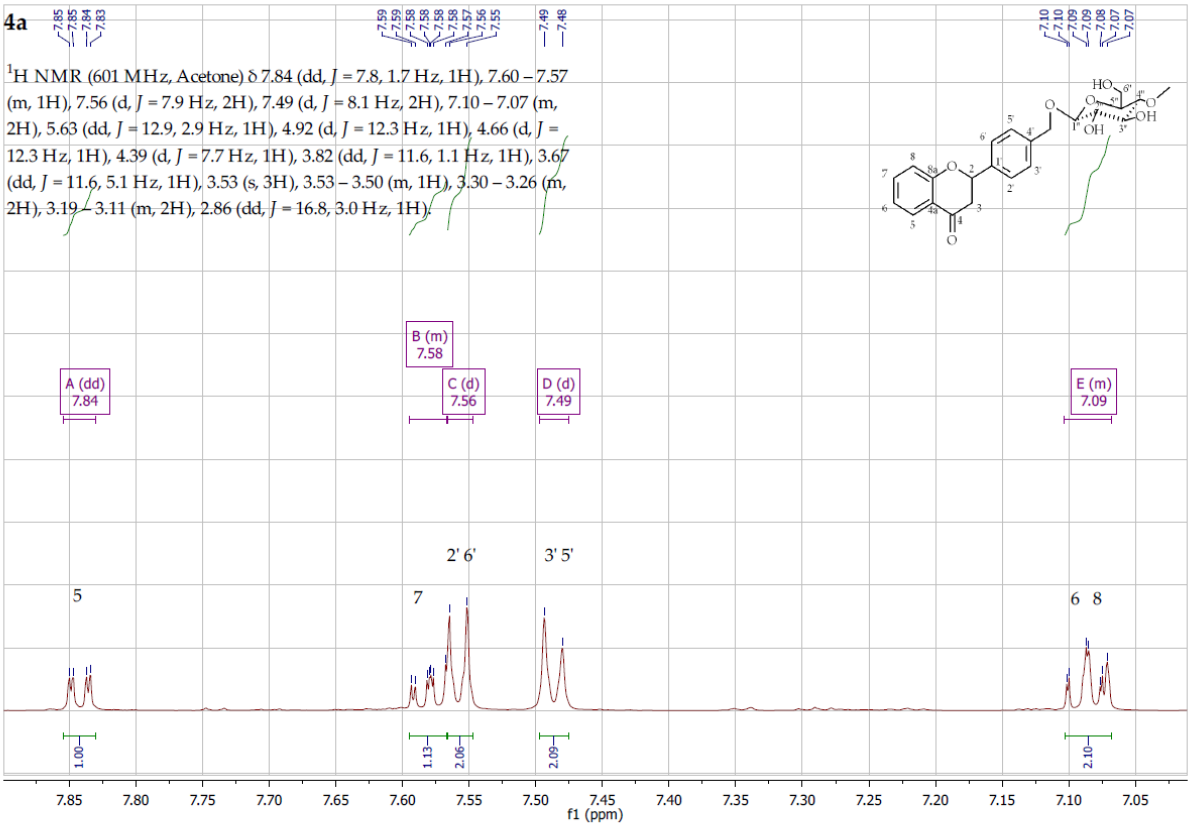

Figure S24. ^{1}H NMR spectrum expansion (*δ*, acetone-d6, 600 MHz) of flavanone 4′-methylene-*O*-*β*-D-(4′′-*O*-methyl)-glucopyranoside (**4a**)

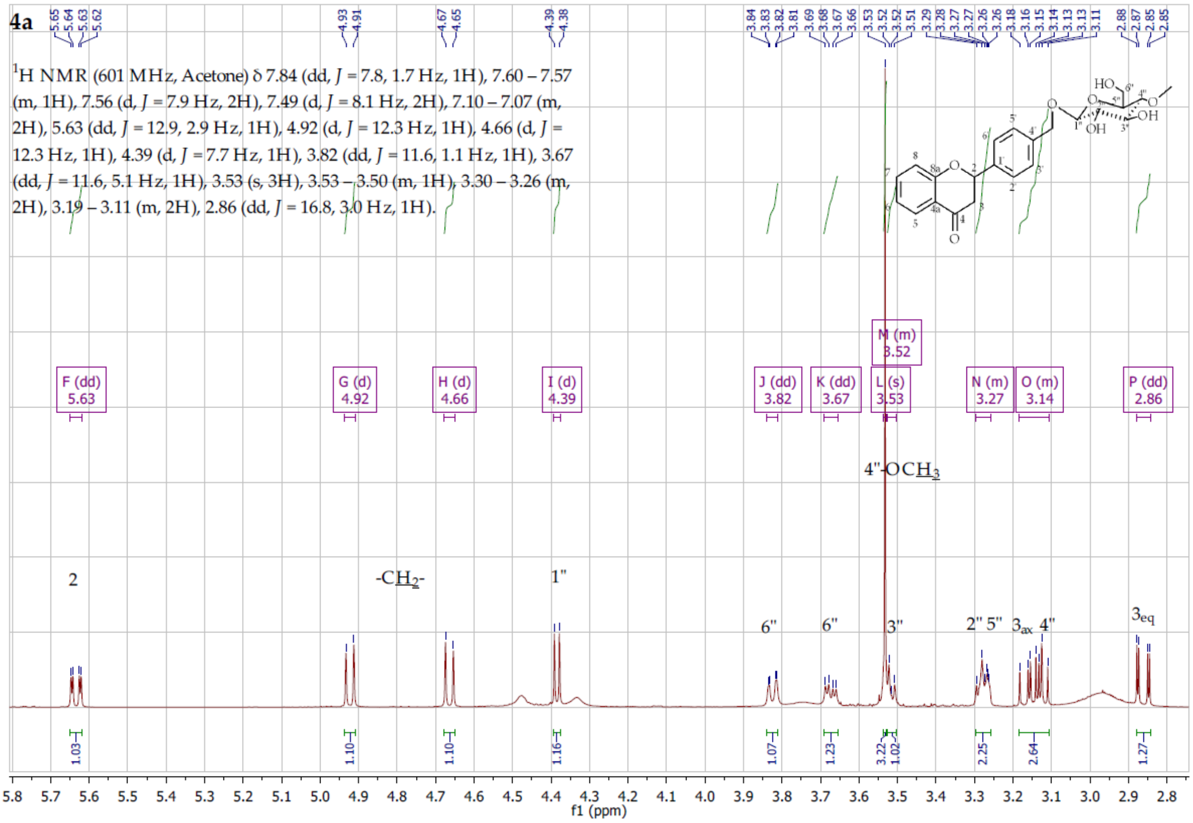

Figure S25. ^{1}H NMR spectrum expansion (*δ*, acetone-d6, 600 MHz) of flavanone 4′-methylene-*O*-*β*-D-(4′′-*O*-methyl)-glucopyranoside (**4a**)

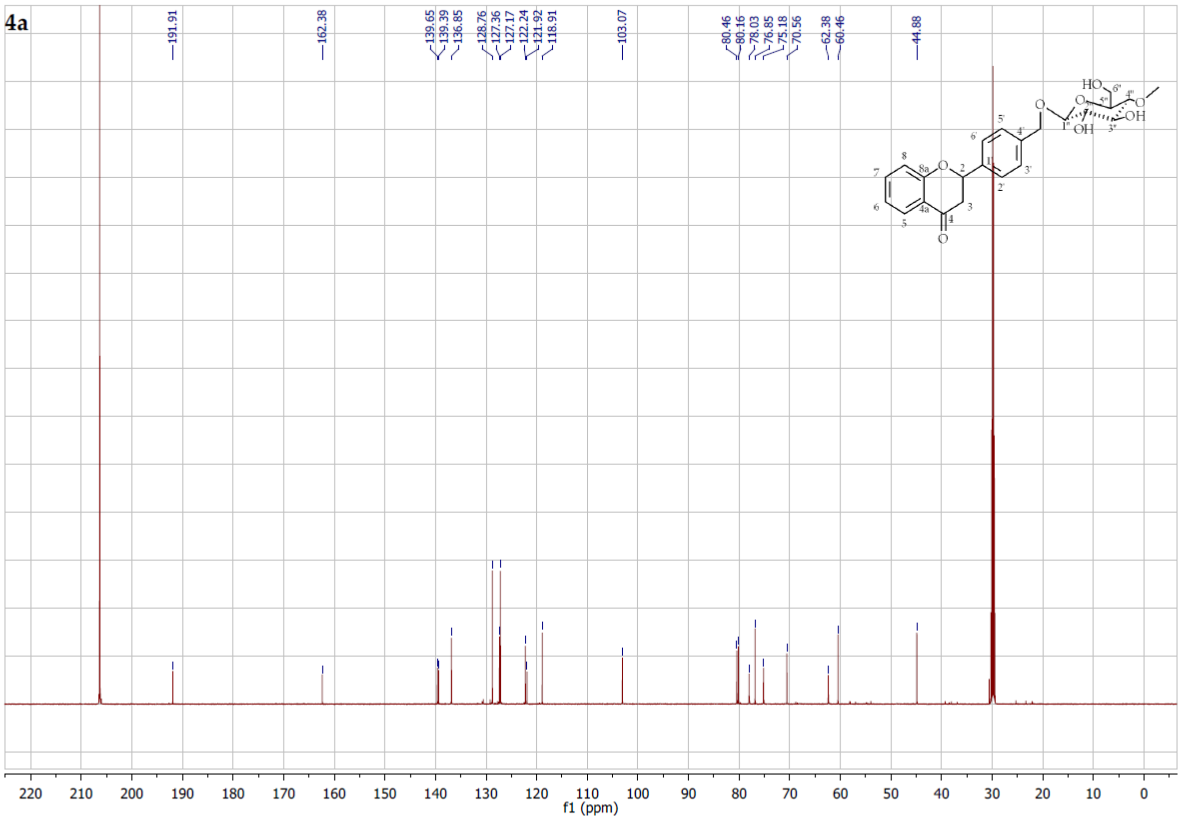

Figure S26. ^{13}C NMR spectrum (*δ*, acetone-d6, 151 MHz) of flavanone 4′-methylene-*O*-*β*-D-(4′′-*O*-methyl)-glucopyranoside (**4a**)

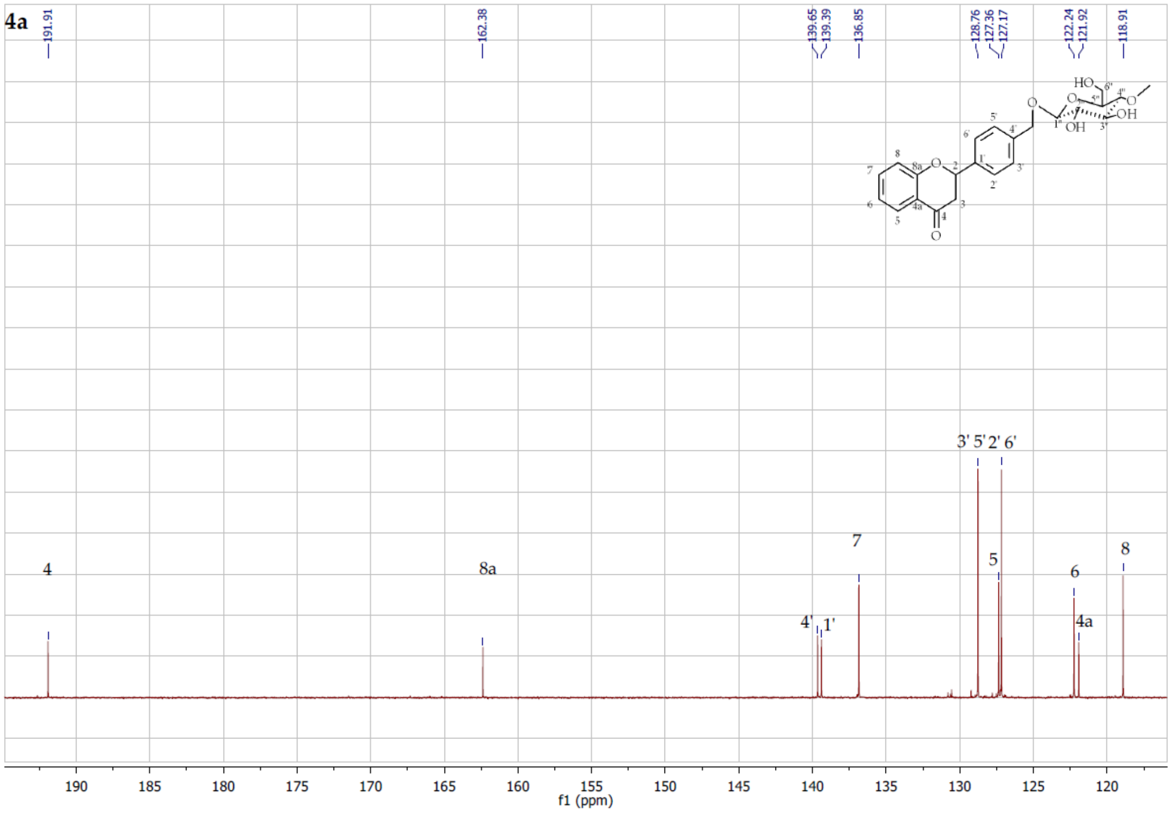

Figure S27. ^{13}C NMR spectrum expansion (*δ*, acetone-d6, 151 MHz) of flavanone 4′-methylene-*O*-*β*-D-(4′′-*O*-methyl)-glucopyranoside (**4a**)

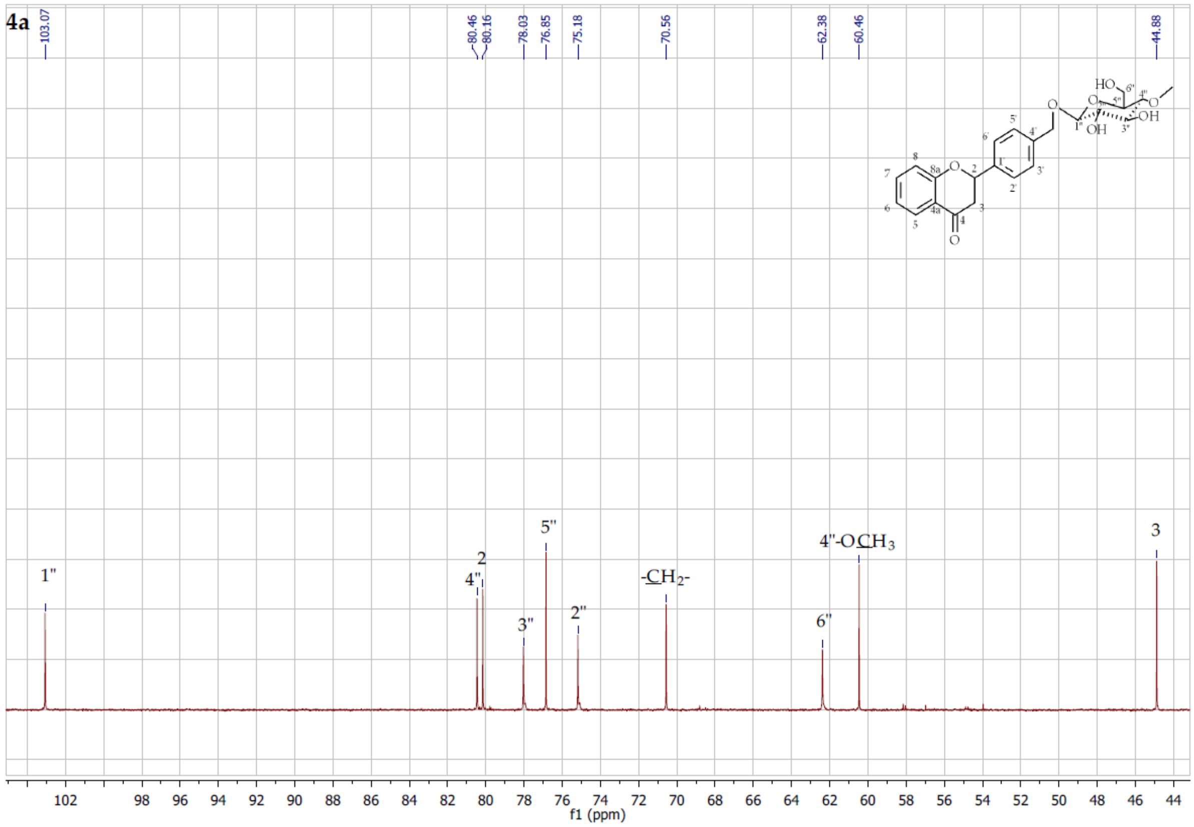

Figure S28. ^{13}C NMR spectrum expansion (*δ*, acetone-d6, 151 MHz) of flavanone 4′-methylene-*O*-*β*-D-(4′′-*O*-methyl)-glucopyranoside (**4a**)

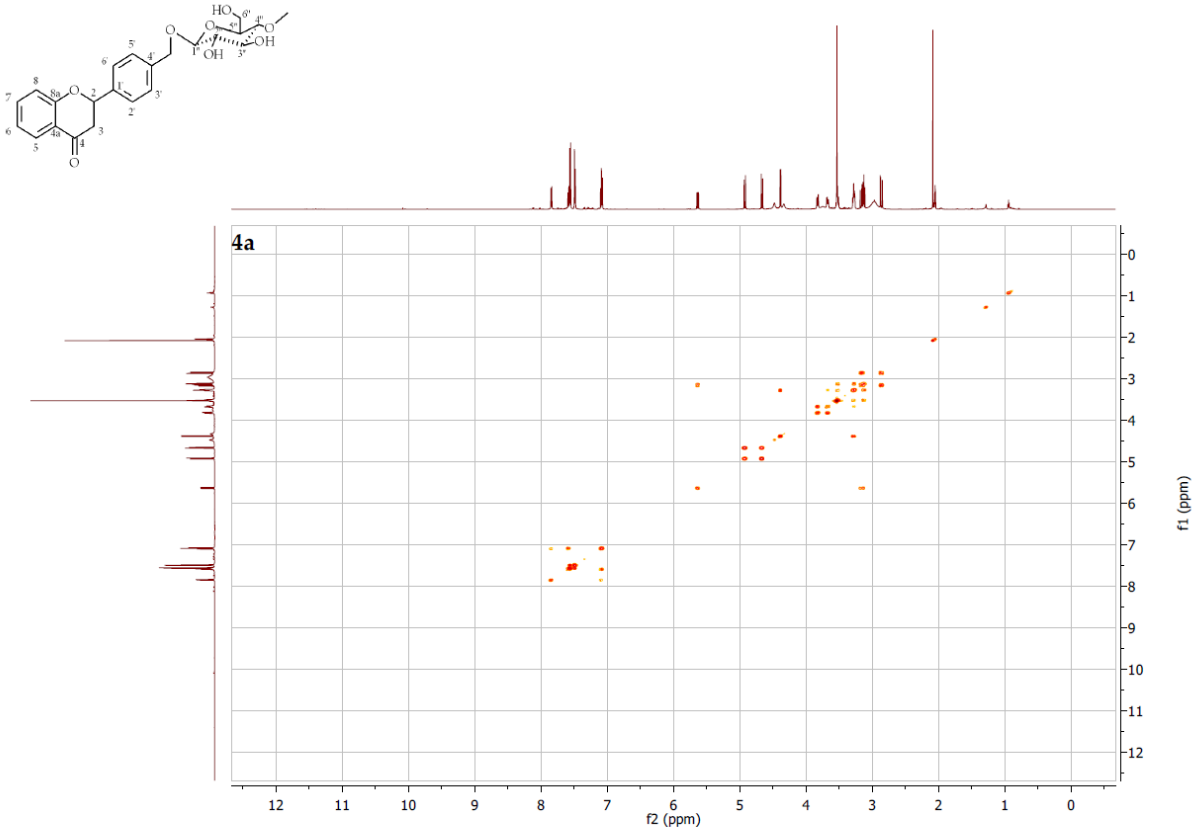

Figure S29. COSY contour map – ^{1}H x ^{1}H of flavanone 4′-methylene-*O*-*β*-D-(4′′-*O*-methyl)-glucopyranoside (**4a**)

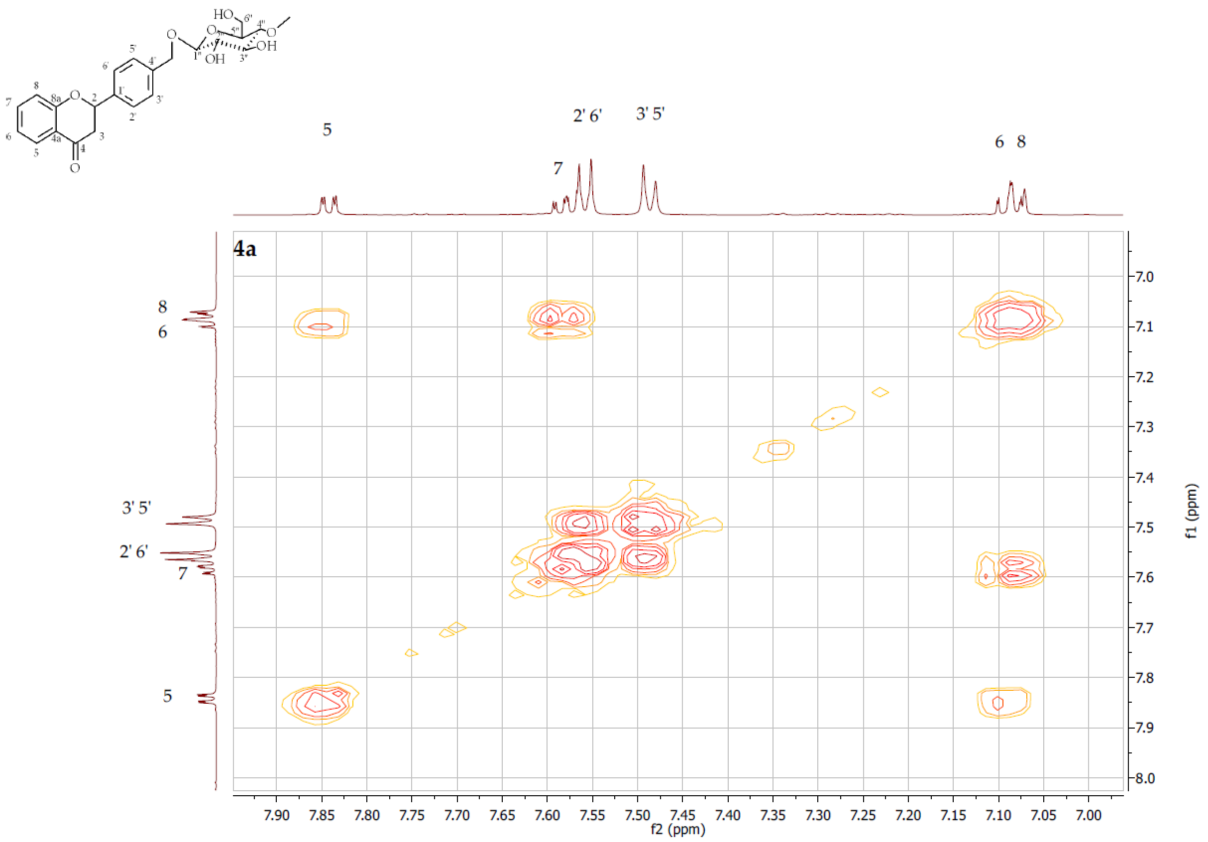

Figure S30. COSY contour map – ^{1}H x ^{1}H expansion of flavanone 4′-methylene-*O*-*β*-D-(4′′-*O*-methyl)-glucopyranoside (**4a**)

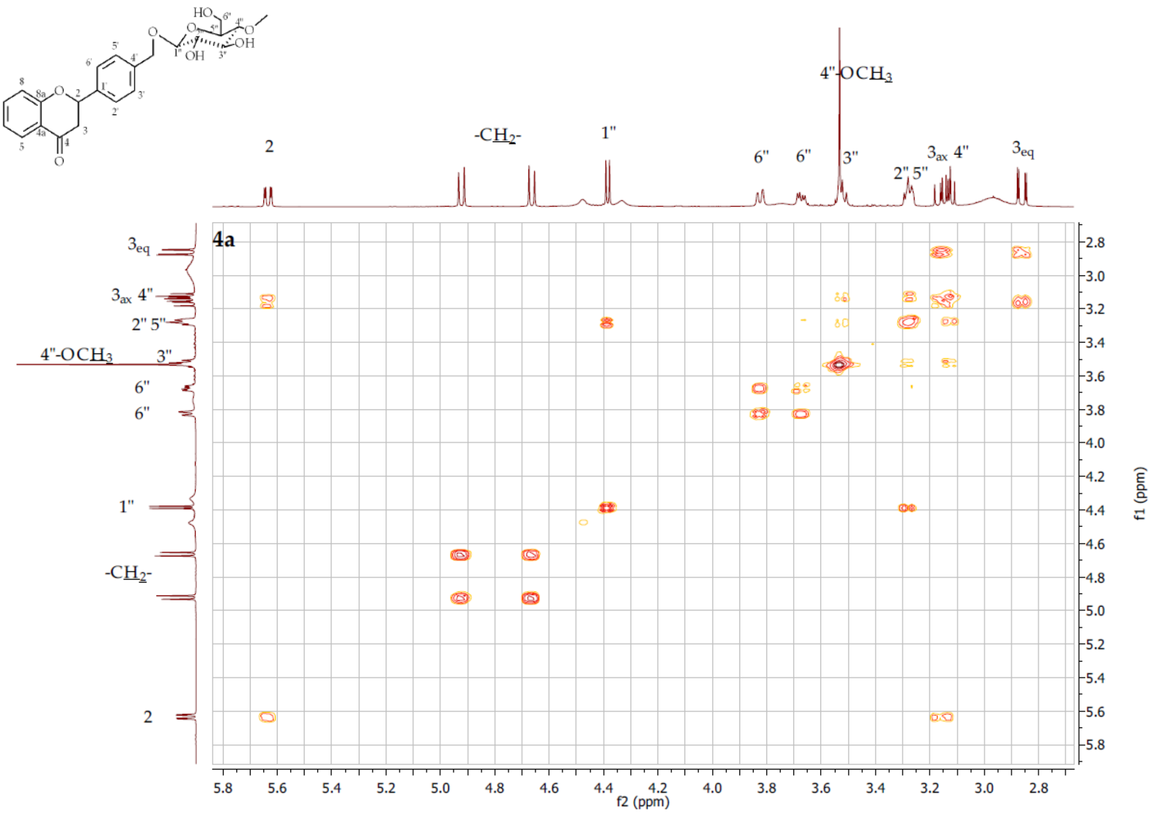

Figure S31. COSY contour map – ^{1}H x ^{1}H expansion of flavanone 4′-methylene-*O*-*β*-D-(4′′-*O*-methyl)-glucopyranoside (**4a**)

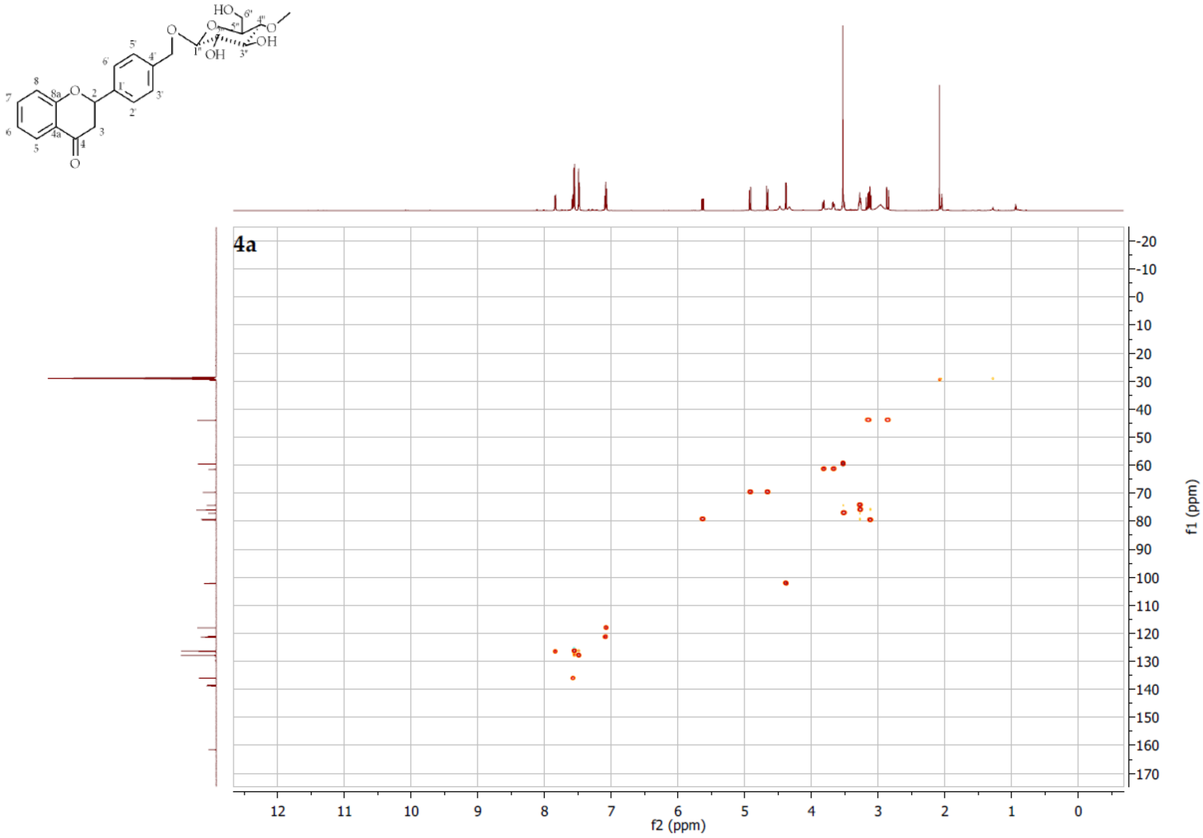

Figure S32. HSQC contour map – ^{1}H x ^{13}C of flavanone 4′-methylene-*O*-*β*-D-(4′′-*O*-methyl)-glucopyranoside (**4a**)

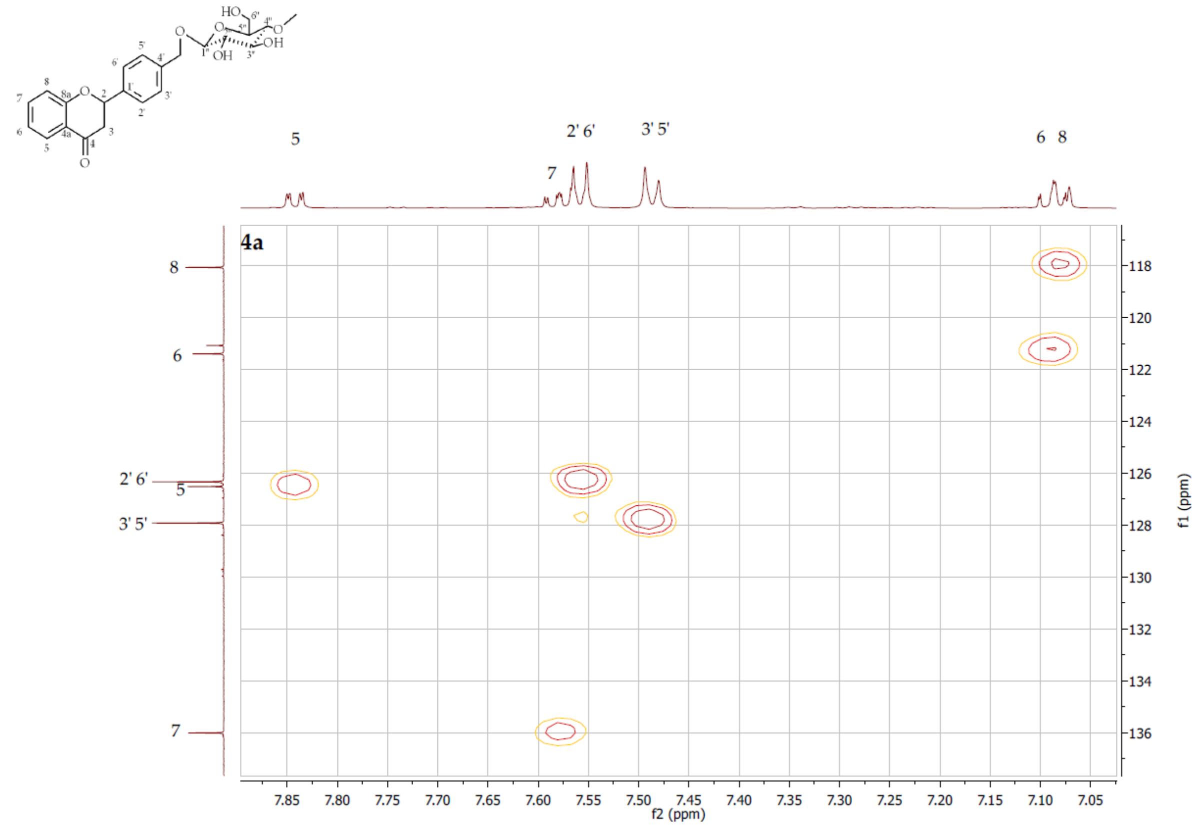

Figure S33. HSQC contour map – ^{1}H x ^{13}C expansion of flavanone 4′-methylene-*O*-*β*-D-(4′′-*O*-methyl)-glucopyranoside (**4a**)

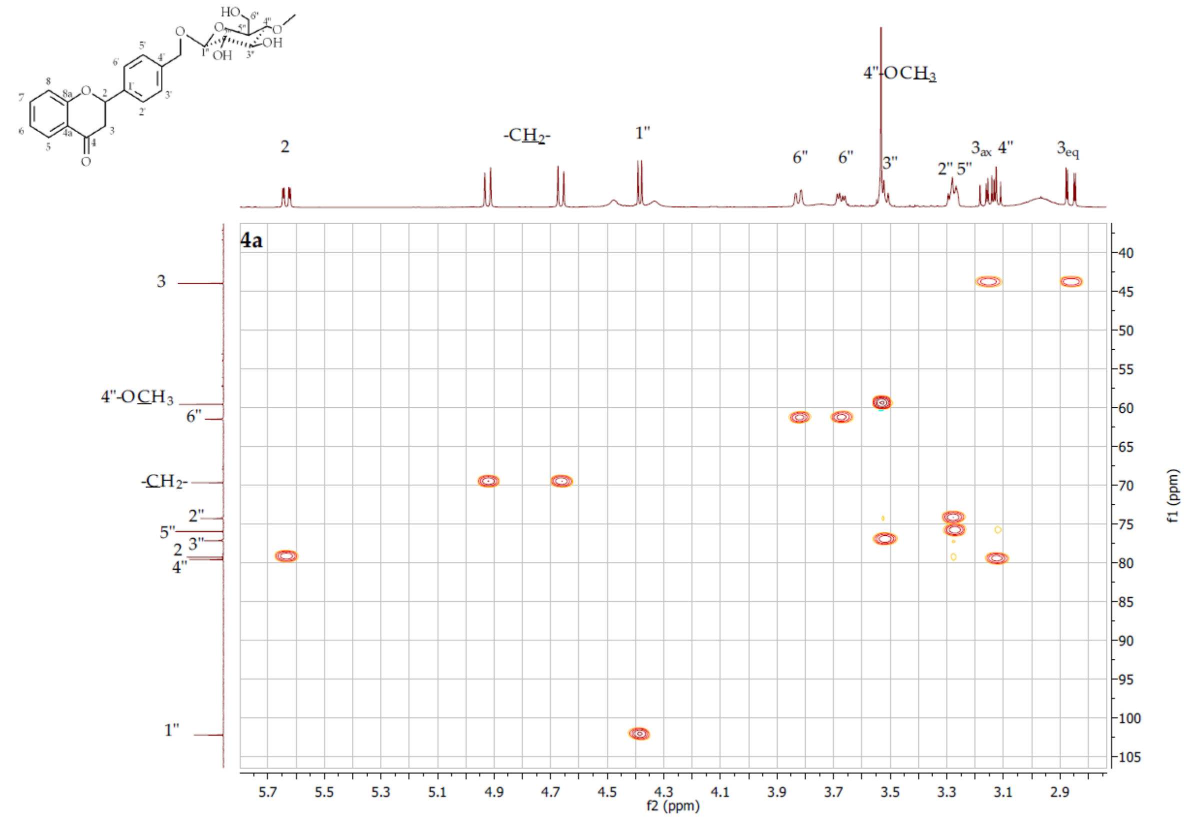

Figure S34. HSQC contour map – $^{1}H \times ^{13}C$ expansion of flavanone 4′-methylene-*O*-*β*-D-(4′′-*O*-methyl)-glucopyranoside (**4a**)

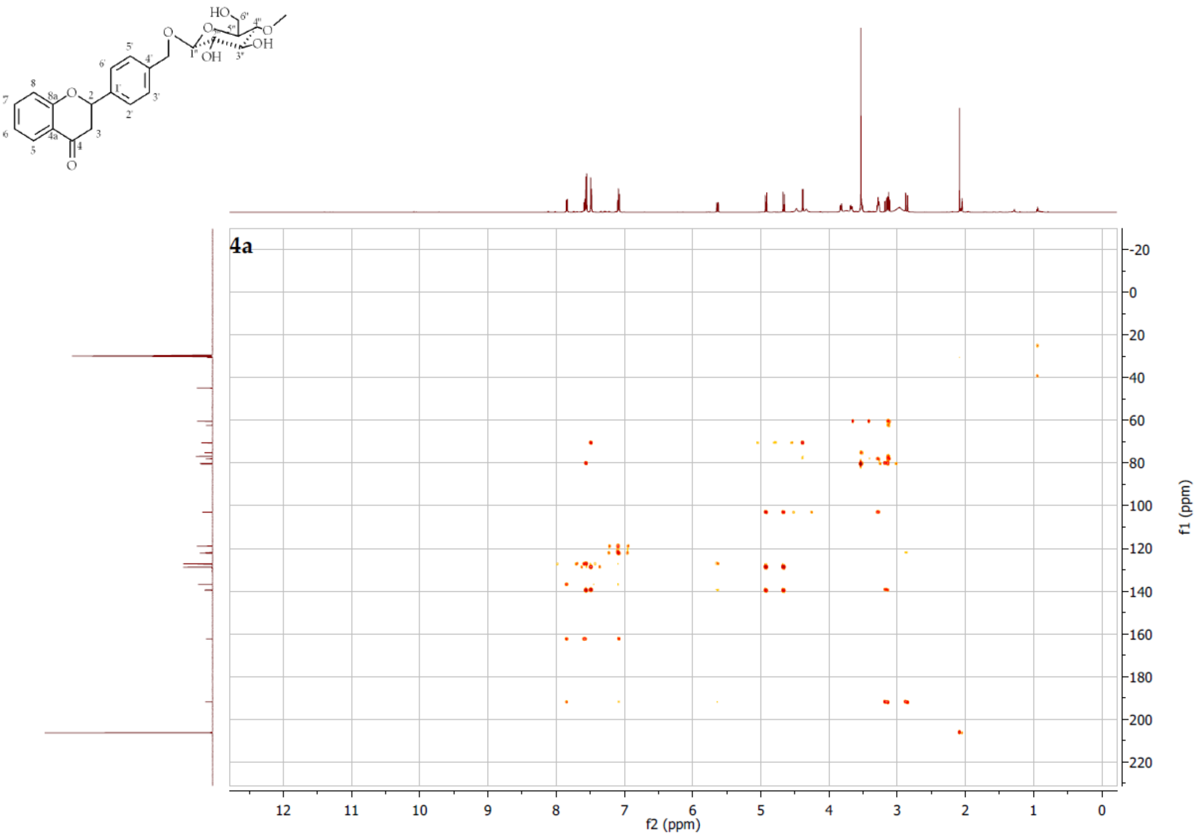

Figure S35. HMBC contour map – $^{1}H \times ^{13}C$ of flavanone 4′-methylene-*O*-*β*-D-(4′′-*O*-methyl)-glucopyranoside (**4a**)

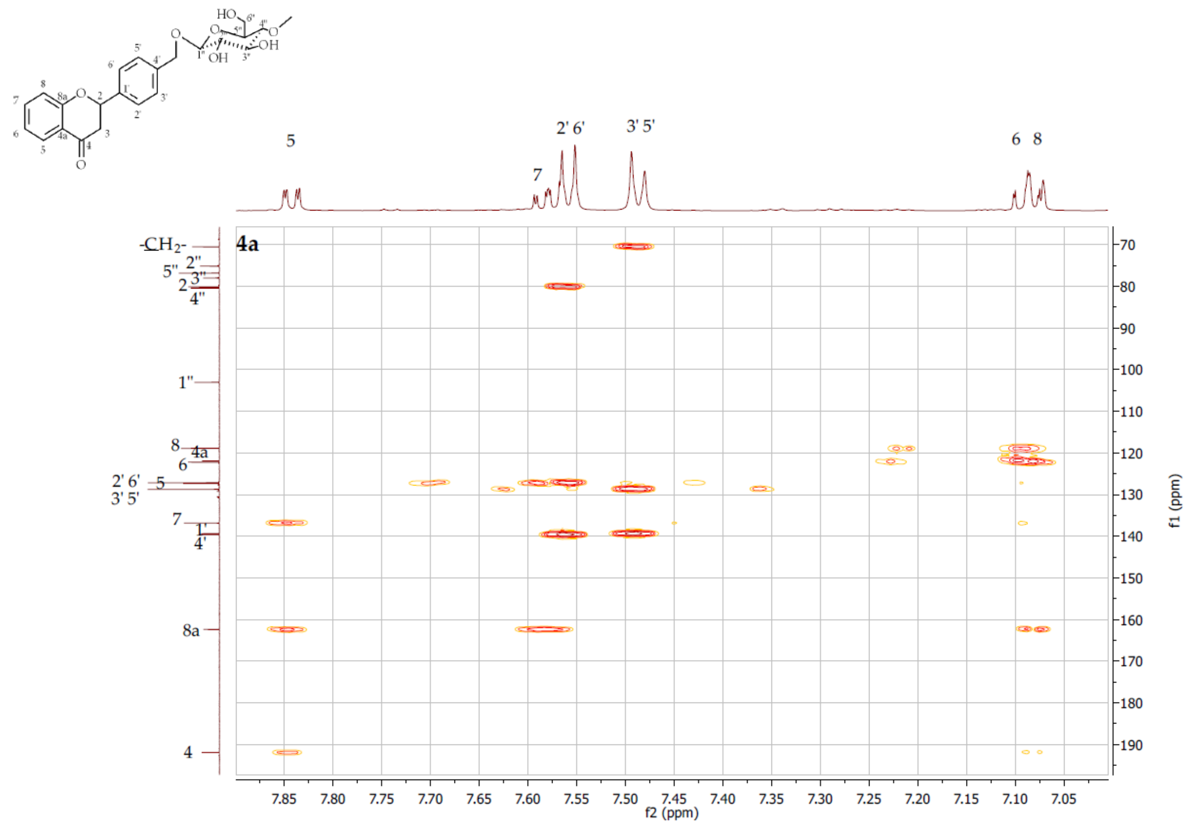

Figure S36. HMBC contour map – ^{1}H x ^{13}C expansion of flavanone 4′-methylene-*O*-*β*-D-(4′′-*O*-methyl)-glucopyranoside (**4a**)

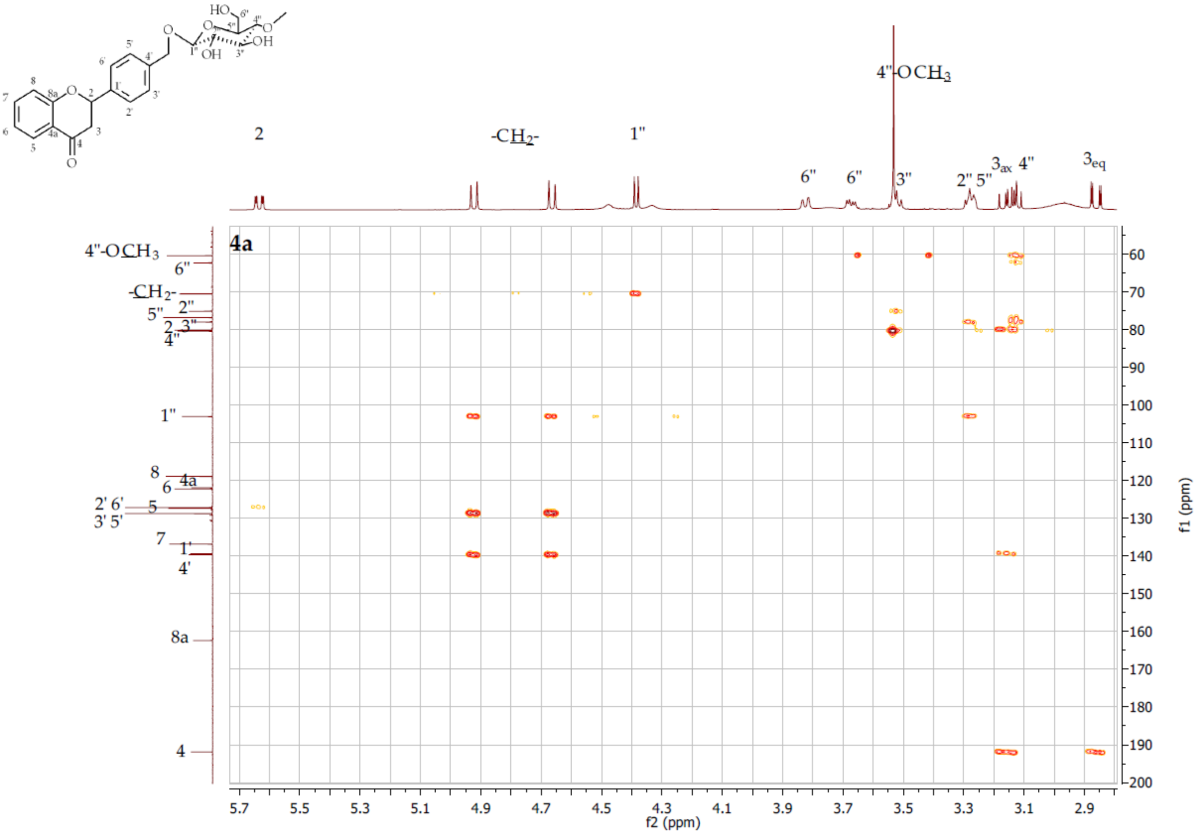

Figure S37. HMBC contour map – ^{1}H x ^{13}C expansion of flavanone 4′-methylene-*O*-*β*-D-(4′′-*O*-methyl)-glucopyranoside (**4a**)

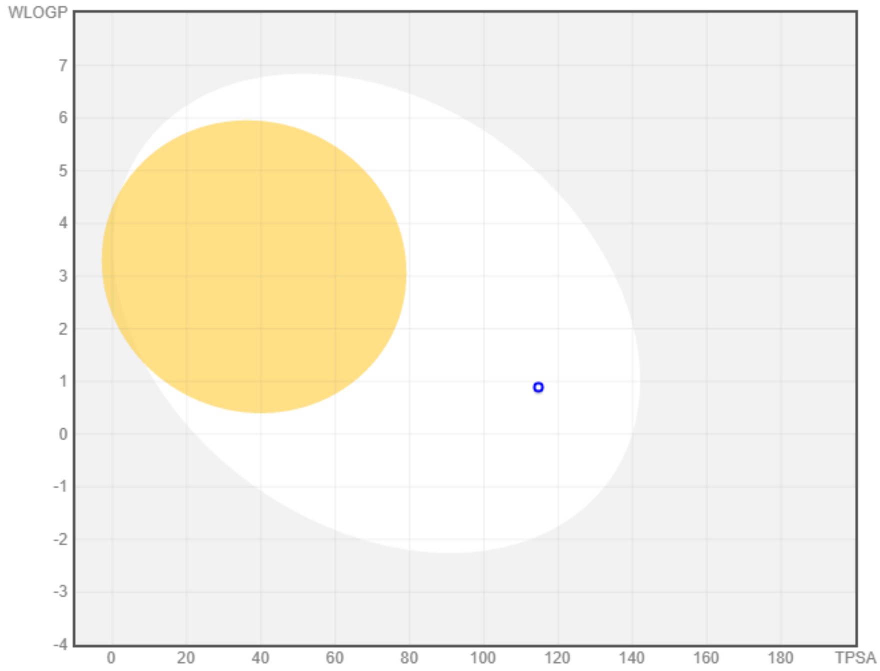

Actions

☐ Show Molecules Name

Legends

BBB

HIA

PGP+

PGP—

Remarks

None

Molecule 1

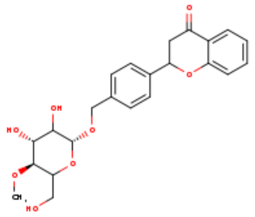

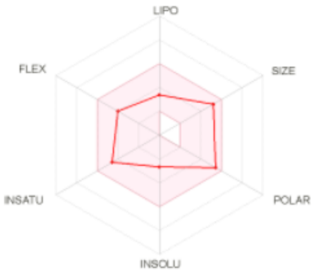

SMILES OCC1O[C@@H](OCc2ccc(cc2)C2CC(=O)c3c(O2)cccc3)C([C@H]([C@@H]1OC)O)O

Physicochemical Properties	
Formula	C23H26O8
Molecular weight	430.45 g/mol
Num. heavy atoms	31
Num. arom. heavy atoms	12
Fraction Csp3	0.43
Num. rotatable bonds	6
Num. H-bond acceptors	8
Num. H-bond donors	3
Molar Refractivity	108.74
TPSA	114.68 Å²
**Lipophilicity**	
Log $P_{o/w}$ (iLOGP)	2.89
Log $P_{o/w}$ (XLOGP3)	0.41
Log $P_{o/w}$ (WLOGP)	0.89
Log $P_{o/w}$ (MLOGP)	-0.24
Log $P_{o/w}$ (SILICOS-IT)	1.77
Consensus Log $P_{o/w}$	1.14

Water Solubility	
Log *S* (ESOL)	-2.66
Solubility	9.47e-01 mg/ml ; 2.20e-03 mol/l
Class	Soluble
Log *S* (Ali)	-2.38
Solubility	1.77e+00 mg/ml ; 4.12e-03 mol/l
Class	Soluble
Log *S* (SILICOS-IT)	-3.97
Solubility	4.66e-02 mg/ml ; 1.08e-04 mol/l
Class	Soluble
**Pharmacokinetics**	
GI absorption	High
BBB permeant	No
P-gp substrate	Yes
CYP1A2 inhibitor	No
CYP2C19 inhibitor	No
CYP2C9 inhibitor	No
CYP2D6 inhibitor	No
CYP3A4 inhibitor	No
Log K_p (skin permeation)	-8.63 cm/s
**Druglikeness**	
Lipinski	Yes; 0 violation
Ghose	Yes
Veber	Yes
Egan	Yes
Muegge	Yes
Bioavailability Score	0.55
**Medicinal Chemistry**	
PAINS	0 alert
Brenk	0 alert
Leadlikeness	No; 1 violation: MW>350
Synthetic accessibility	5.07

Figure S38. flavanone 4′-methylene-*O*-*β*-D-(4′′-*O*-methyl)-glucopyranoside (**4a**) physicochemical and ADME parameters prediction using the SwissADME modelling

Pa	Pi	Activity
0,910	0,002	Hepatoprotectant
0,900	0,010	CDP-glycerol glycerophosphotransferase inhibitor
0,894	0,012	Membrane integrity agonist
0,881	0,004	Antihypercholesterolemic
0,882	0,007	Benzoate-CoA ligase inhibitor
0,870	0,004	Membrane permeability inhibitor
0,866	0,003	Anticarcinogenic
0,848	0,003	Chemopreventive
0,829	0,002	Free radical scavenger
0,830	0,005	CYP3A4 inducer

Figure S39. flavanone 4′-methylene-*O*-*β*-D-(4′′-*O*-methyl)-glucopyranoside (**4a**) biological activity prediction using the Way2Drug Pass online modelling

Name	Confidence	ChEMBL ID
Clostridium ramosum	0.6042	CHEMBL614971
RESISTANT Acinetobacter pittii	0.5626	CHEMBL3140321
Actinomyces meyeri	0.5531	CHEMBL612289
Mycobacterium mageritense	0.5480	CHEMBL612959
Clostridium cadaveris	0.5389	CHEMBL614970
RESISTANT Mycobacterium ulcerans	0.5331	CHEMBL612965
Listeria monocytogenes	0.4524	CHEMBL614974
Nocardia transvalensis	0.4086	CHEMBL613234
Lactobacillus plantarum	0.4052	CHEMBL614973
Staphylococcus lugdunensis	0.3806	CHEMBL613303
Clostridium sordellii	0.3783	CHEMBL613072

Figure S40. flavanone 4′-methylene-*O*-*β*-D-(4′′-*O*-methyl)-glucopyranoside (**4a**) antibacterial activity prediction using the Way2Drug AntiBac-Pred modelling

Name	Confidence	ChEMBL ID
Rhizopus oryzae	0.3701	CHEMBL612306
Absidia corymbifera	0.3405	CHEMBL612369
Candida dubliniensis	0.3324	CHEMBL613334
Trichophyton mentagrophytes	0.2867	CHEMBL613162
Aspergillus niger	0.1721	CHEMBL358
Saccharomyces cerevisiae	0.1242	CHEMBL361
Mucor	0.0718	CHEMBL612521
Penicillium marneffei	0.0311	CHEMBL612994

Figure S41. flavanone 4′-methylene-*O*-*β*-D-(4′′-*O*-methyl)-glucopyranoside (**4a**) antifungal activity prediction using the Way2Drug AntiFun-Pred modelling

Virus	Protein target	Confidence
Severe acute respiratory syndrome coronavirus 2	Replicase polyprotein 1ab	0.5968
Human immunodeficiency virus 2	Human immunodeficiency virus type 2 integrase	0.2298
Influenza A virus	Neuraminidase	0.0202
Varicella-zoster virus (strain Dumas) (HHV-3) (Human herpesvirus 3)	Thymidine kinase	0.0100

Figure S42. flavanone 4′-methylene-*O*-*β*-D-(4′′-*O*-methyl)-glucopyranoside (**4a**) antiviral activity prediction using the Way2Drug AntiVir-Pred modelling

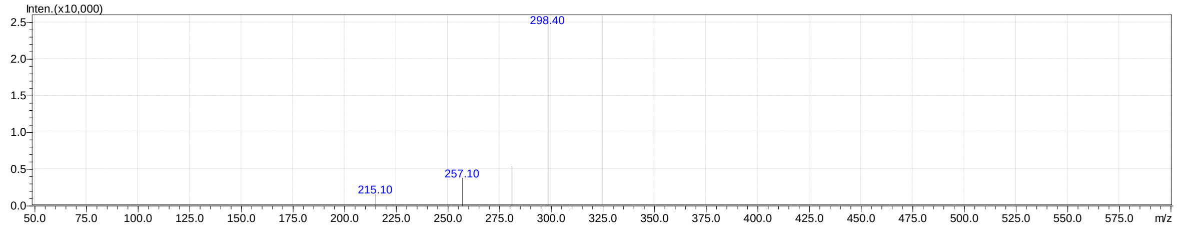

Figure S43. MS analysis of 2-phenyl-(4′-hydroxymethyl)-4-hydroxychromane (**4b**)

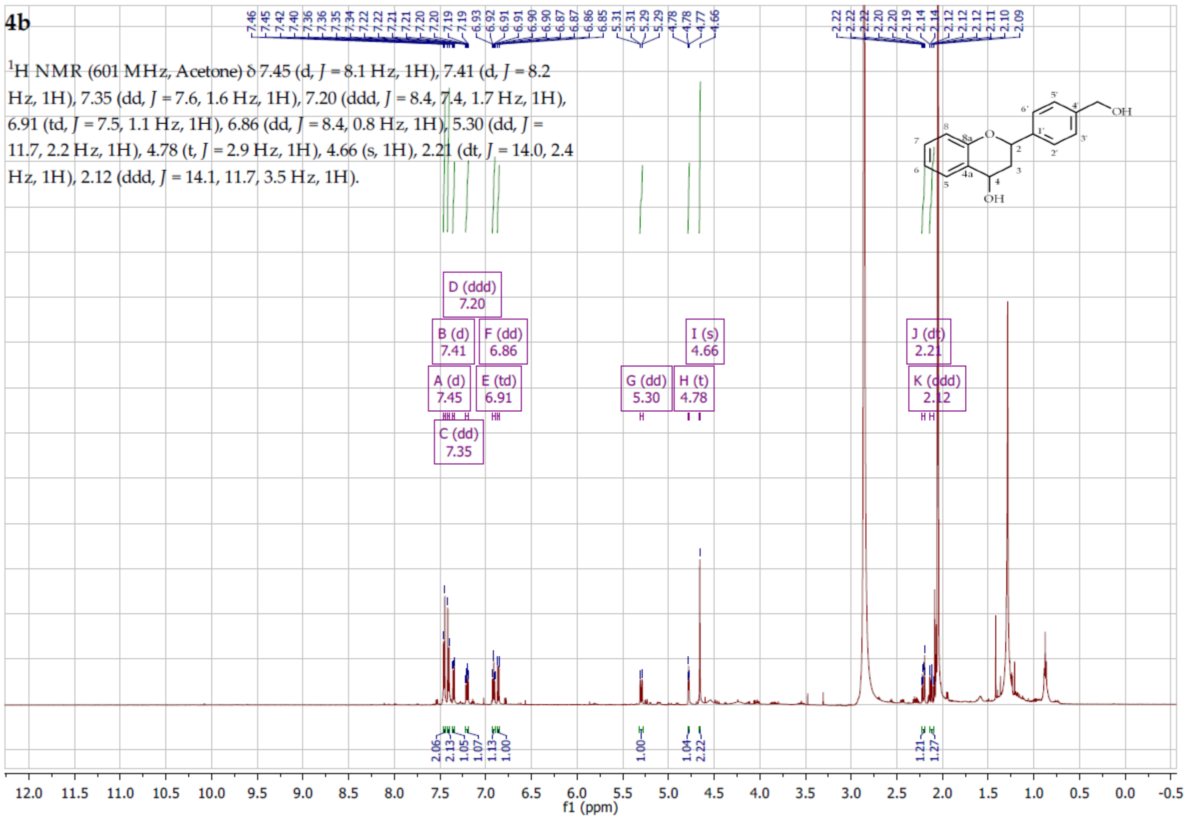

Figure S44. ^{1}H NMR spectrum (*δ*, acetone-d6, 600 MHz) of 2-phenyl-(4′-hydroxymethyl)-4-hydroxychromane (**4b**)

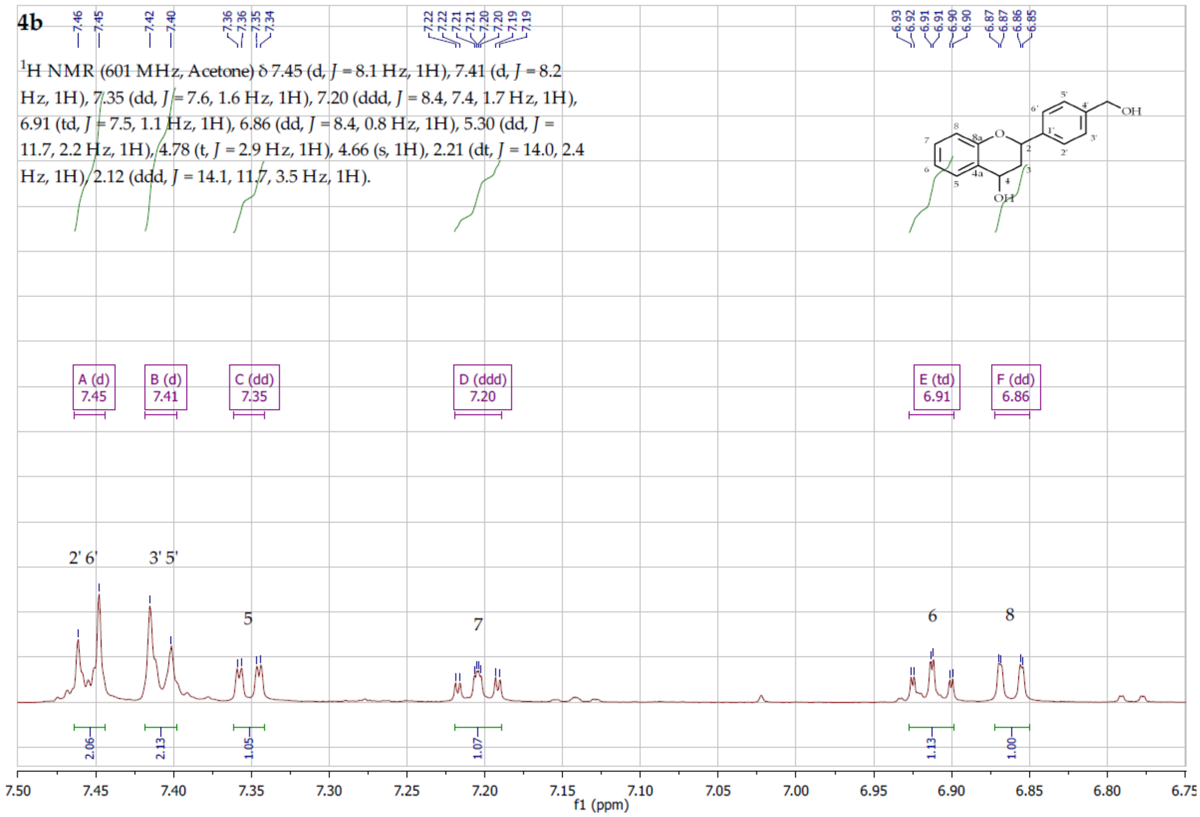

Figure S45. ^{1}H NMR spectrum expansion (*δ,* acetone-d6, 600 MHz) of 2-phenyl-(4′-hydroxymethyl)-4-hydroxychromane (**4b**)

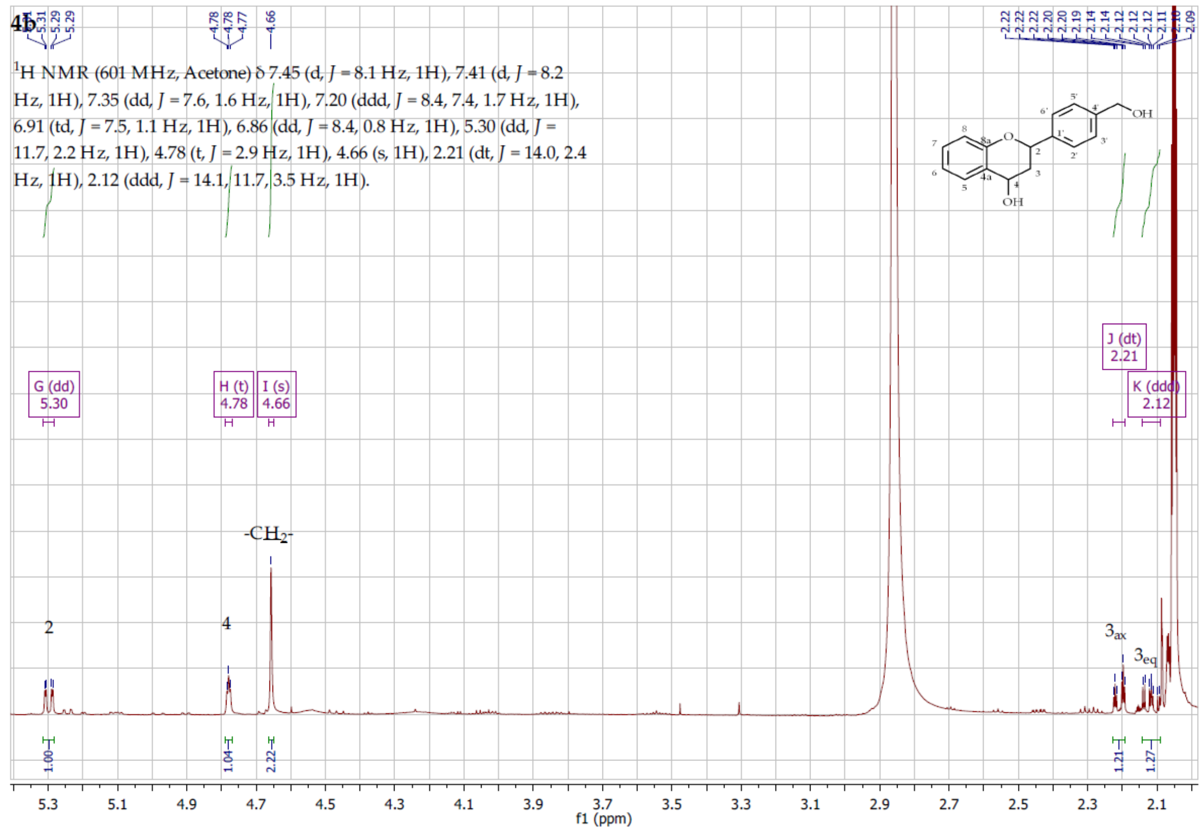

Figure S46. ^{1}H NMR spectrum expansion (*δ,* acetone-d6, 600 MHz) of 2-phenyl-(4′-hydroxymethyl)-4-hydroxychromane (**4b**)

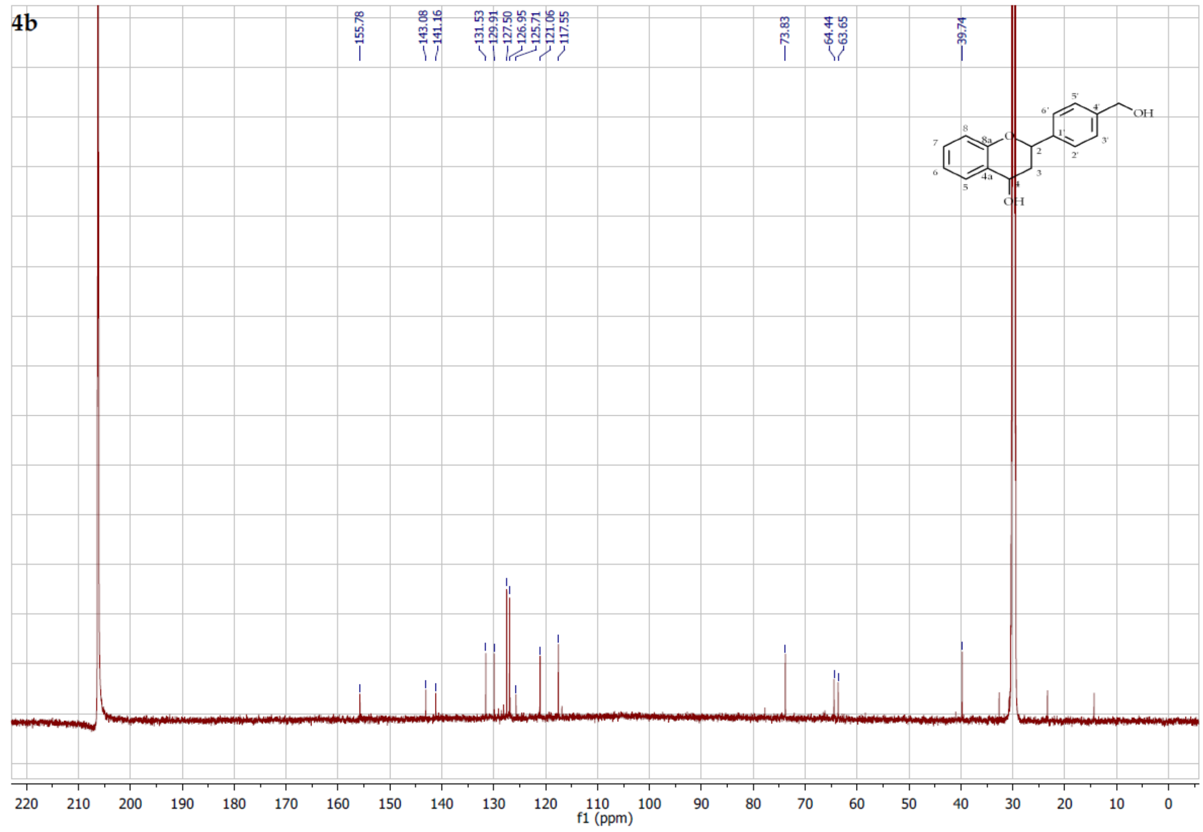

Figure S47. ^{13}C NMR spectrum (δ, acetone-d6, 151 MHz) of 2-phenyl-(4′-hydroxymethyl)-4-hydroxychromane (**4b**)

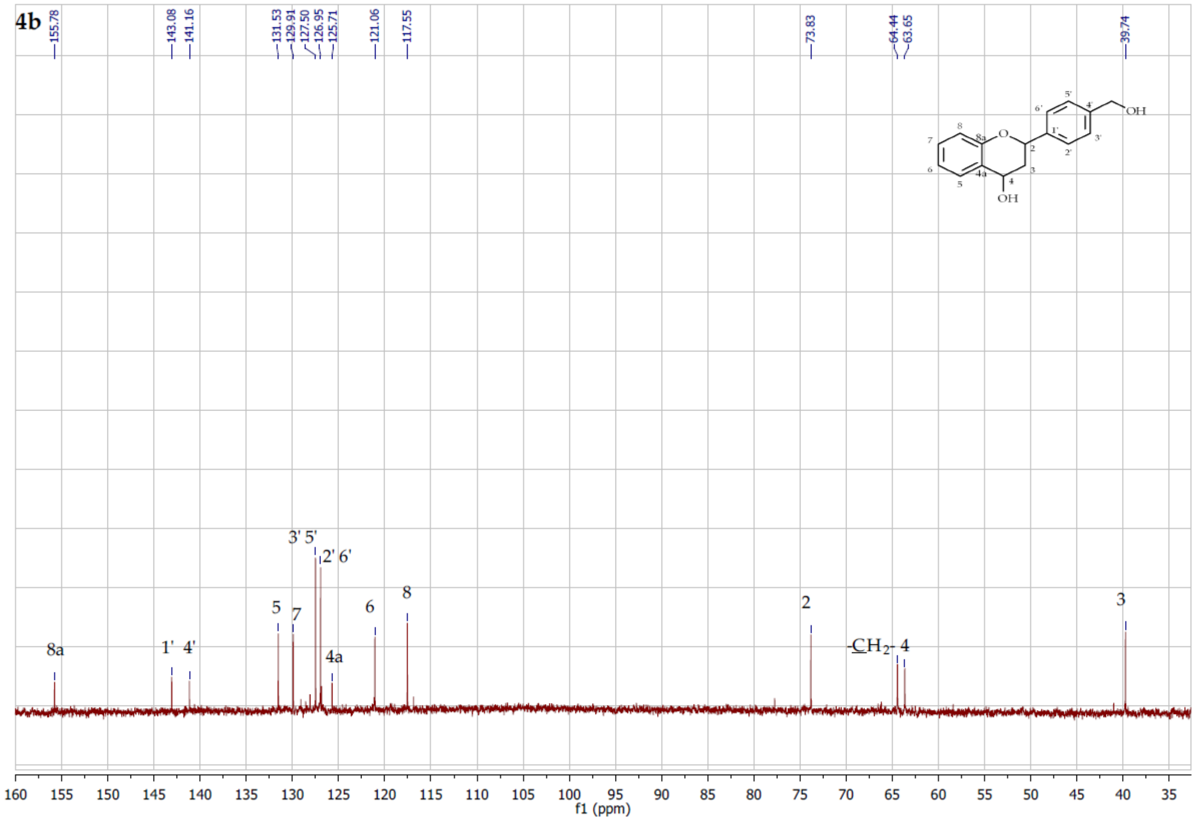

Figure S48. ^{13}C NMR spectrum expansion (δ, acetone-d6, 151 MHz) of 2-phenyl-(4′-hydroxymethyl)-4-hydroxychromane (**4b**)

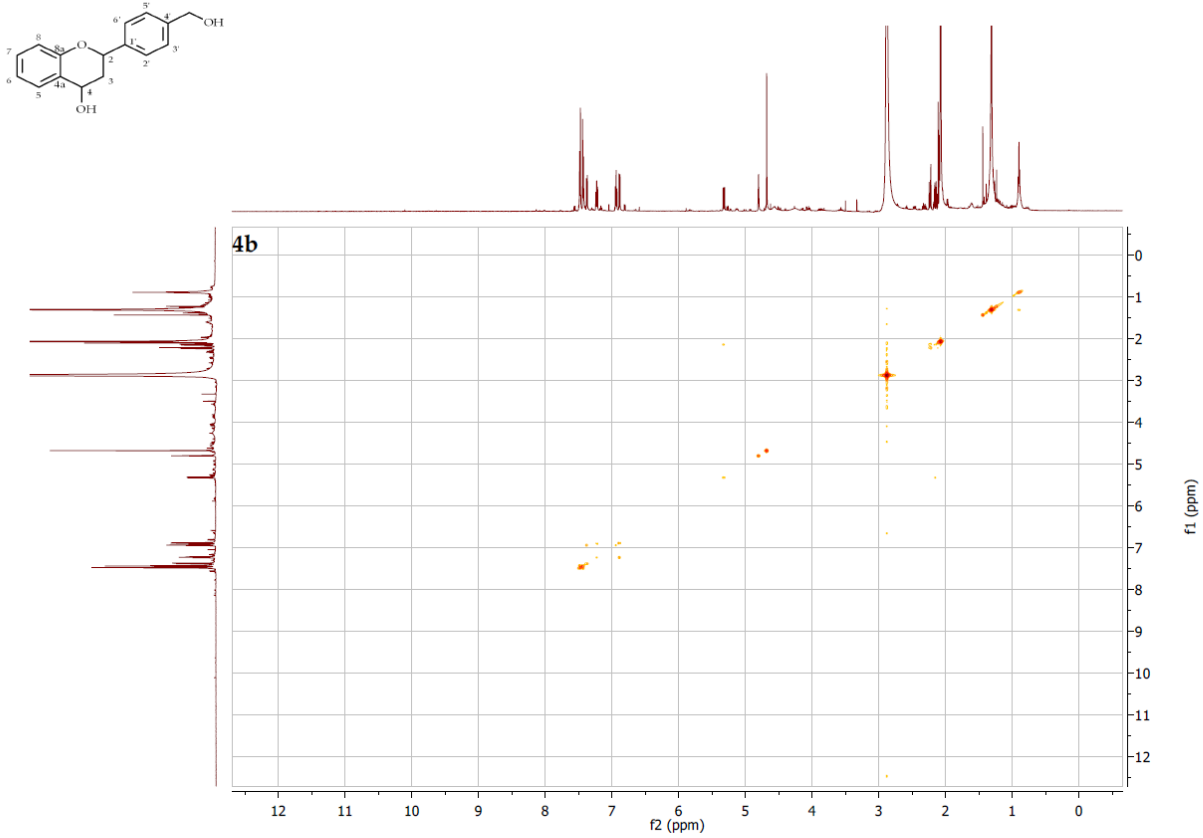

Figure S49. COSY contour map – ^{1}H x ^{1}H of 2-phenyl-(4′-hydroxymethyl)-4-hydroxychromane (**4b**)

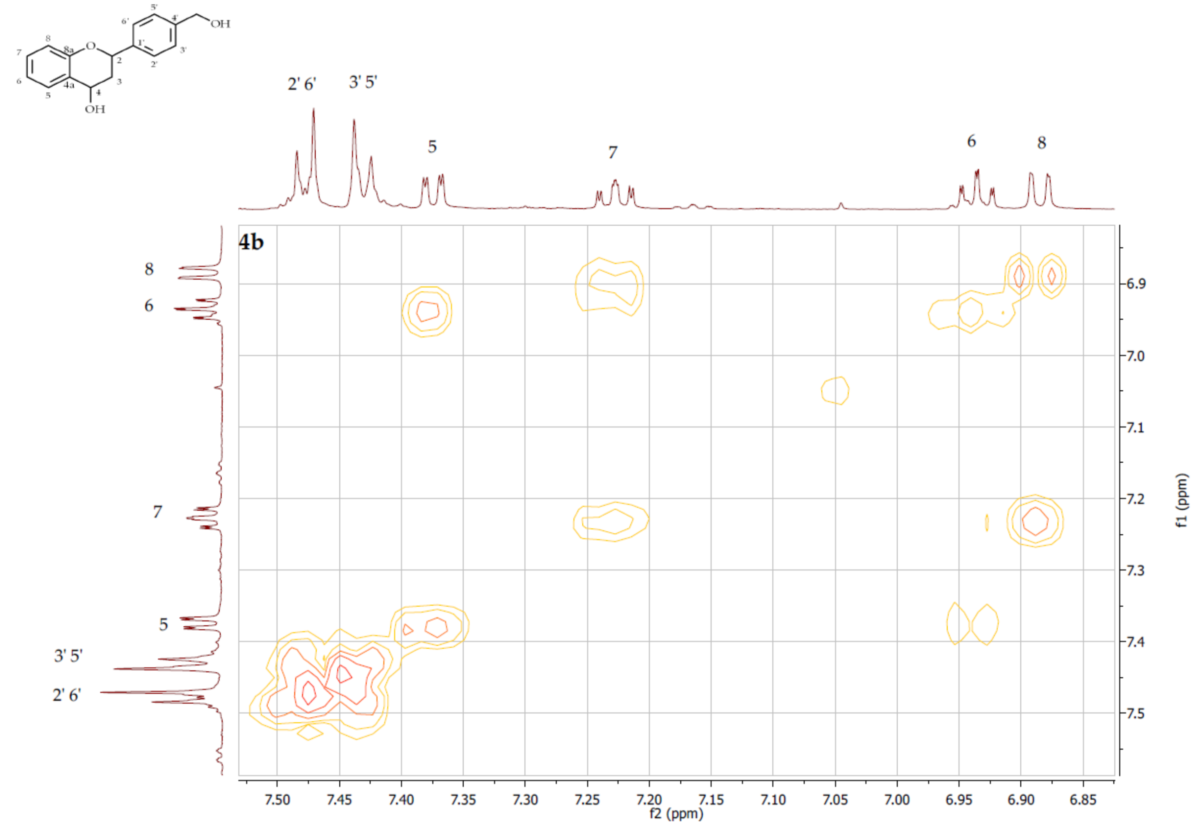

Figure S50. COSY contour map – ^{1}H x ^{1}H expansion of 2-phenyl-(4′-hydroxymethyl)-4-hydroxychromane (**4b**)

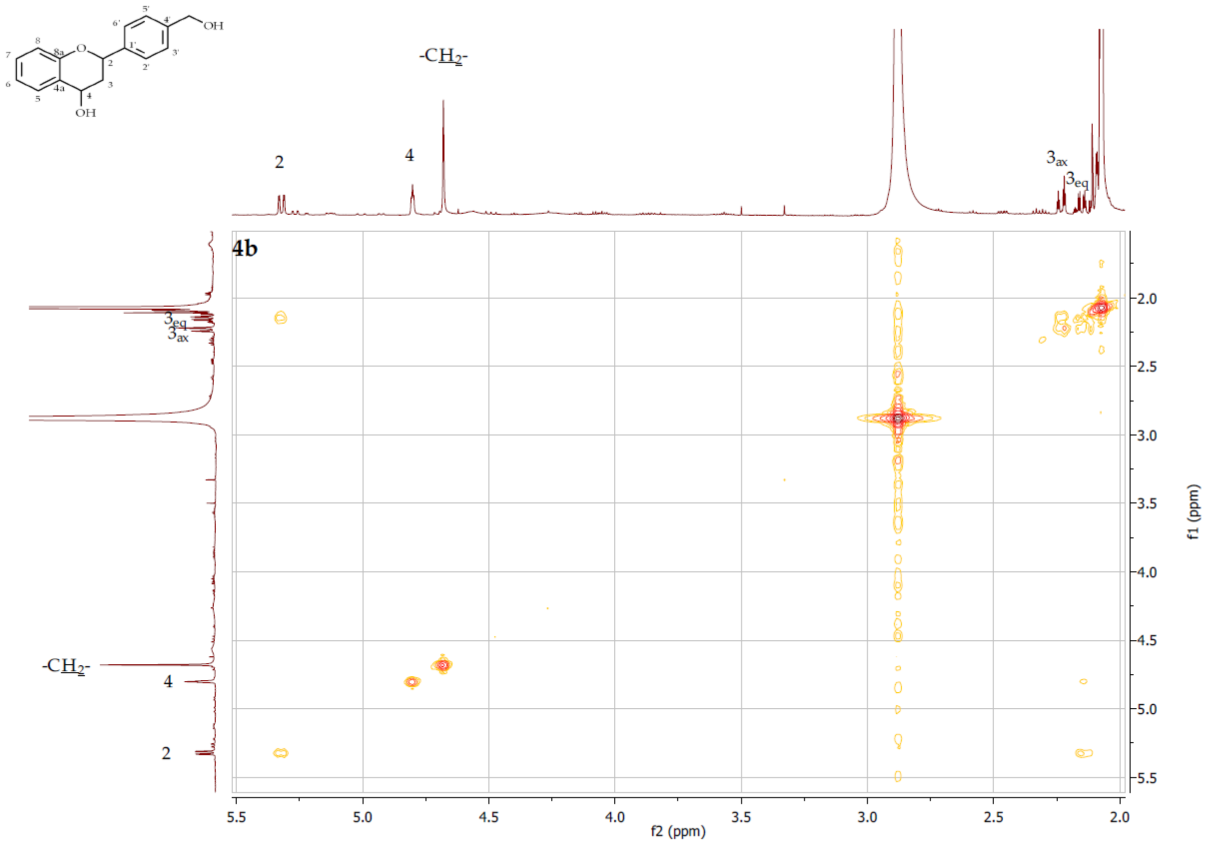

Figure S51. COSY contour map – $^{1}H \times {}^{1}H$ expansion of 2-phenyl-(4′-hydroxymethyl)-4-hydroxychromane (**4b**)

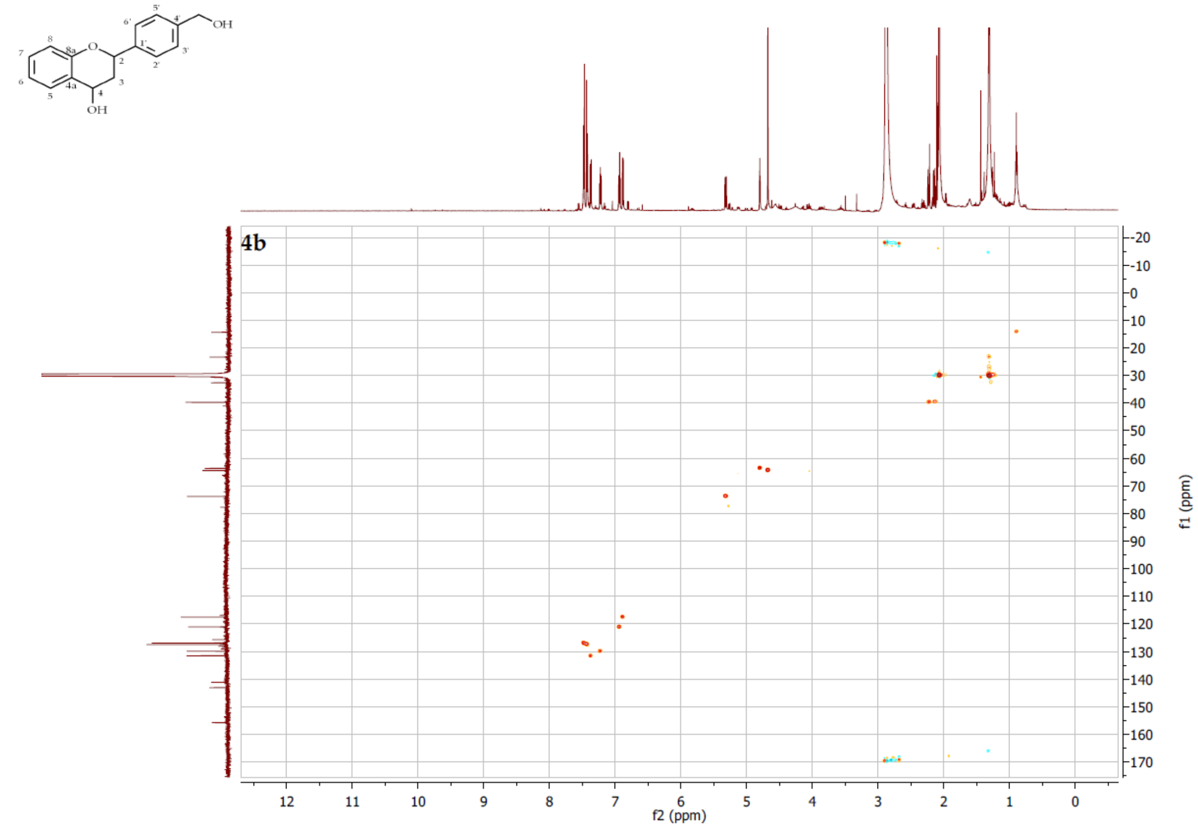

Figure S52. HSQC contour map – $^{1}H \times {}^{13}C$ of 2-phenyl-(4′-hydroxymethyl)-4-hydroxychromane (**4b**)

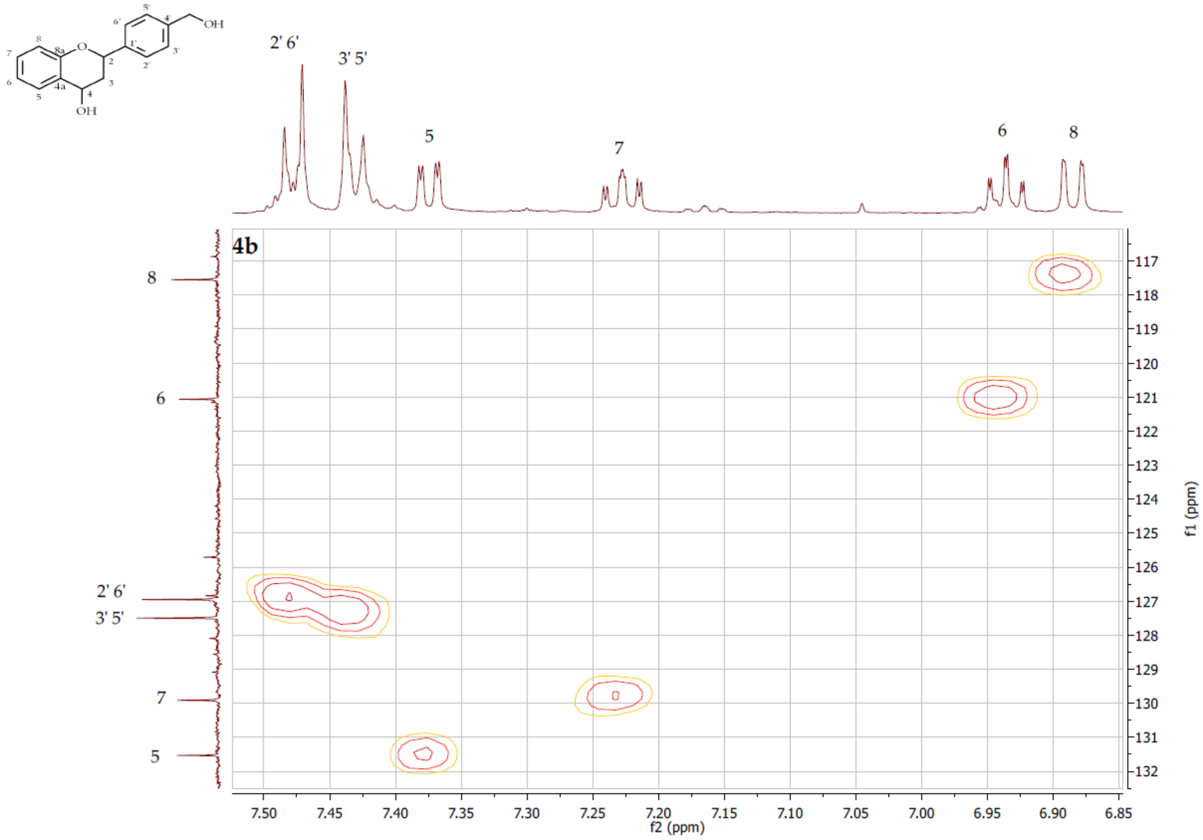

Figure S53. HSQC contour map – ^{1}H x ^{13}C expansion of 2-phenyl-(4′-hydroxymethyl)-4-hydroxychromane (**4b**)

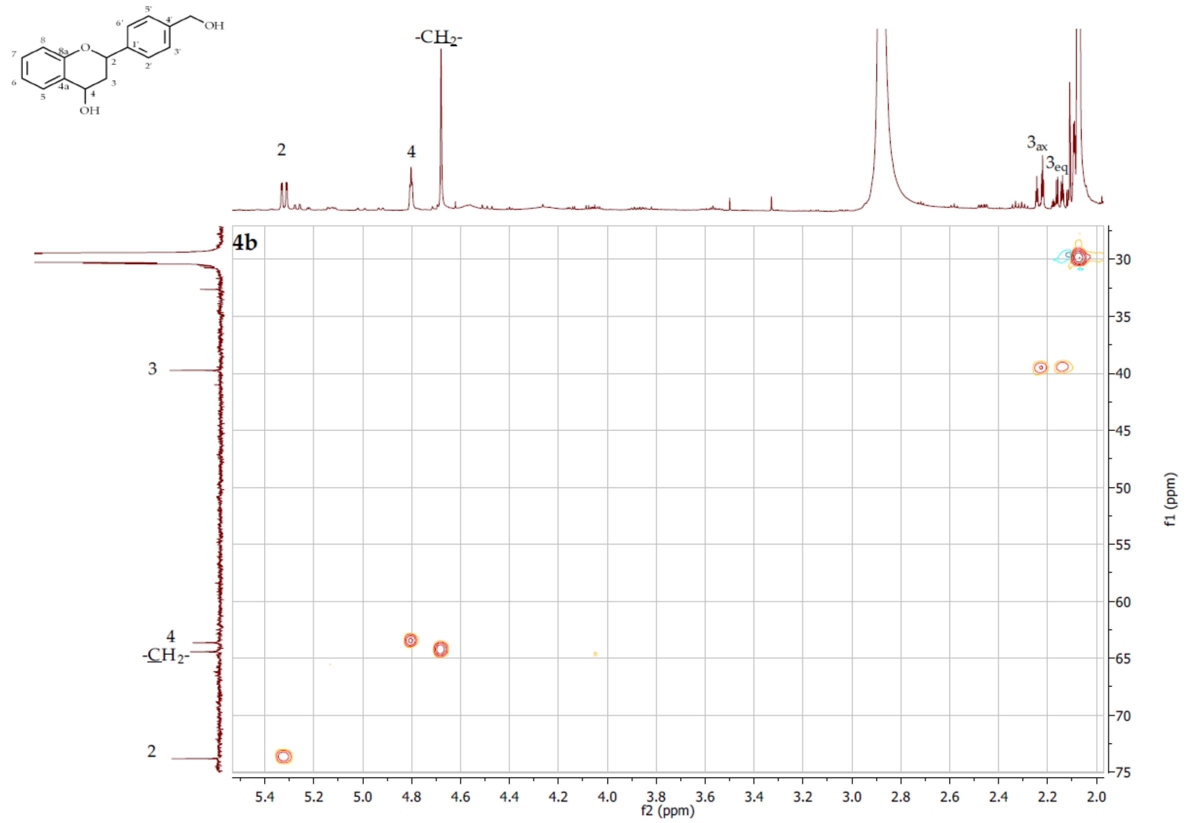

Figure S54. HSQC contour map – ^{1}H x ^{13}C expansion of 2-phenyl-(4′-hydroxymethyl)-4-hydroxychromane (**4b**)

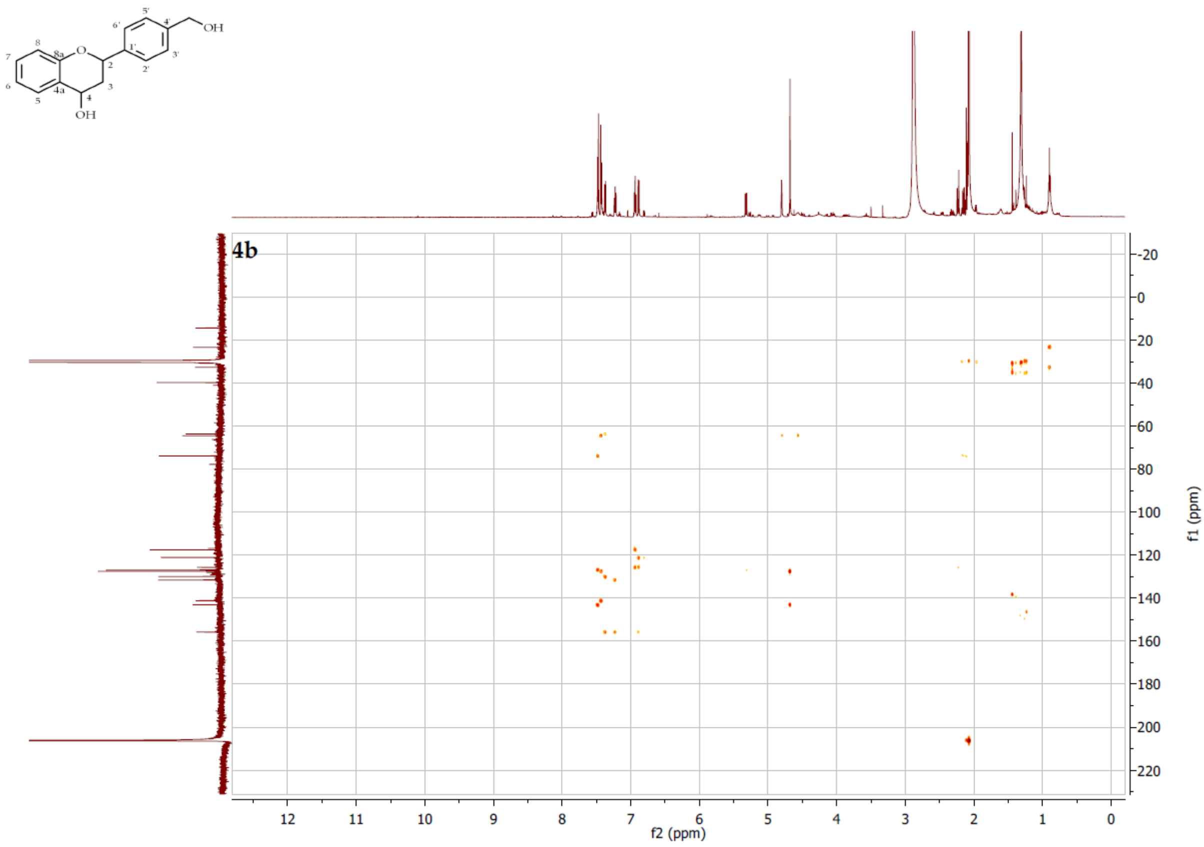

Figure S55. HMBC contour map – ^{1}H x ^{13}C 2-phenyl-(4′-hydroxymethyl)-4-hydroxychromane (**4b**)

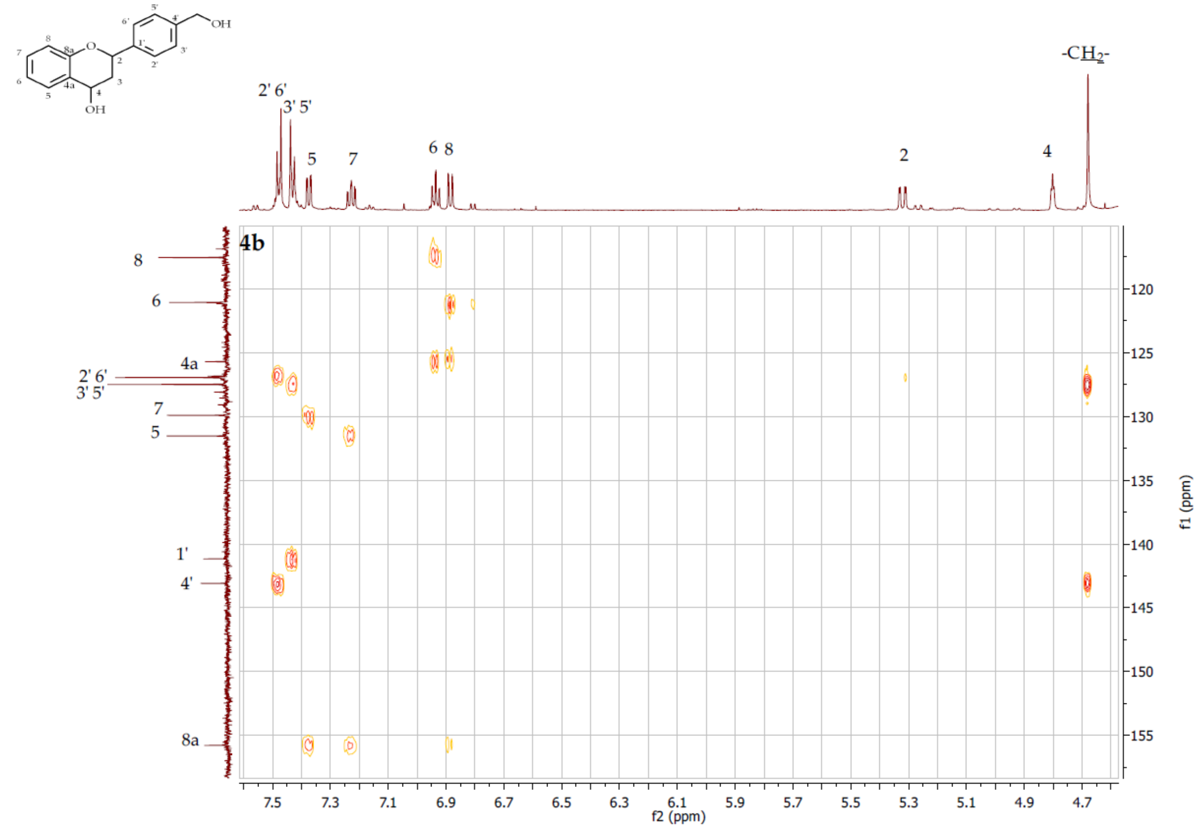

Figure S56. HMBC contour map – ^{1}H x ^{13}C expansion of 2-phenyl-(4′-hydroxymethyl)-4-hydroxychromane (**4b**)

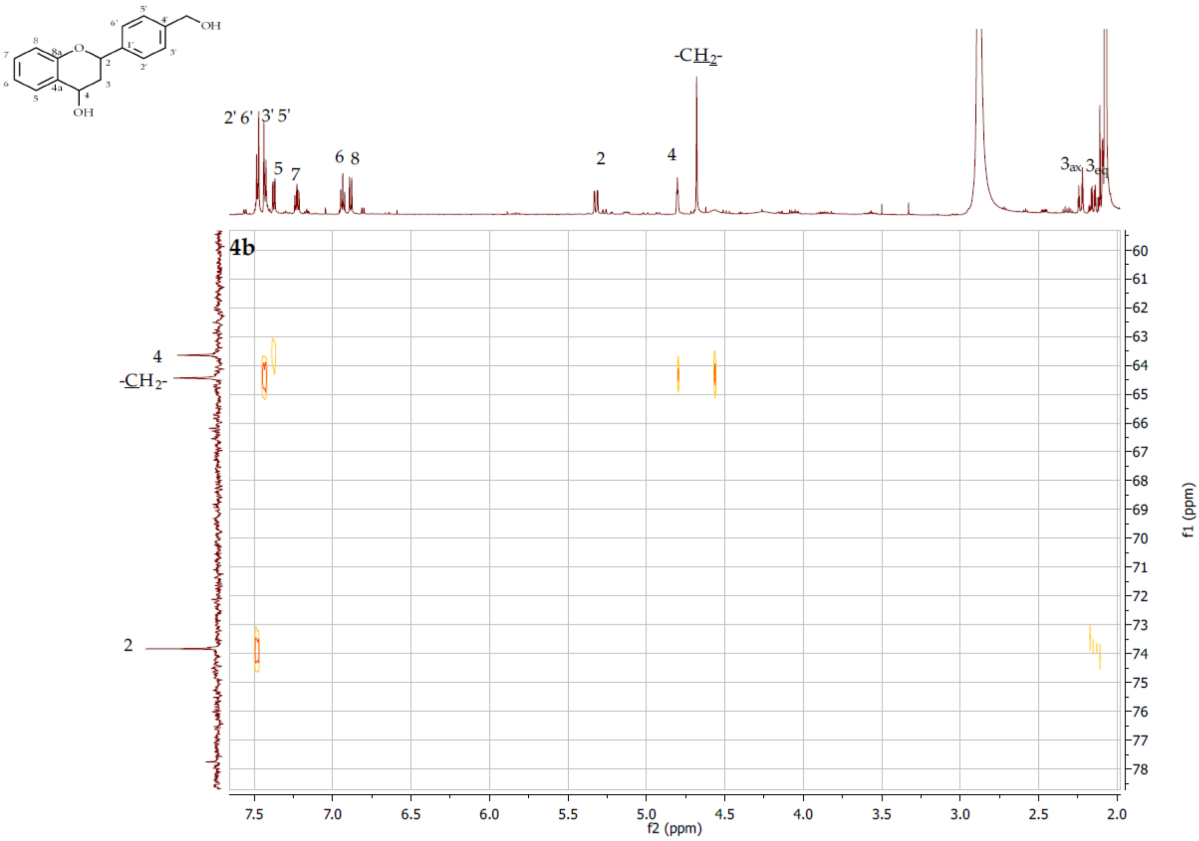

Figure S57. HMBC contour map – ^{1}H x ^{13}C expansion of 2-phenyl-(4′-hydroxymethyl)-4-hydroxychromane (**4b**)

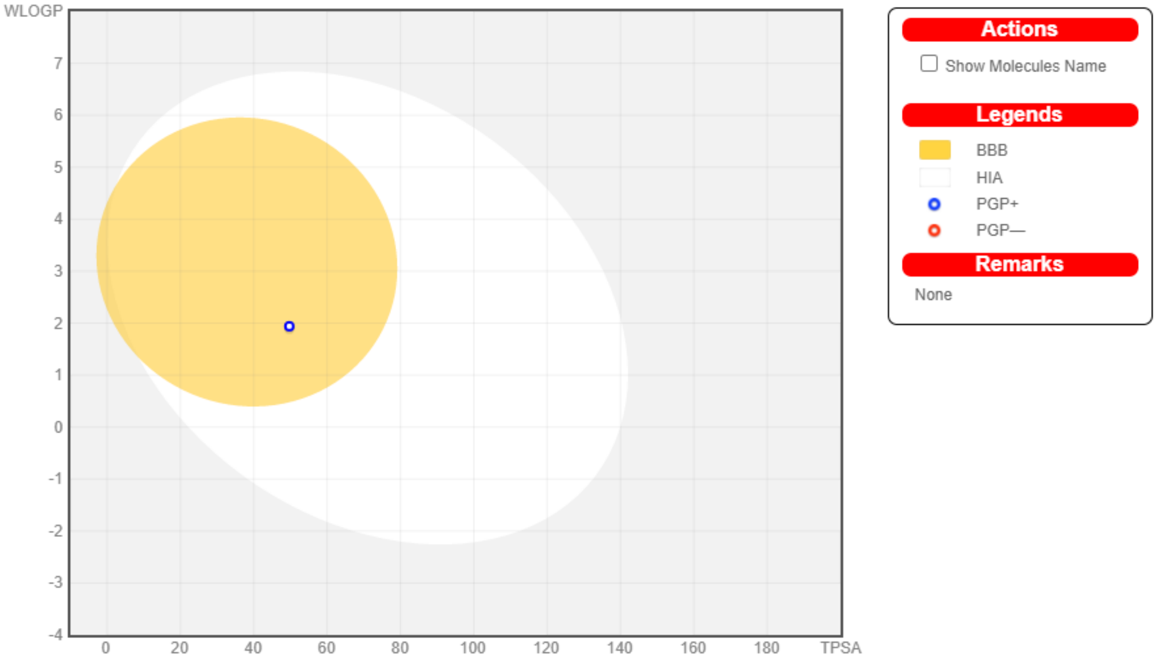

Molecule 1

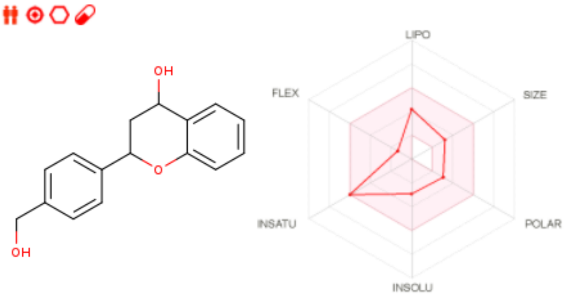

SMILES OCc1ccc(cc1)C1CC(O)c2c(O1)cccc2

Physicochemical Properties	
Formula	C16H16O3
Molecular weight	256.30 g/mol
Num. heavy atoms	19
Num. arom. heavy atoms	12
Fraction Csp3	0.25
Num. rotatable bonds	2
Num. H-bond acceptors	3
Num. H-bond donors	2
Molar Refractivity	72.37
TPSA	49.69 Å²

Lipophilicity	
Log $P_{o/w}$ (iLOGP)	2.20
Log $P_{o/w}$ (XLOGP3)	1.82
Log $P_{o/w}$ (WLOGP)	1.94
Log $P_{o/w}$ (MLOGP)	1.91
Log $P_{o/w}$ (SILICOS-IT)	2.82
Consensus Log $P_{o/w}$	2.14

Water Solubility	
Log *S* (ESOL)	-2.91
Solubility	3.15e-01 mg/ml ; 1.23e-03 mol/l
Class	Soluble
Log *S* (Ali)	-2.48
Solubility	8.42e-01 mg/ml ; 3.29e-03 mol/l
Class	Soluble
Log *S* (SILICOS-IT)	-4.28
Solubility	1.34e-02 mg/ml ; 5.22e-05 mol/l
Class	Moderately soluble

Pharmacokinetics	
GI absorption	High
BBB permeant	Yes
P-gp substrate	Yes
CYP1A2 inhibitor	No
CYP2C19 inhibitor	No
CYP2C9 inhibitor	No
CYP2D6 inhibitor	Yes
CYP3A4 inhibitor	No
Log K_p (skin permeation)	-6.57 cm/s

Druglikeness	
Lipinski	Yes; 0 violation
Ghose	Yes
Veber	Yes
Egan	Yes
Muegge	Yes
Bioavailability Score	0.55

Medicinal Chemistry	
PAINS	0 alert
Brenk	0 alert
Leadlikeness	Yes
Synthetic accessibility	3.22

Figure S58. 2-Phenyl-(4′-hydroxymethyl)-4-hydroxychromane (**4b**) physicochemical and ADME parameters prediction using the SwissADME modelling

Pa	Pi	Activity
0,908	0,009	Membrane integrity agonist
0,828	0,006	Pullulanase inhibitor
0,815	0,014	Sugar-phosphatase inhibitor
0,805	0,017	Alkenylglycerophosphocholine hydrolase inhibitor
0,779	0,041	CYP2C12 substrate
0,741	0,014	Glucan endo-1,6-beta-glucosidase inhibitor
0,736	0,012	UDP-glucuronosyltransferase substrate
0,728	0,007	Glucan 1,4-alpha-maltotriohydrolase inhibitor
0,736	0,015	Ribulose-phosphate 3-epimerase inhibitor
0,729	0,027	Methylenetetrahydrofolate reductase (NADPH) inhibitor

Figure S59. 2-Phenyl-(4′-hydroxymethyl)-4-hydroxychromane (**4b**) biological activity prediction using the Way2Drug Pass online modelling

Name	Confidence	ChEMBL ID
Staphylococcus haemolyticus	0.5987	CHEMBL612507
Listeria monocytogenes	0.4041	CHEMBL614974
Mycobacterium mageritense	0.3465	CHEMBL612959
Clostridium cadaveris	0.3428	CHEMBL614970
RESISTANT Acinetobacter pittii	0.3205	CHEMBL3140321
Clostridium ramosum	0.3178	CHEMBL614971
Bacillus subtilis	0.2989	CHEMBL359
Pseudomonas fluorescens	0.2824	CHEMBL612500
Yersinia pestis	0.2822	CHEMBL614597
Propionibacterium acnes	0.2668	CHEMBL612639
RESISTANT Burkholderia pseudomallei	0.2585	CHEMBL3140323
RESISTANT Mycobacterium ulcerans	0.2406	CHEMBL612965

Figure S60. 2-Phenyl-(4′-hydroxymethyl)-4-hydroxychromane (**4b**) antibacterial activity prediction using the Way2Drug AntiBac-Pred modelling

Name	Confidence	ChEMBL ID
Candida dubliniensis	0.7041	CHEMBL613334
Rhizopus oryzae	0.3998	CHEMBL612306
Absidia corymbifera	0.3508	CHEMBL612369
Yarrowia lipolytica	0.3209	CHEMBL612844
Mucor	0.3015	CHEMBL612521
Trichophyton mentagrophytes	0.2976	CHEMBL613162
Epidermophyton floccosum	0.2495	CHEMBL612386

Figure S61. 2-Phenyl-(4′-hydroxymethyl)-4-hydroxychromane (**4b**) antifungal activity prediction using the Way2Drug AntiFun-Pred modelling

Virus	Protein target	Confidence
Human immunodeficiency virus 2	Human immunodeficiency virus type 2 integrase	0.3653
Severe acute respiratory syndrome coronavirus 2	Replicase polyprotein 1ab	0.2621
Varicella-zoster virus<br>(strain Dumas)<br>(HHV-3)<br>(Human herpesvirus 3)	DNA polymerase	0.1877
Herpes simplex virus<br>(type 1 / strain 17)	Human herpesvirus 1 DNA polymerase	0.1877
Dengue virus type 2	Genome polyprotein	0.1222
Macacine herpesvirus 1	Thymidine kinase	0.0887
Human immunodeficiency virus type 1 group M subtype B<br>(isolateARV2/SF2)<br>(HIV-1)	Gag-Pol polyprotein	0.0050

Figure S62. 2-Phenyl-(4′-hydroxymethyl)-4-hydroxychromane (**4b**) antiviral activity prediction using the Way2Drug AntiVir-Pred modelling

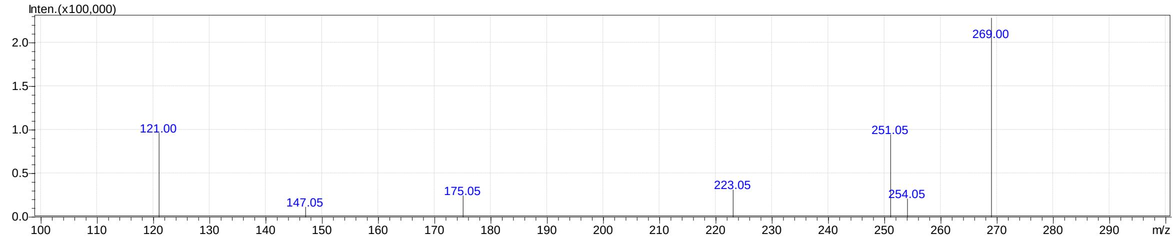

Figure S63. MS analysis of flavanone 4′-carboxylic acid (**4c**)

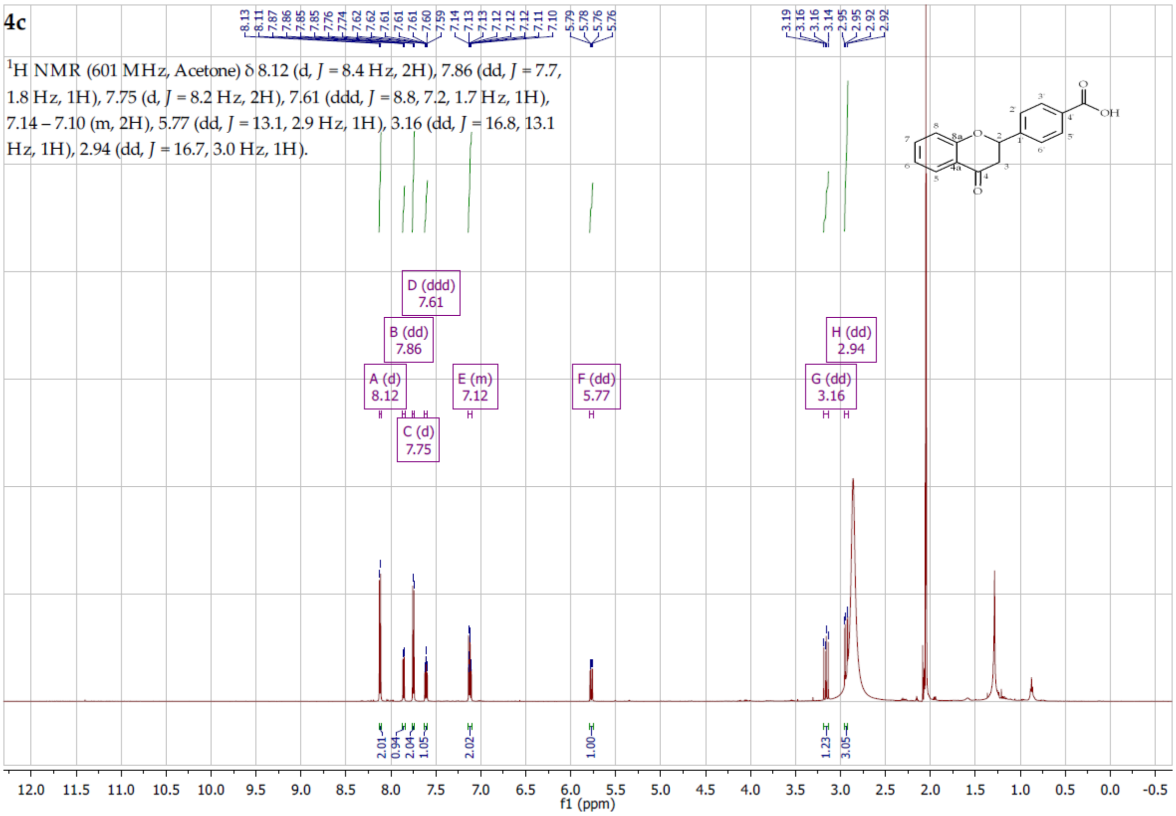

Figure S64. ^{1}H NMR spectrum (δ, acetone-d6, 600 MHz) of flavanone 4′-carboxylic acid (**4c**)

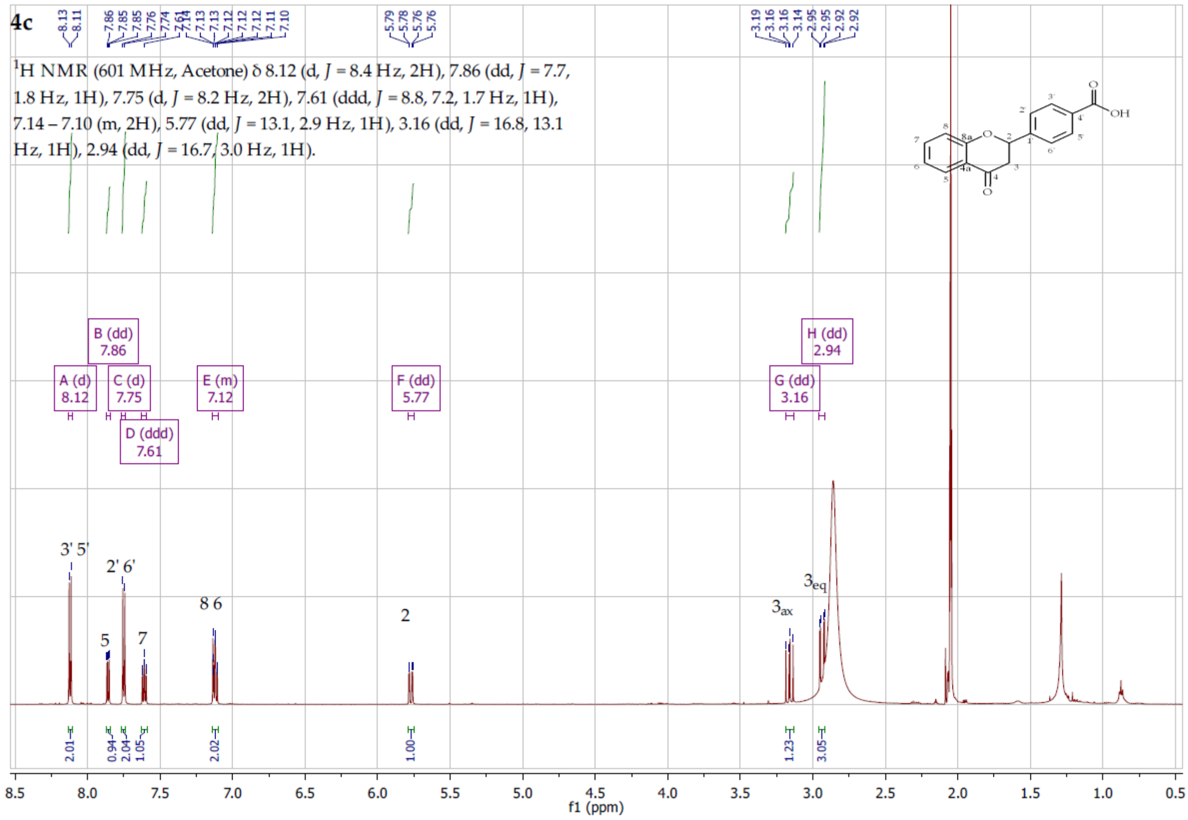

Figure S65. ^{1}H NMR spectrum expansion (δ, acetone-d6, 600 MHz) of flavanone 4′-carboxylic acid (**4c**)

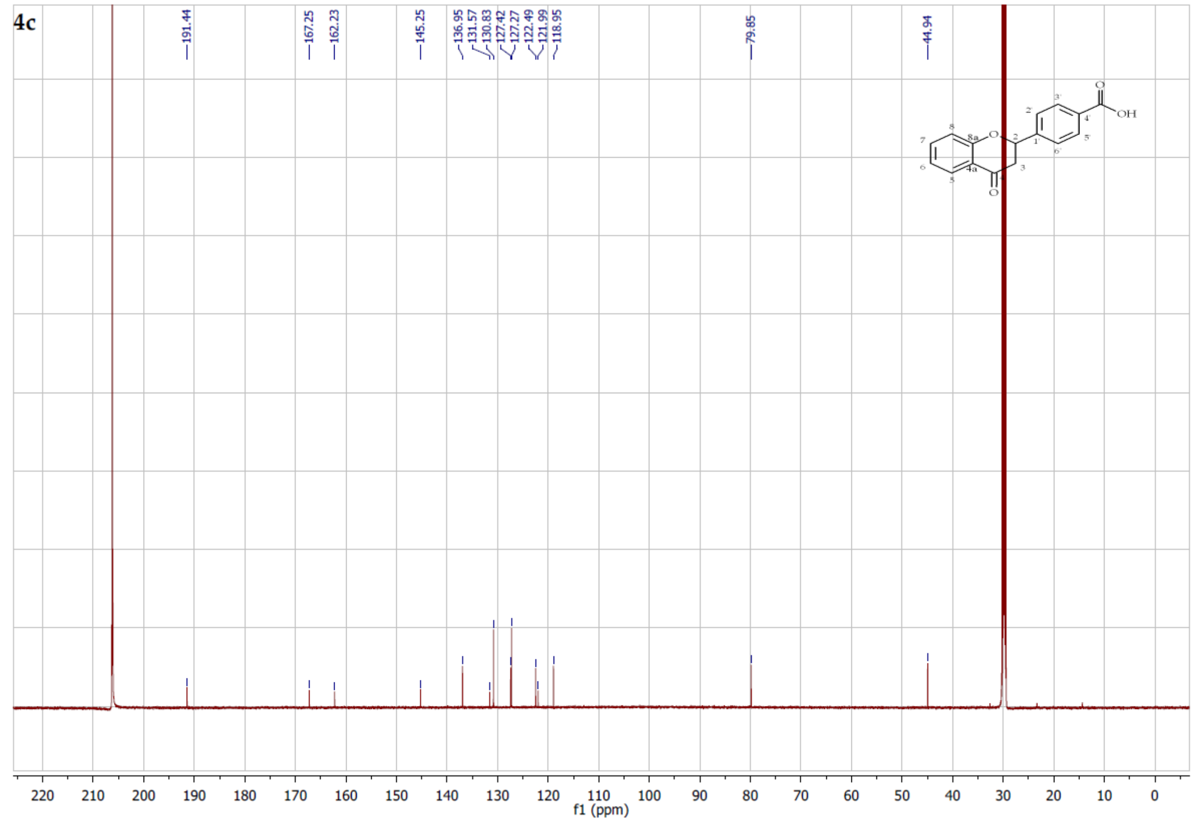

Figure S66. ^{13}C NMR spectrum (δ, acetone-d6, 151 MHz) of flavanone 4′-carboxylic acid (**4c**)

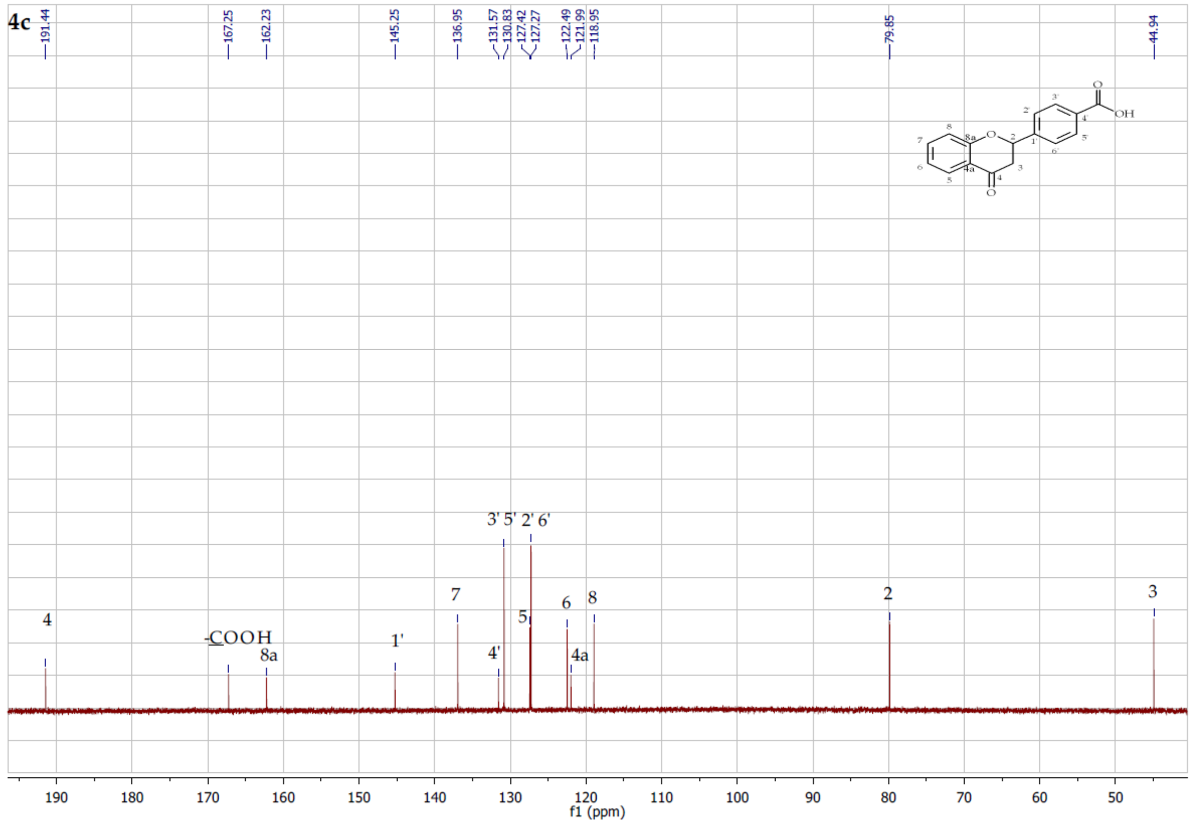

Figure S67. ^{13}C NMR spectrum expansion (δ, acetone-d6, 151 MHz) of flavanone 4’-carboxylic acid (**4c**)

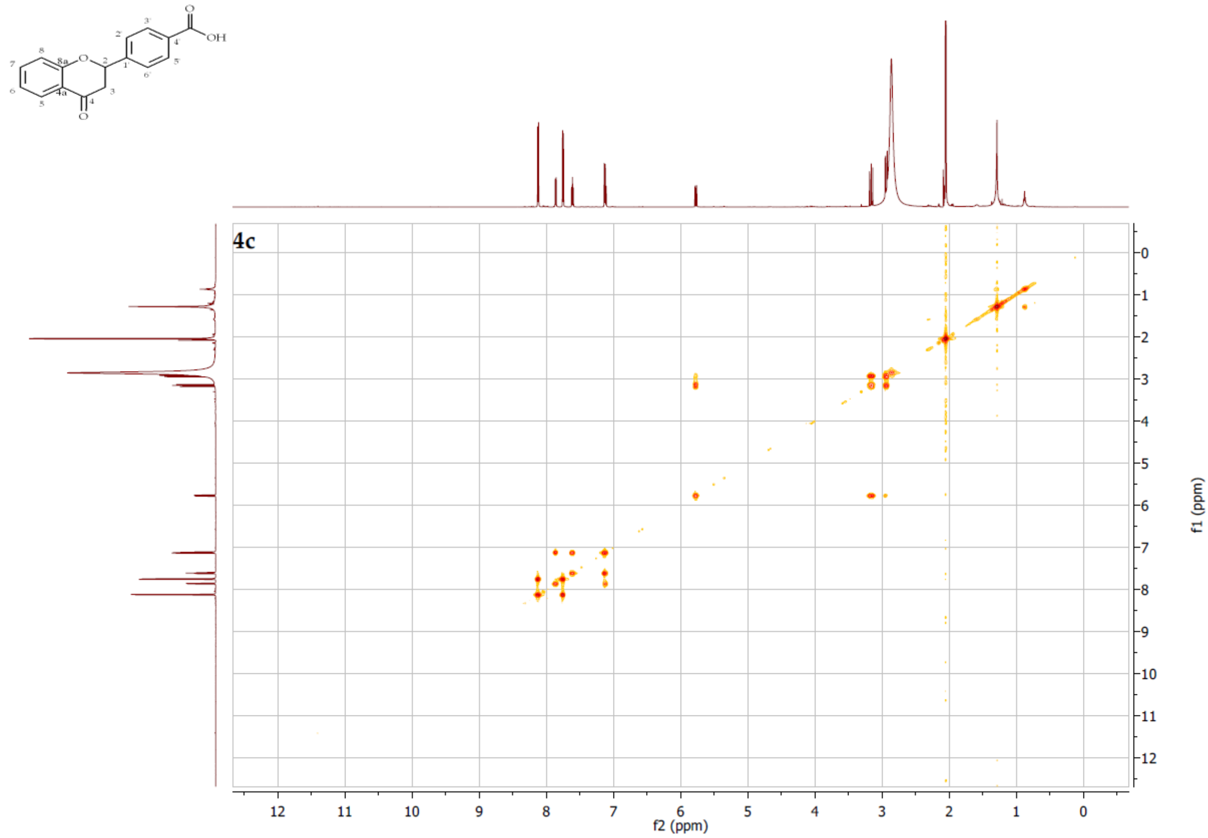

Figure S68. COSY contour map – ^{1}H x ^{1}H of flavanone 4’-carboxylic acid (**4c**)

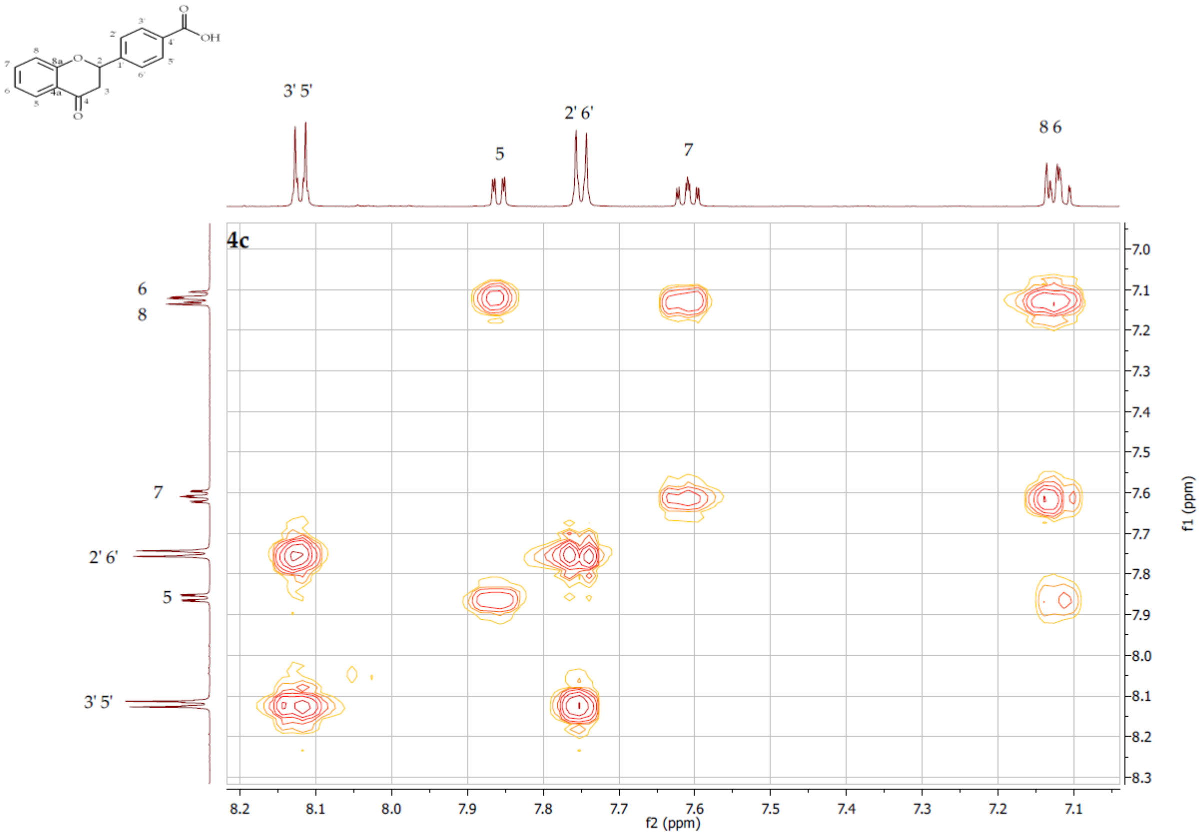

Figure S69. COSY contour map – ^{1}H x ^{1}H expansion of flavanone 4′-carboxylic acid (**4c**)

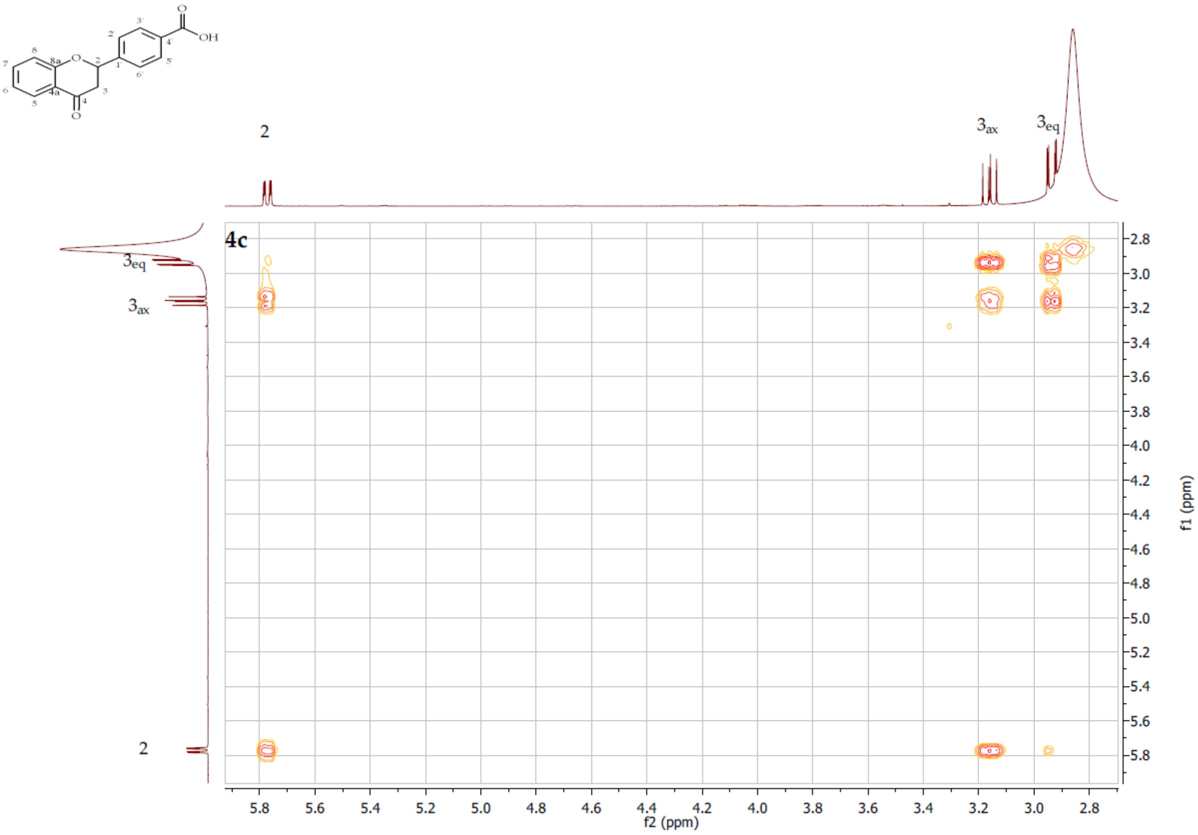

Figure S70. COSY contour map – ^{1}H x ^{1}H expansion of flavanone 4′-carboxylic acid (**4c**)

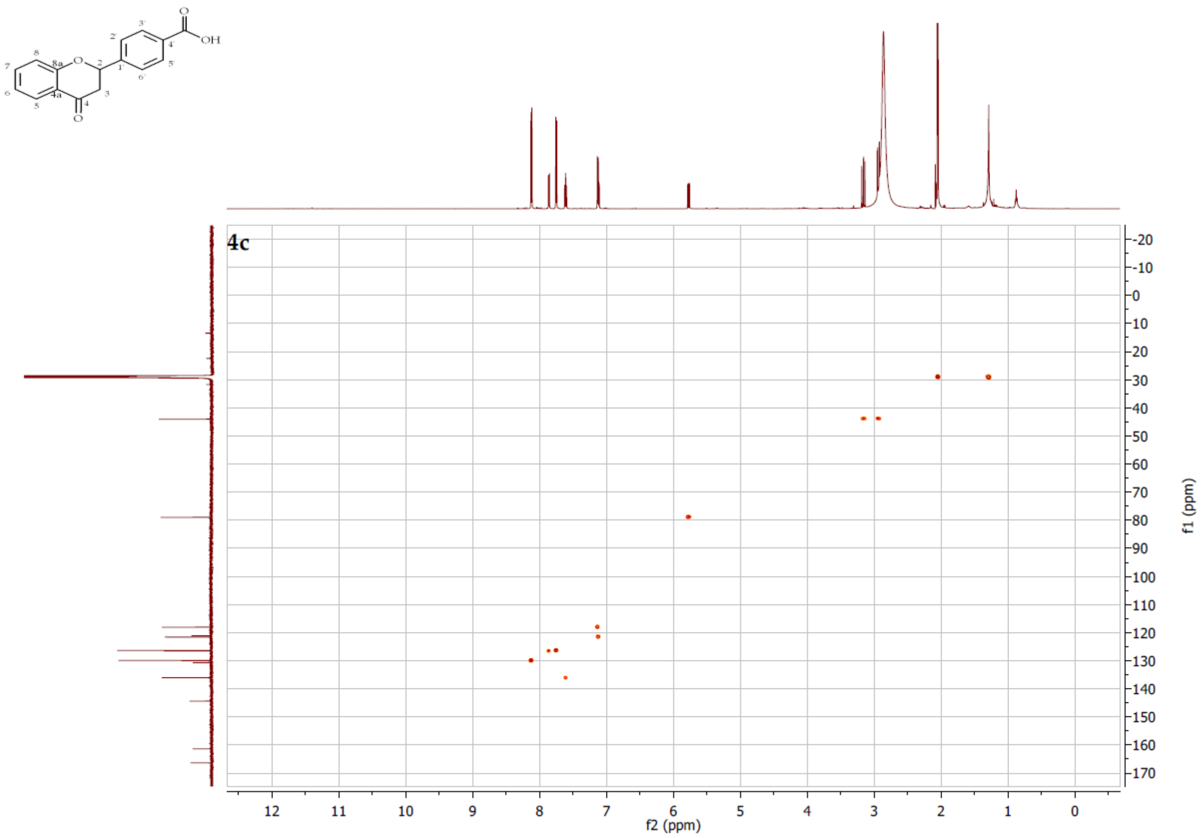

Figure S71. HSQC contour map – ^{1}H x ^{13}C of flavanone 4′-carboxylic acid (**4c**)

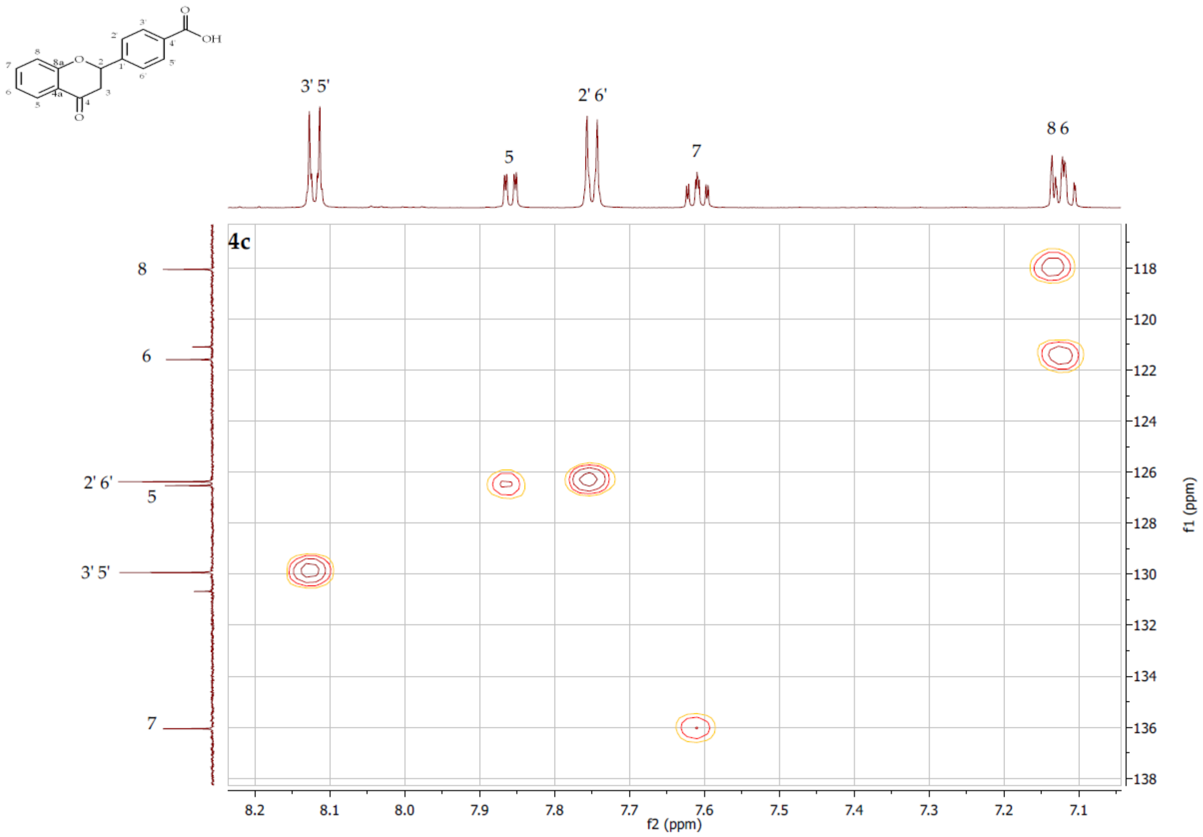

Figure S72. HSQC contour map – ^{1}H x ^{13}C expansion of flavanone 4′-carboxylic acid (**4c**)

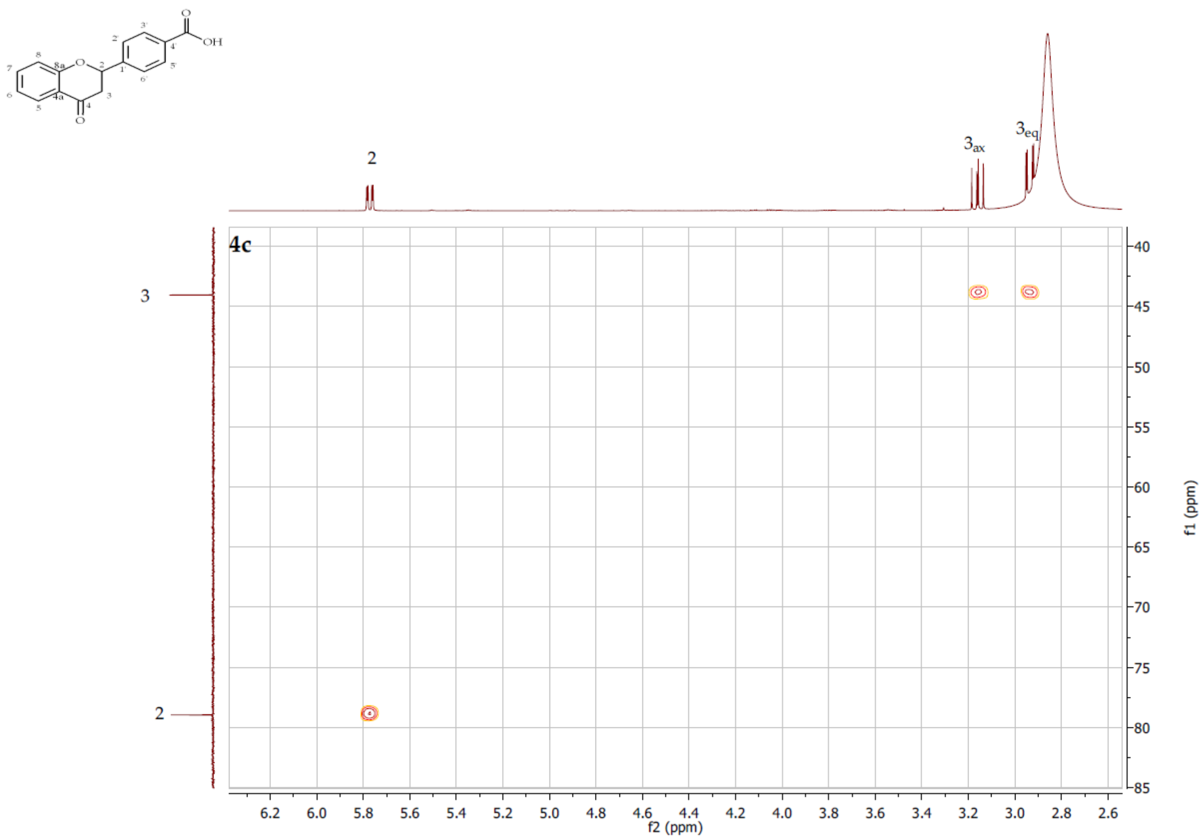

Figure S73. HSQC contour map – ^{1}H x ^{13}C expansion of flavanone 4′-carboxylic acid (**4c**)

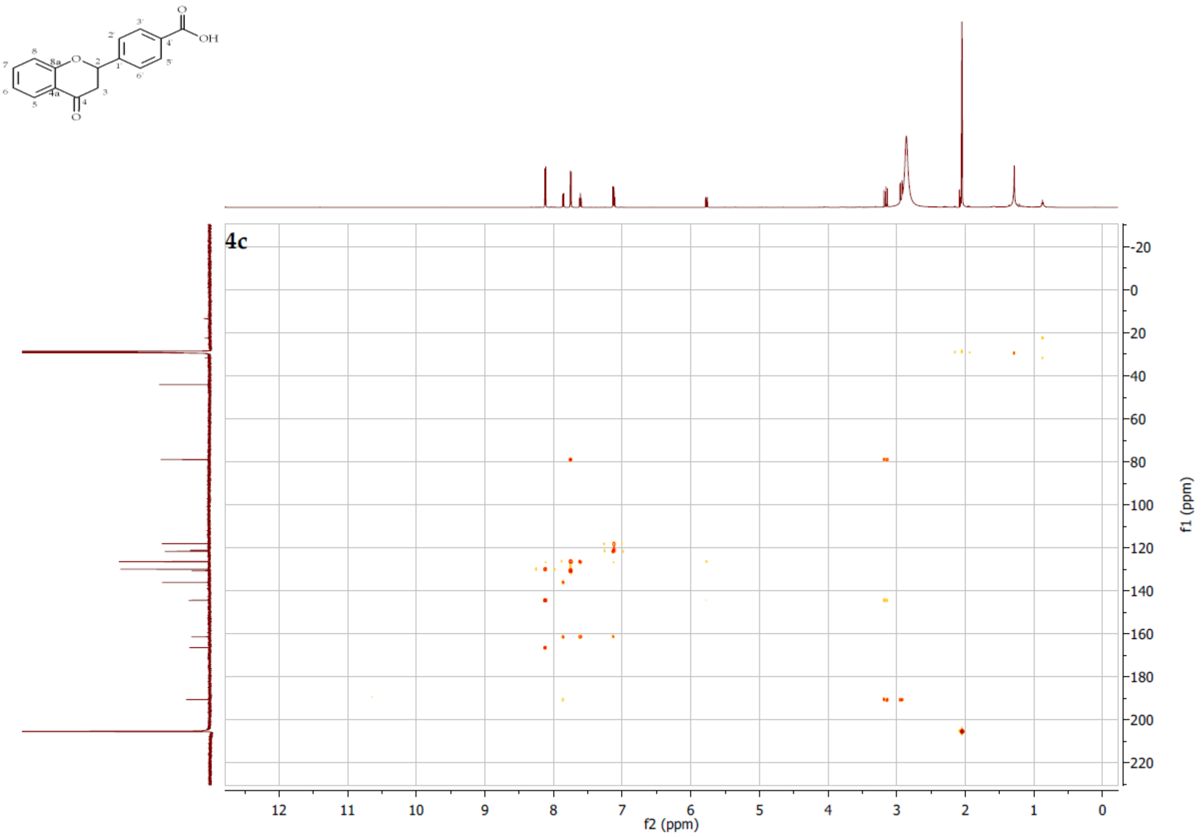

Figure S74. HMBC contour map – ^{1}H x ^{13}C of flavanone 4′-carboxylic acid (**4c**)

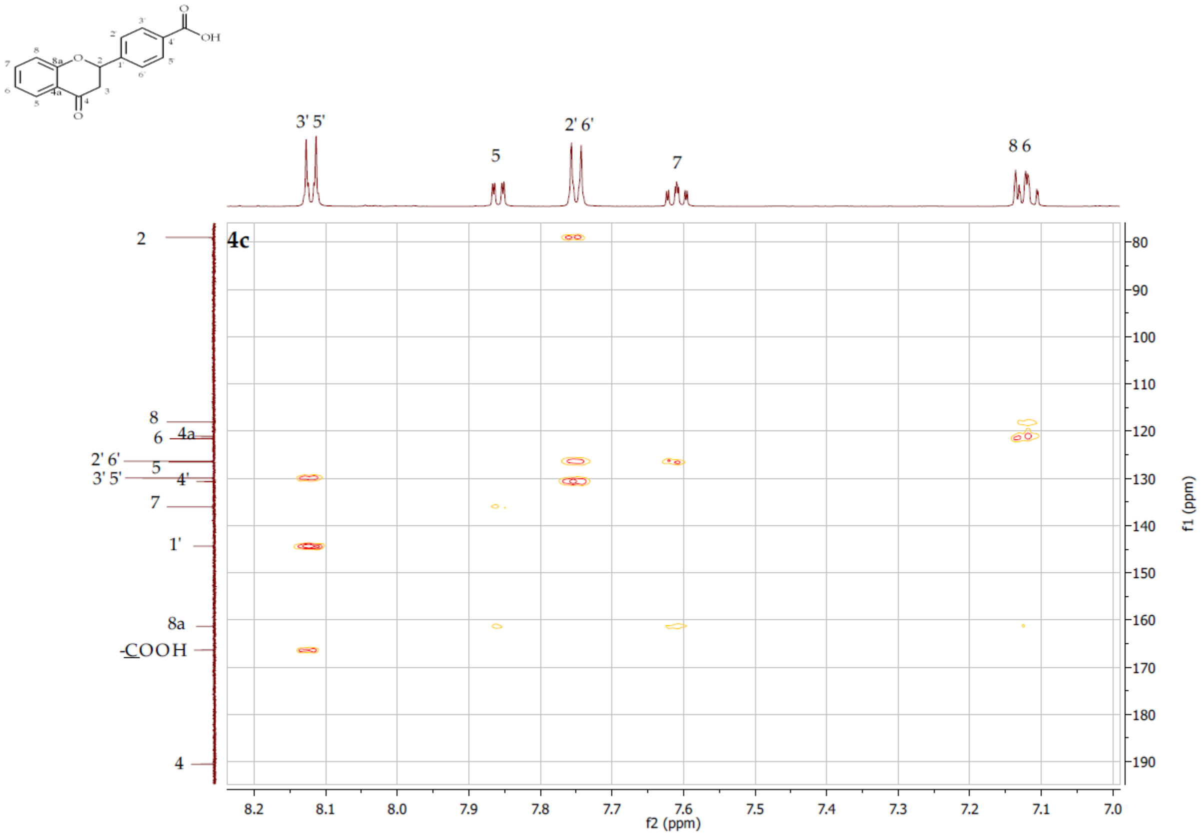

Figure S75. HMBC contour map – ^{1}H x ^{13}C expansion of flavanone 4′-carboxylic acid (**4c**)

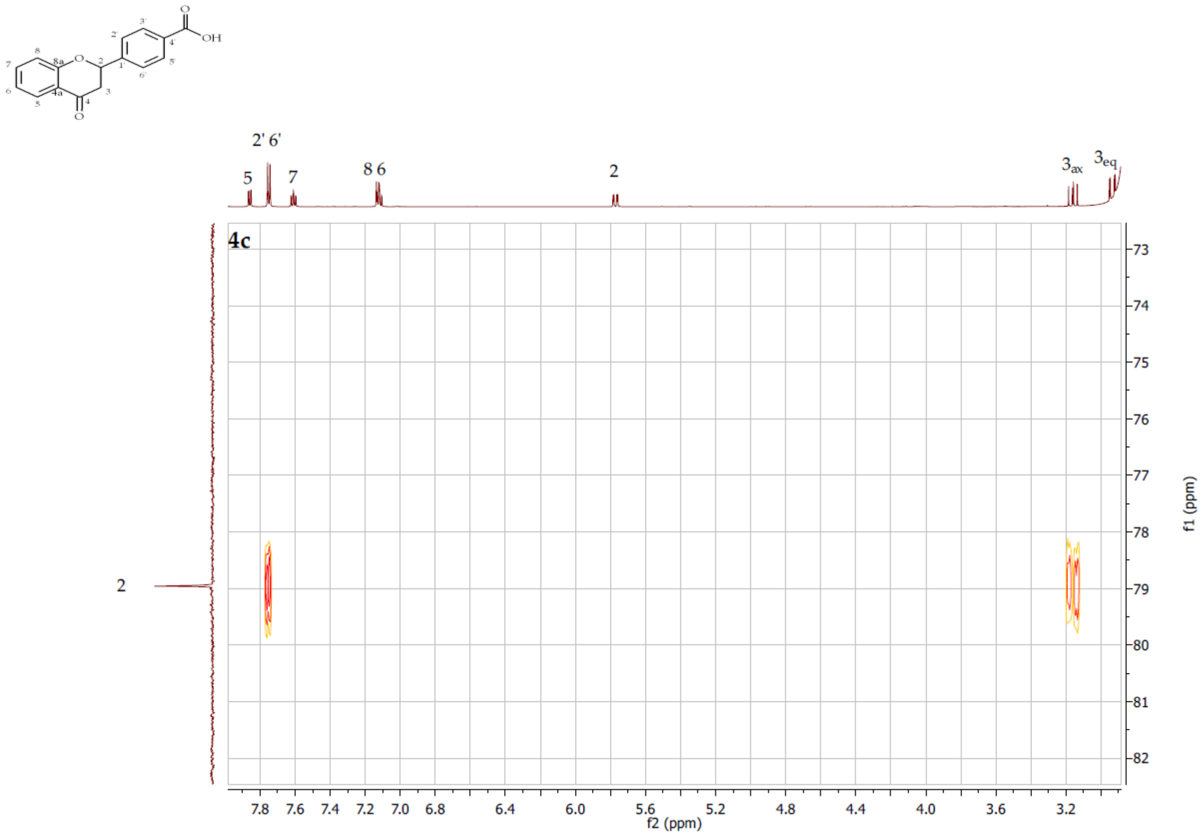

Figure S76. HMBC contour map – ^{1}H x ^{13}C expansion of flavanone 4′-carboxylic acid (**4c**)

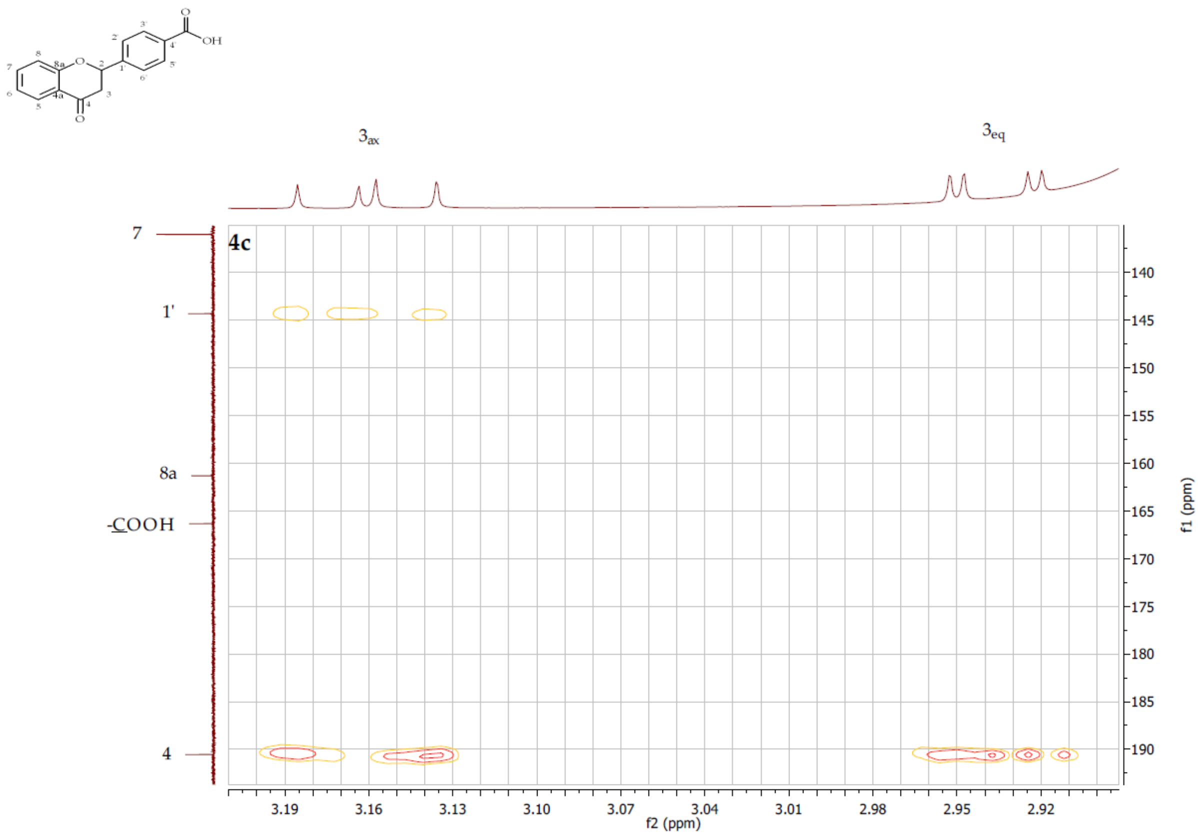

Figure S77. HMBC contour map – ^{1}H x ^{13}C expansion of flavanone 4′-carboxylic acid (**4c**)

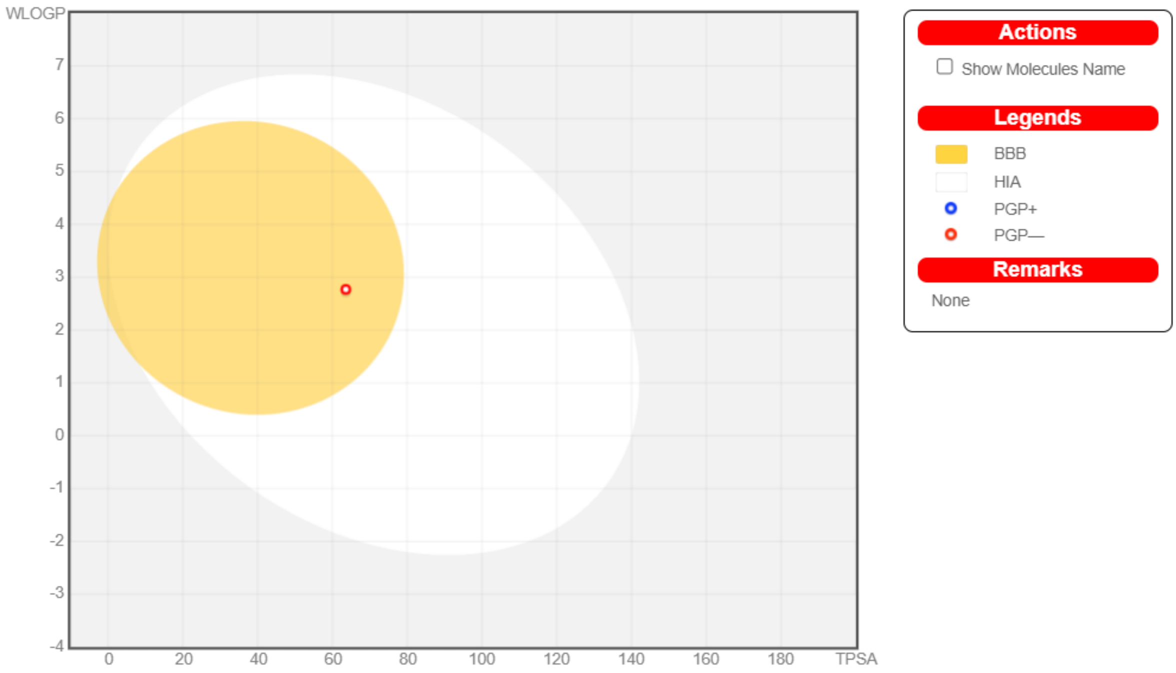

Molecule 1

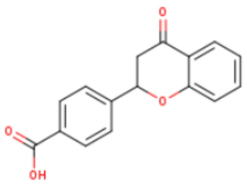

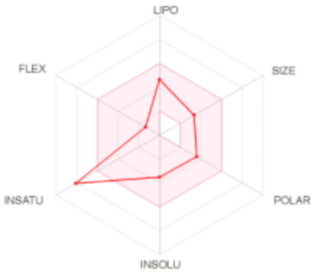

SMILES O=C1CC(Oc2c1cccc2)c1ccc(cc1)C(=O)O

Physicochemical Properties	
Formula	C16H12O4
Molecular weight	268.26 g/mol
Num. heavy atoms	20
Num. arom. heavy atoms	12
Fraction Csp3	0.12
Num. rotatable bonds	2
Num. H-bond acceptors	4
Num. H-bond donors	1
Molar Refractivity	72.46
TPSA	63.60 Å²
**Lipophilicity**	
Log $P_{o/w}$ (iLOGP)	2.03
Log $P_{o/w}$ (XLOGP3)	2.67
Log $P_{o/w}$ (WLOGP)	2.77
Log $P_{o/w}$ (MLOGP)	1.99
Log $P_{o/w}$ (SILICOS-IT)	2.93
Consensus Log $P_{o/w}$	2.48

Water Solubility	
Log *S* (ESOL)	-3.50
Solubility	8.54e-02 mg/ml ; 3.18e-04 mol/l
Class	Soluble
Log *S* (Ali)	-3.66
Solubility	5.90e-02 mg/ml ; 2.20e-04 mol/l
Class	Soluble
Log *S* (SILICOS-IT)	-4.52
Solubility	8.14e-03 mg/ml ; 3.03e-05 mol/l
Class	Moderately soluble
**Pharmacokinetics**	
GI absorption	High
BBB permeant	Yes
P-gp substrate	No
CYP1A2 inhibitor	No
CYP2C19 inhibitor	No
CYP2C9 inhibitor	No
CYP2D6 inhibitor	No
CYP3A4 inhibitor	No
Log K_p (skin permeation)	-6.04 cm/s
**Druglikeness**	
Lipinski	Yes; 0 violation
Ghose	Yes
Veber	Yes
Egan	Yes
Muegge	Yes
Bioavailability Score	0.85
**Medicinal Chemistry**	
PAINS	0 alert
Brenk	0 alert
Leadlikeness	Yes
Synthetic accessibility	2.88

Figure S78. Flavanone 4′-carboxylic acid (**4c**) physicochemical and ADME parameters prediction using the SwissADME modelling

Pa	Pi	Activity
0,912	0,008	Membrane integrity agonist
0,899	0,007	Methylenetetrahydrofolate reductase (NADPH) inhibitor
0,884	0,002	4-Nitrophenol 2-monooxygenase inhibitor
0,889	0,008	Testosterone 17beta-dehydrogenase (NADP+) inhibitor
0,865	0,004	Pullulanase inhibitor
0,858	0,003	Cholestanetriol 26-monooxygenase inhibitor
0,865	0,009	Chlordecone reductase inhibitor
0,835	0,003	Aryl-alcohol dehydrogenase (NADP+) inhibitor
0,830	0,004	3-Hydroxybenzoate 6-monooxygenase inhibitor
0,827	0,003	HMOX1 expression enhancer

Figure S79. Flavanone 4′-carboxylic acid (**4c**) biological activity prediction using the Way2Drug Pass online modelling

Name	Confidence	ChEMBL ID
Listeria monocytogenes	0.5021	CHEMBL614974
Yersinia pestis	0.3716	CHEMBL614597
Dialister micraerophilus	0.3680	CHEMBL615038
Dialister pneumosintes	0.3680	CHEMBL615039
Bacillus subtilis	0.3285	CHEMBL359
Dialister propionicifaciens	0.3217	CHEMBL615040
Parabacteroides merdae	0.3164	CHEMBL615057
Bacteroides uniformis	0.3124	CHEMBL612622
Pseudomonas fluorescens	0.3025	CHEMBL612500
RESISTANT Helicobacter pylori	0.2873	CHEMBL612600
RESISTANT Staphylococcus simulans	0.2849	CHEMBL612425
Clostridium ramosum	0.2730	CHEMBL614971

Figure S80. Flavanone 4′-carboxylic acid (**4c**) antibacterial activity prediction using the Way2Drug AntiBac-Pred modelling

Name	Confidence	ChEMBL ID
Candida dubliniensis	0.6520	CHEMBL613334
Epidermophyton floccosum	0.2644	CHEMBL612386
Absidia corymbifera	0.1102	CHEMBL612369
Rhizopus oryzae	0.1084	CHEMBL612306
Candida rugosa	0.0373	CHEMBL612869
Trichophyton mentagrophytes	0.0296	CHEMBL613162

Figure S81. Flavanone 4′-carboxylic acid (**4c**) antifungal activity prediction using the Way2Drug AntiFun-Pred modelling

Virus	Protein target	Confidence
Human immunodeficiency virus 2	Human immunodeficiency virus type 2 integrase	0.7289
Dengue virus type 2	Genome polyprotein	0.4804
Severe acute respiratory syndrome coronavirus 2	Replicase polyprotein 1ab	0.2878
Vaccinia virus (strain Western Reserve) (VACV) (Vaccinia virus (strainWR))	DNA polymerase	0.0949
Influenza A virus	Neuraminidase	0.0880
SARS coronavirus	Replicase polyprotein 1ab	0.0834
Middle East respiratory syndrome-related coronavirus (isolate UnitedKingdom/H123990006/2012) (Betacoronavirus England 1) (Humancoronavirus EMC)	Replicase polyprotein 1ab	0.0481

Figure S82. Flavanone 4′-carboxylic acid (**4c**) antiviral activity prediction using the Way2Drug AntiVir-Pred modelling

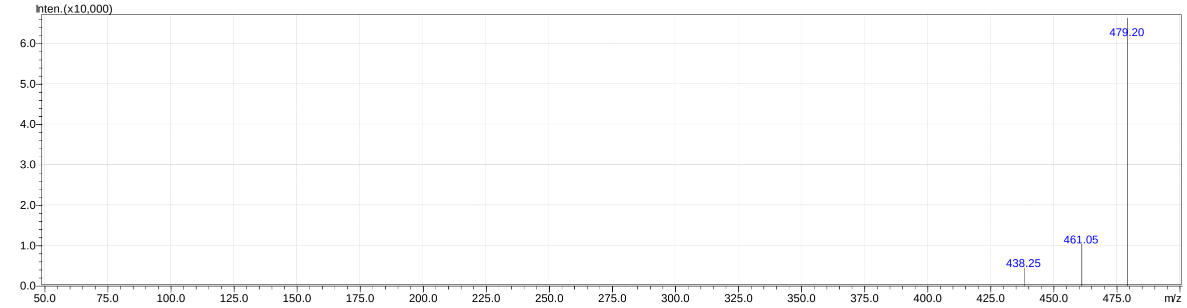

Figure S83. MS analysis of and 4′-hydroxymethylflavanone 4-*O*-*β*-D-(4′′-*O*-methyl)-glucopyranoside (**4d**)

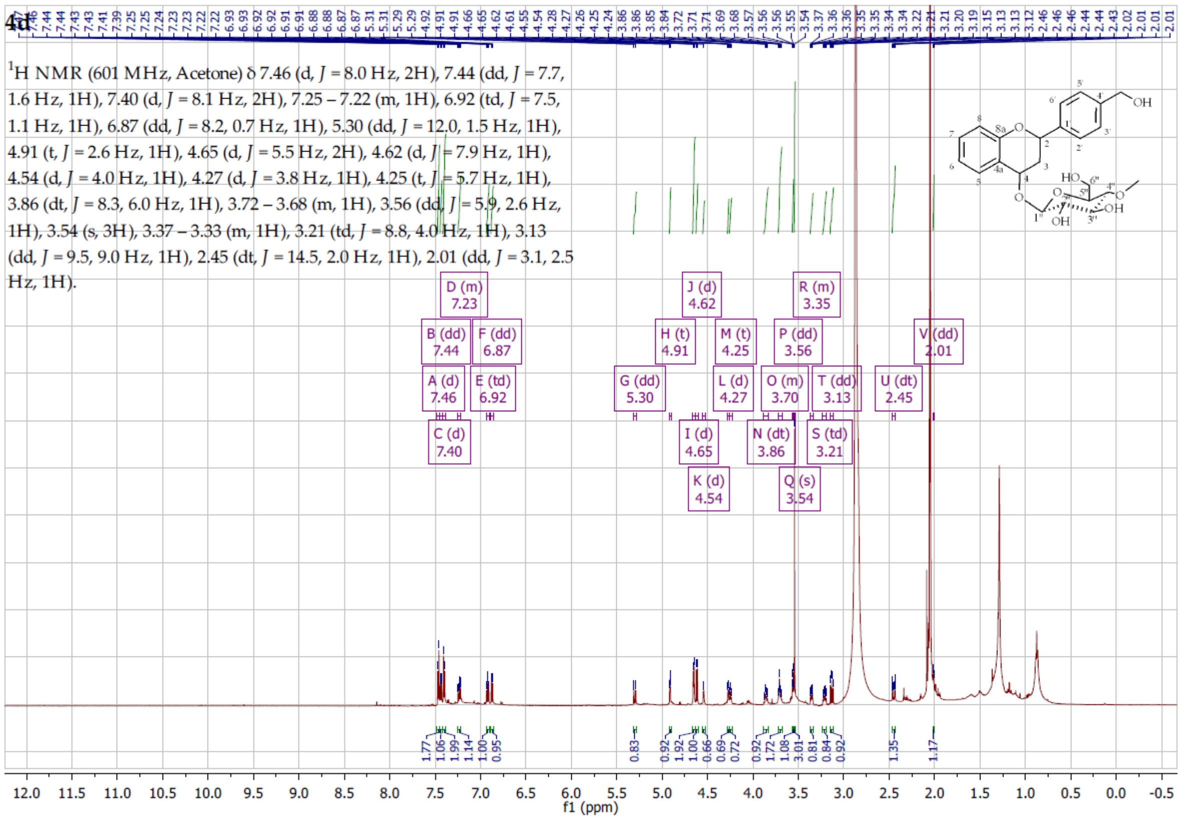

Figure S84. ^{1}H NMR spectrum (*δ*, acetone-d6, 600 MHz) of 4′-hydroxymethylflavanone 4-*O*-*β*-D-(4′′-*O*-methyl)-glucopyranoside (**4d**)

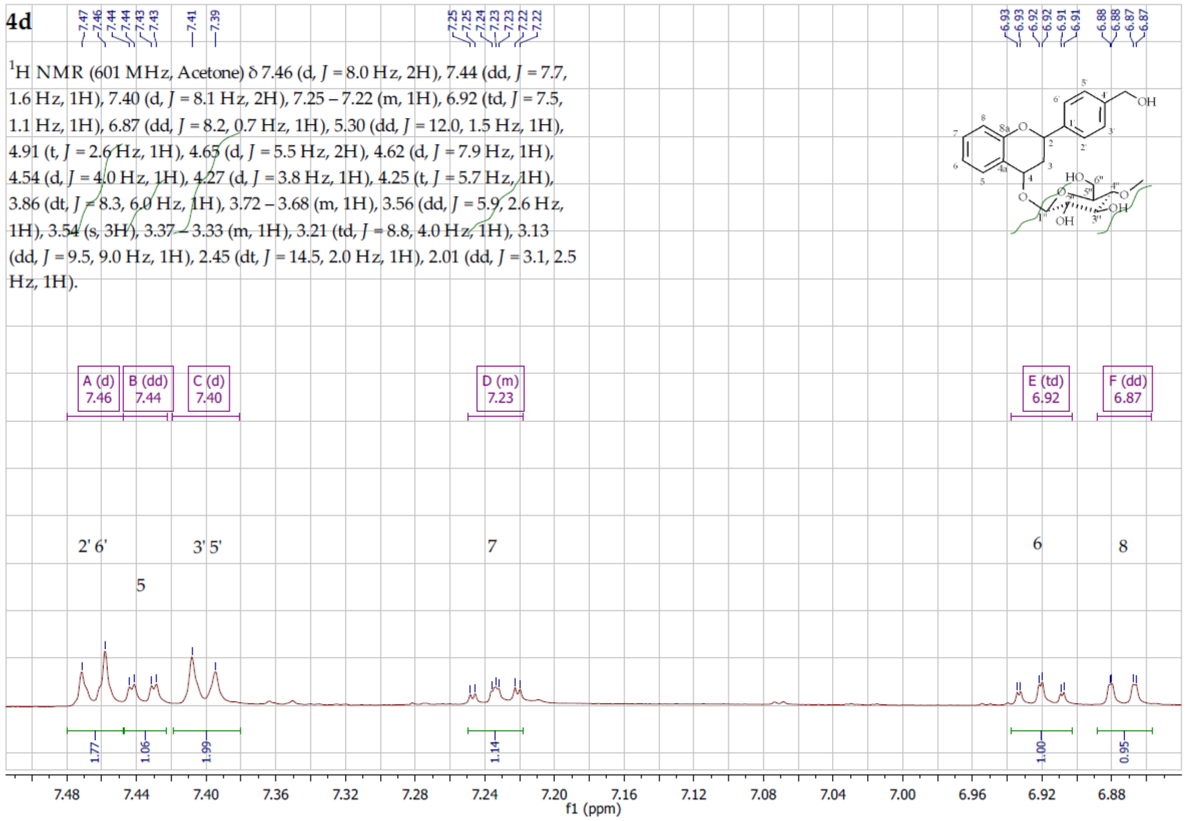

Figure S85. ^{1}H NMR spectrum expansion (*δ*, acetone-d6, 600 MHz) of 4′-hydroxymethylflavanone 4-*O*-*β*-D-(4′′-*O*-methyl)-glucopyranoside (**4d**)

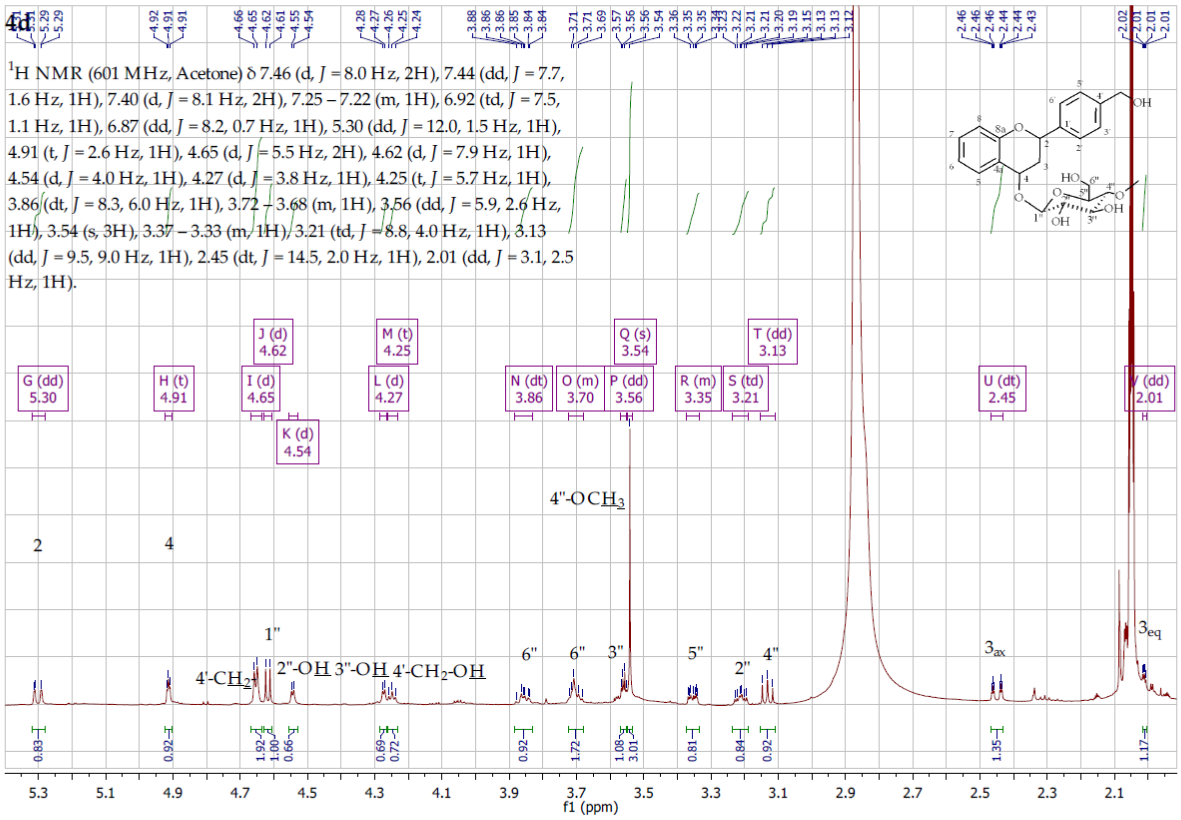

Figure S86. ^{1}H NMR spectrum expansion (*δ*, acetone-d6, 600 MHz) of 4′-hydroxymethylflavanone 4-*O*-*β*-D-(4′′-*O*-methyl)-glucopyranoside (**4d**)

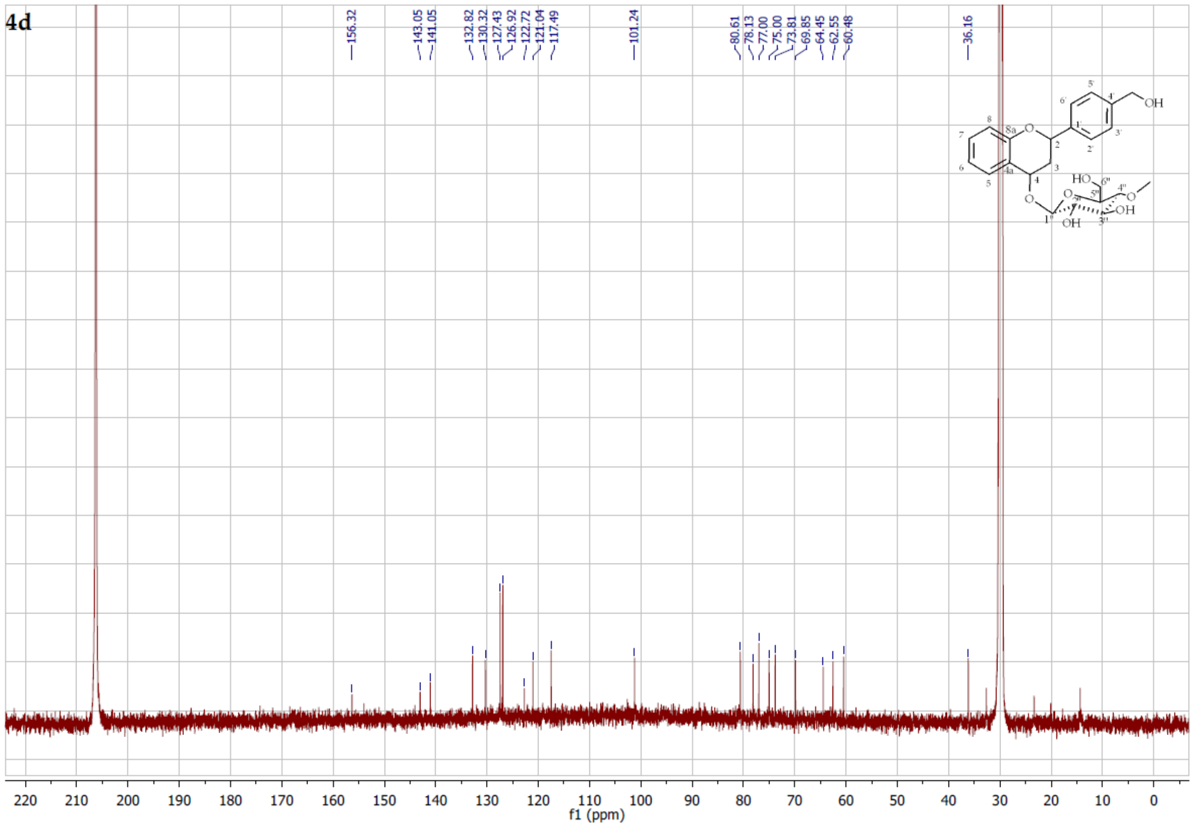

Figure S87. ^{13}C NMR spectrum (*δ*, acetone-d6, 151 MHz) of 4′-hydroxymethylflavanone 4-*O*-*β*-D-(4′′-*O*-methyl)-glucopyranoside (**4d**)

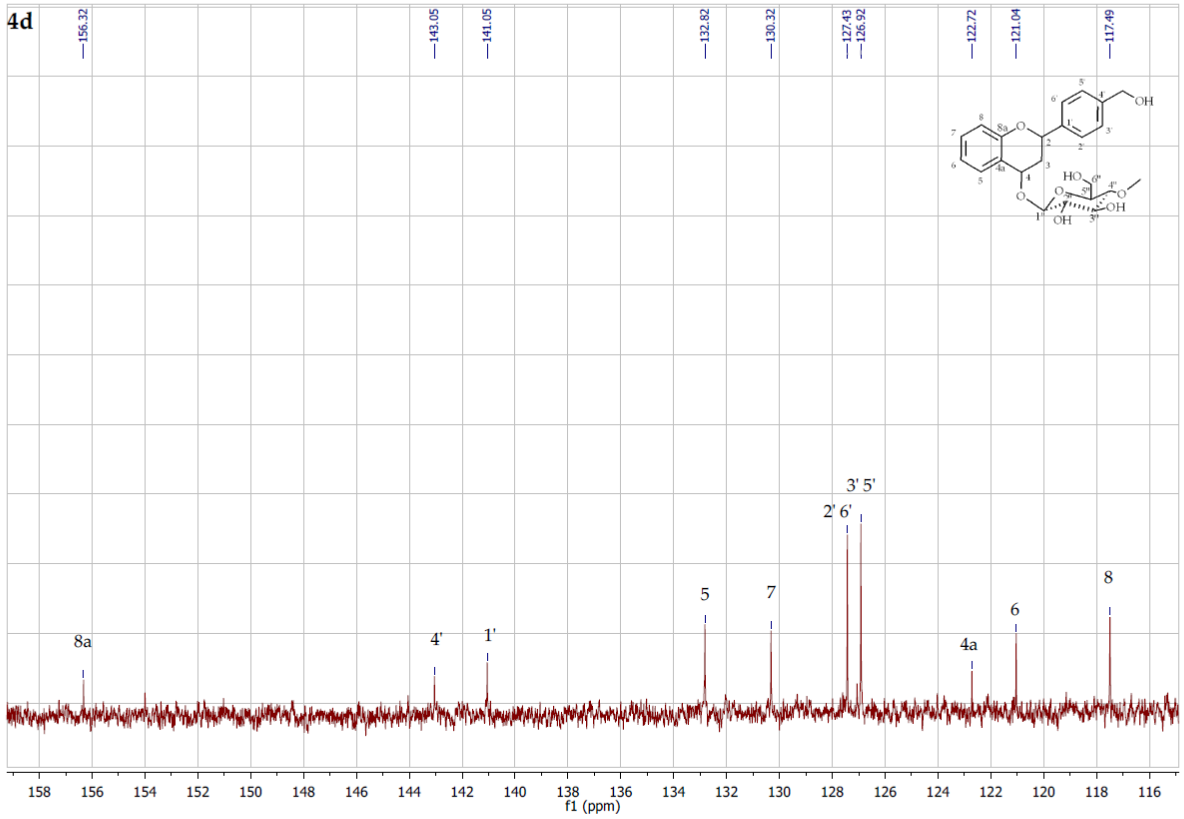

Figure S88. ^{13}C NMR spectrum expansion (*δ*, acetone-d6, 151 MHz) of 4′-hydroxymethylflavanone 4-*O*-*β*-D-(4′′-*O*-methyl)-glucopyranoside (**4d**)

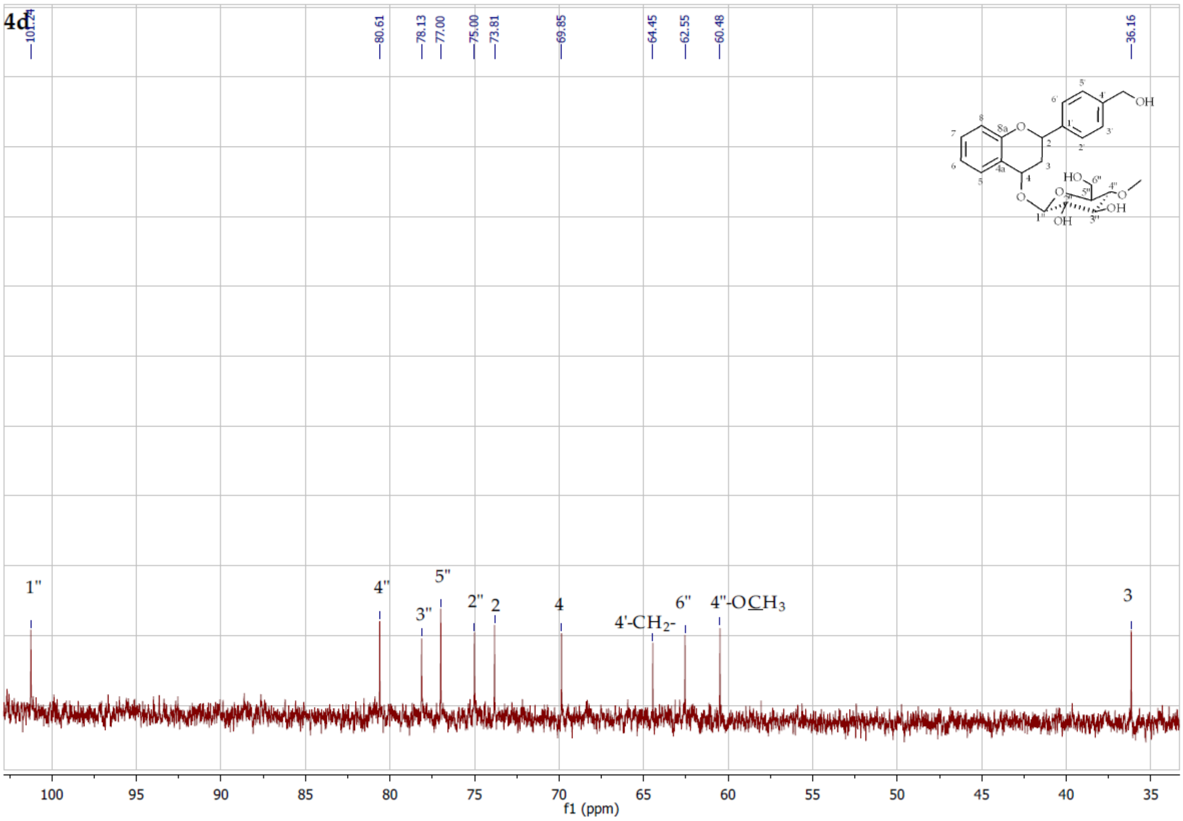

Figure S89. ^{13}C NMR spectrum expansion (*δ*, acetone-d6, 151 MHz) of 4′-hydroxymethylflavanone 4-*O*-*β*-D-(4′′-*O*-methyl)-glucopyranoside (**4d**)

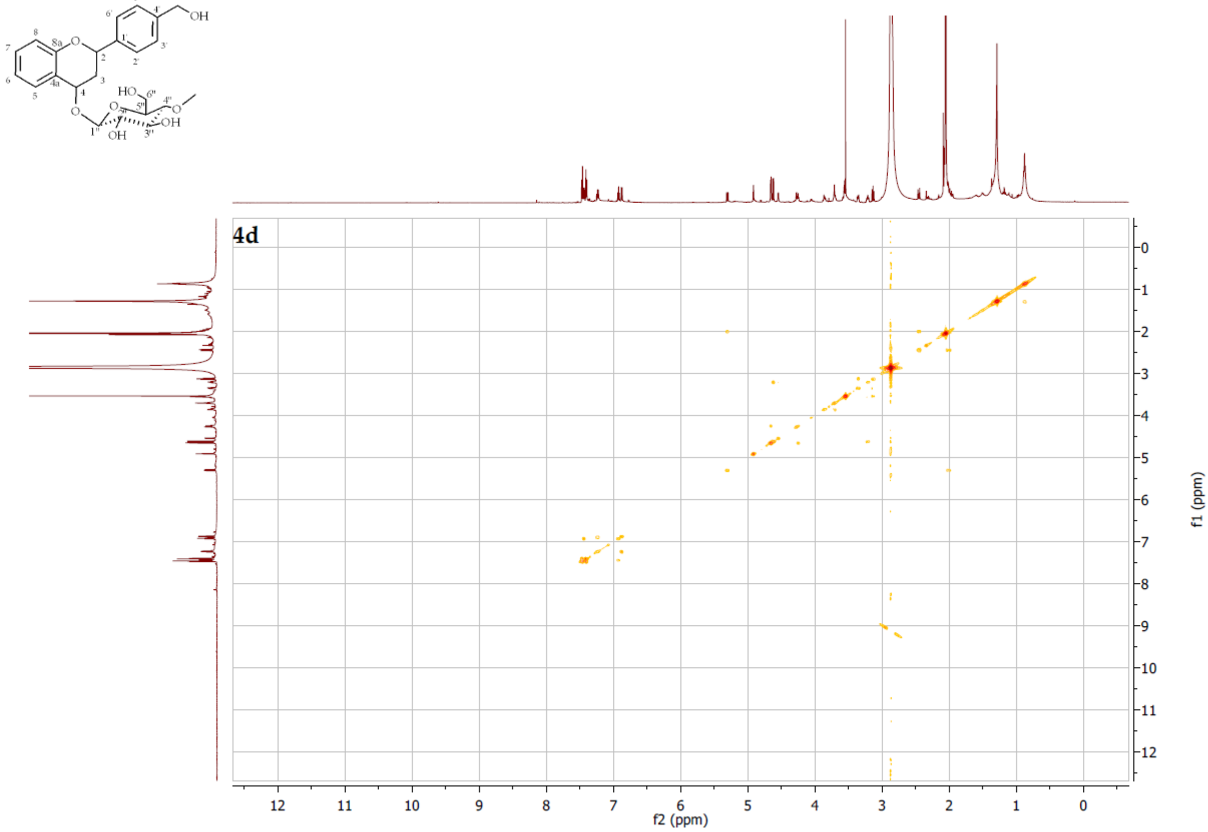

Figure S90. COSY contour map – ^{1}H x ^{1}H of 4′-hydroxymethylflavanone 4-*O*-*β*-D-(4′′-*O*-methyl)-glucopyranoside (**4d**)

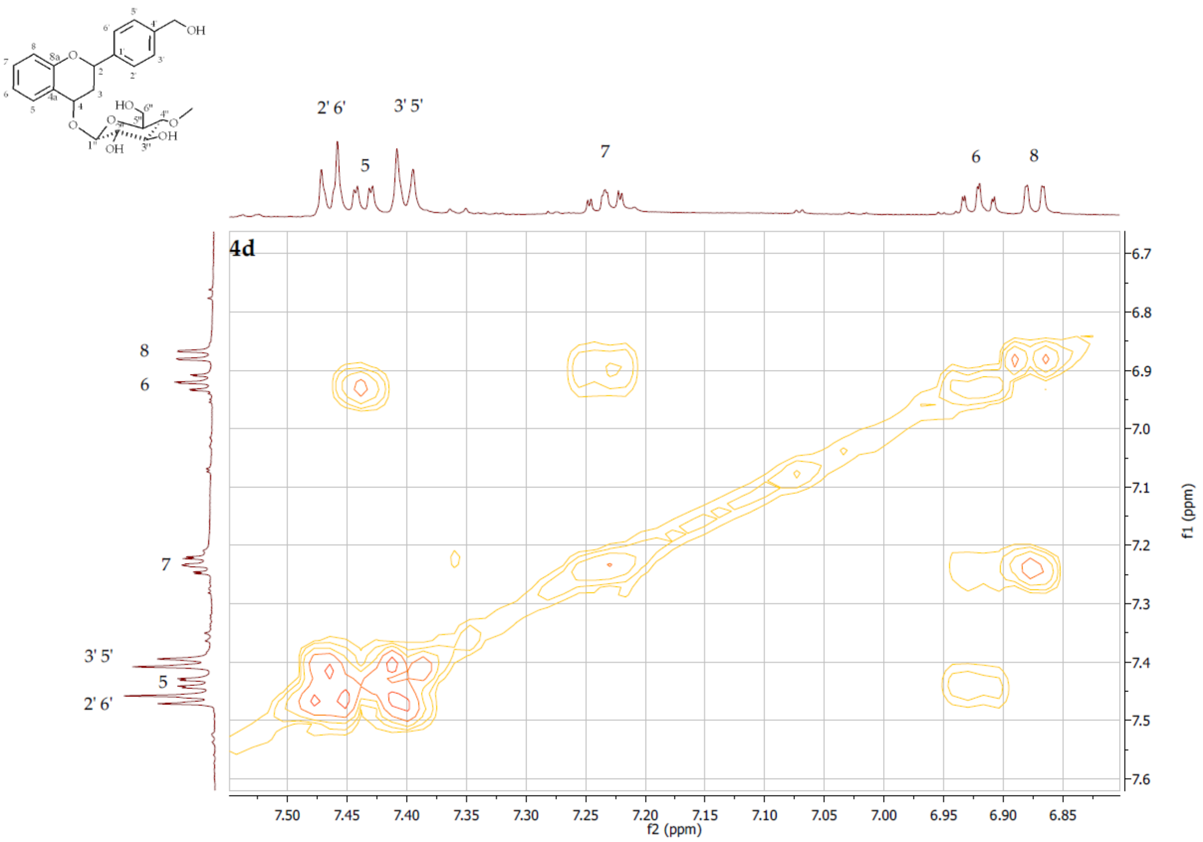

Figure S91. COSY contour map – ^{1}H x ^{1}H expansion of 4′-hydroxymethylflavanone 4-*O*-*β*-D-(4′′-*O*-methyl)-glucopyranoside (**4d**)

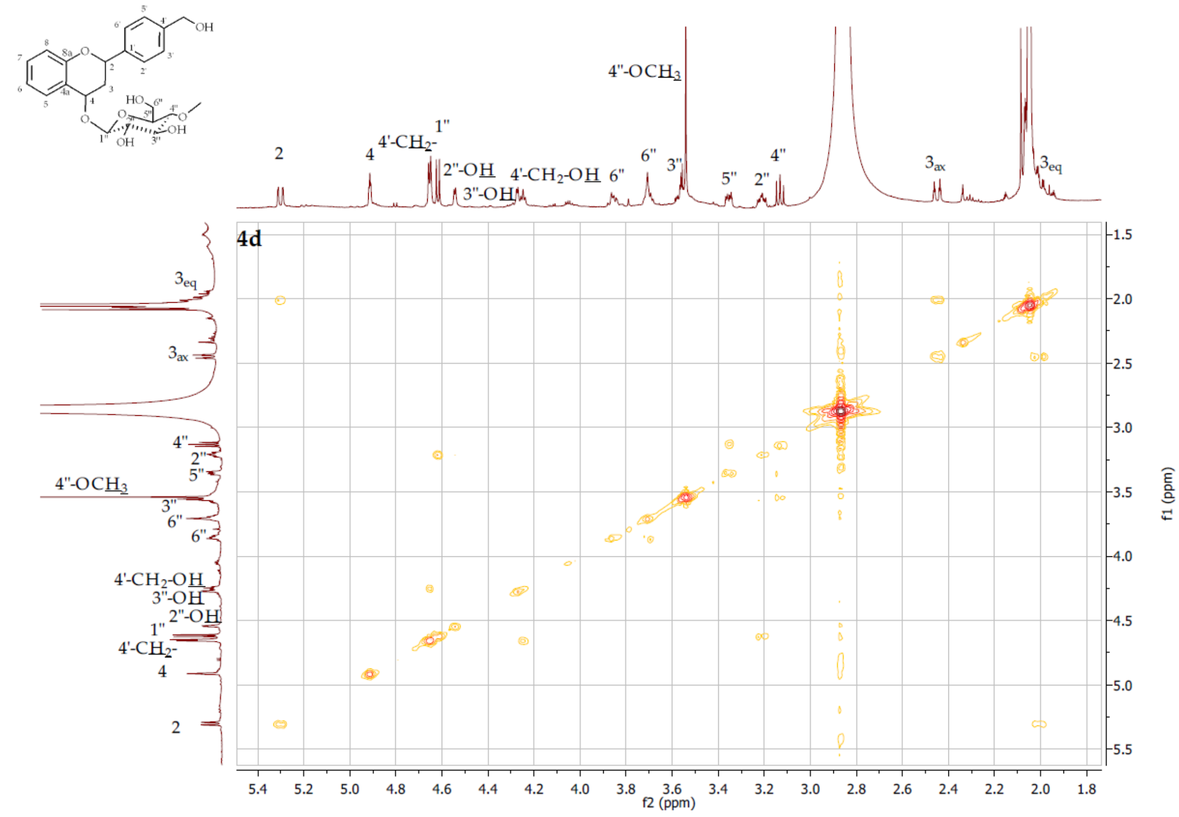

Figure S92. COSY contour map – ^{1}H x ^{1}H expansion of 4′-hydroxymethylflavanone 4-*O*-*β*-D-(4′′-*O*-methyl)-glucopyranoside (**4d**)

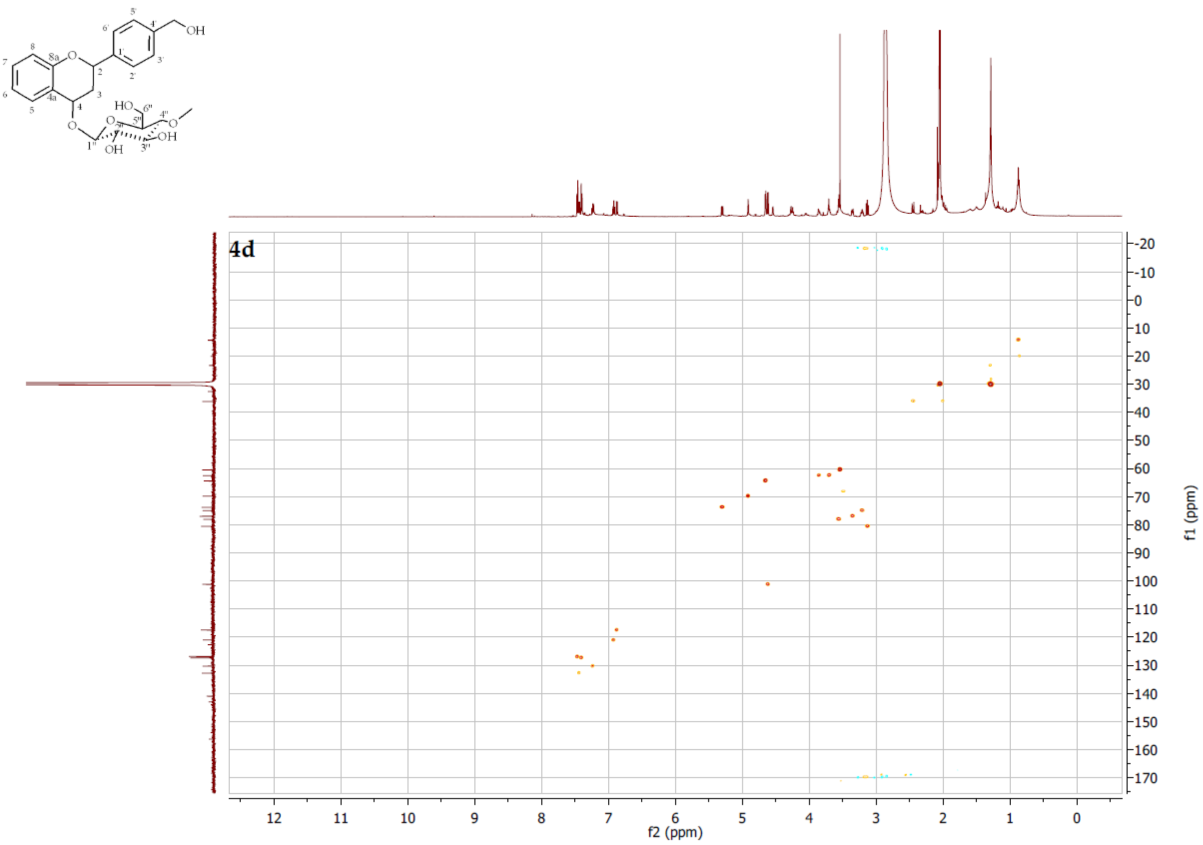

Figure S93. HSQC contour map – ^{1}H x ^{13}C of 4′-hydroxymethylflavanone 4-*O*-*β*-D-(4′′-*O*-methyl)-glucopyranoside (**4d**)

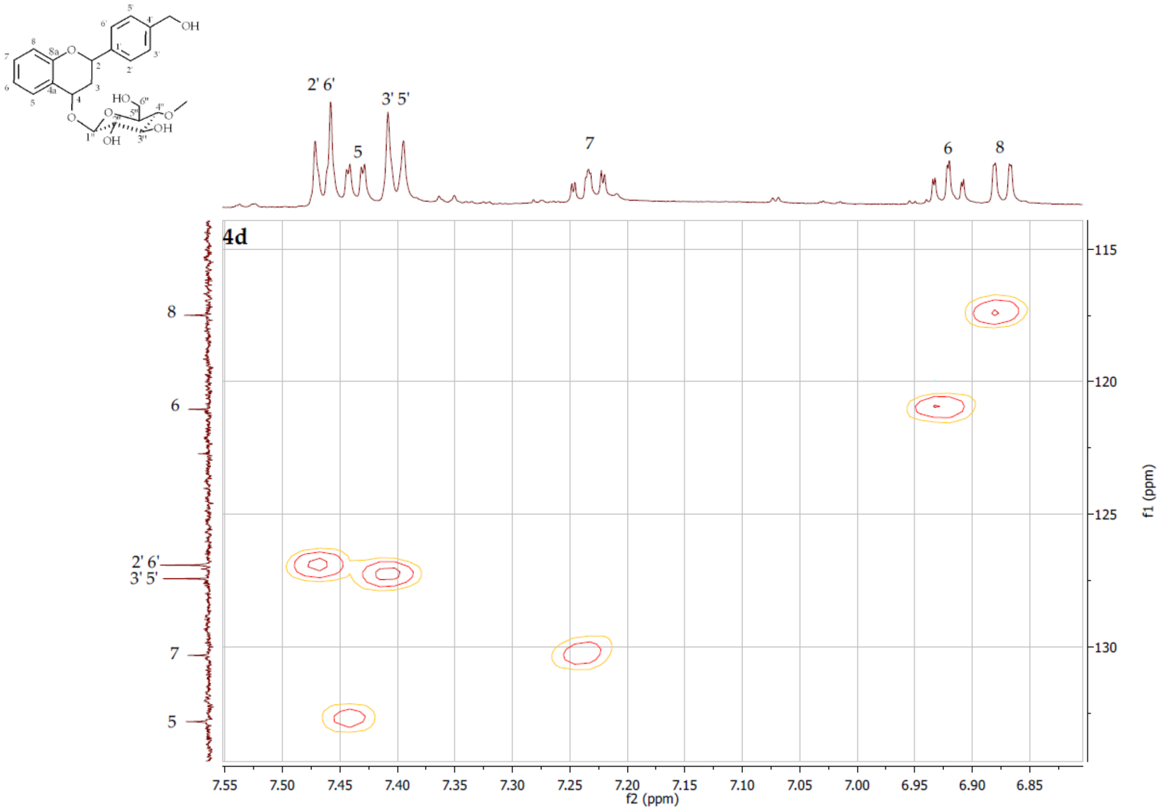

Figure S94. HSQC contour map – ^{1}H x ^{13}C expansion of 4′-hydroxymethylflavanone 4-*O*-*β*-D-(4′′-*O*-methyl)-glucopyranoside (**4d**)

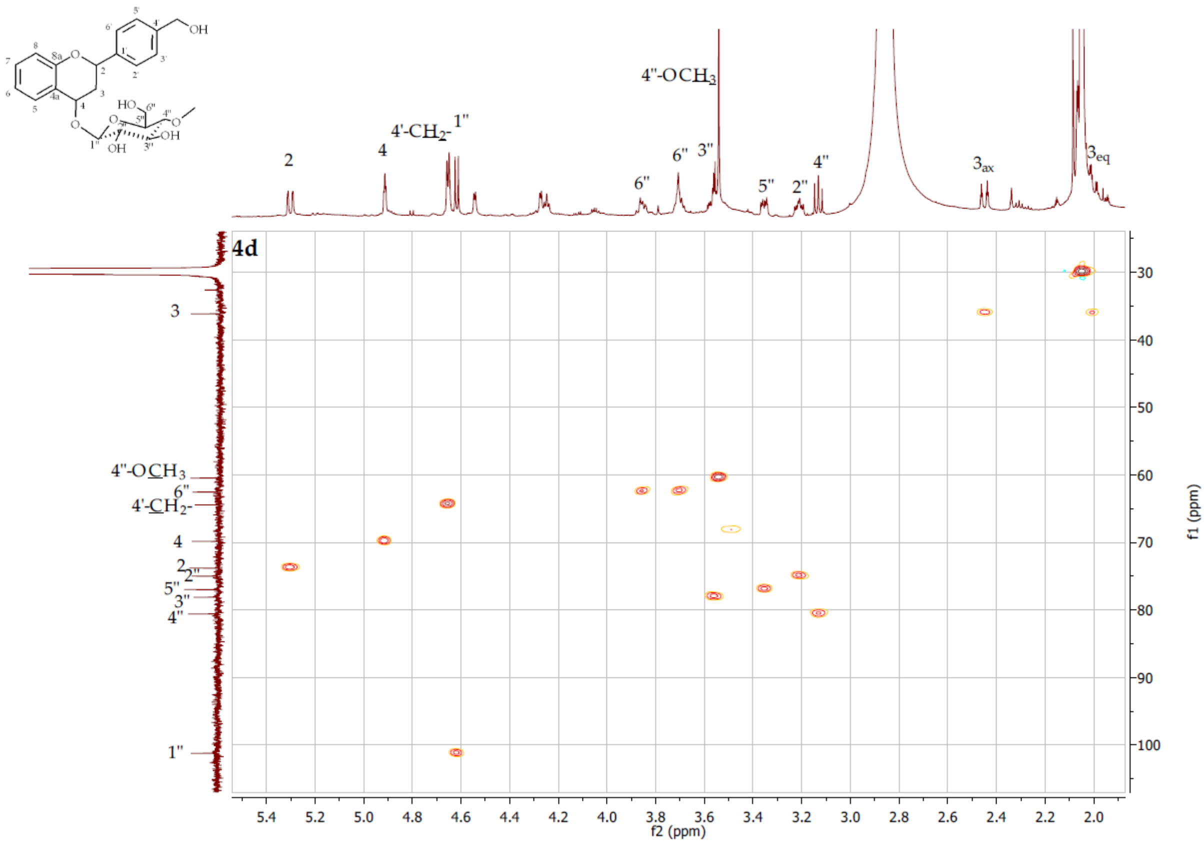

Figure S95. HSQC contour map – ^{1}H x ^{13}C expansion of 4′-hydroxymethylflavanone 4-*O*-*β*-D-(4′′-*O*-methyl)-glucopyranoside (**4d**)

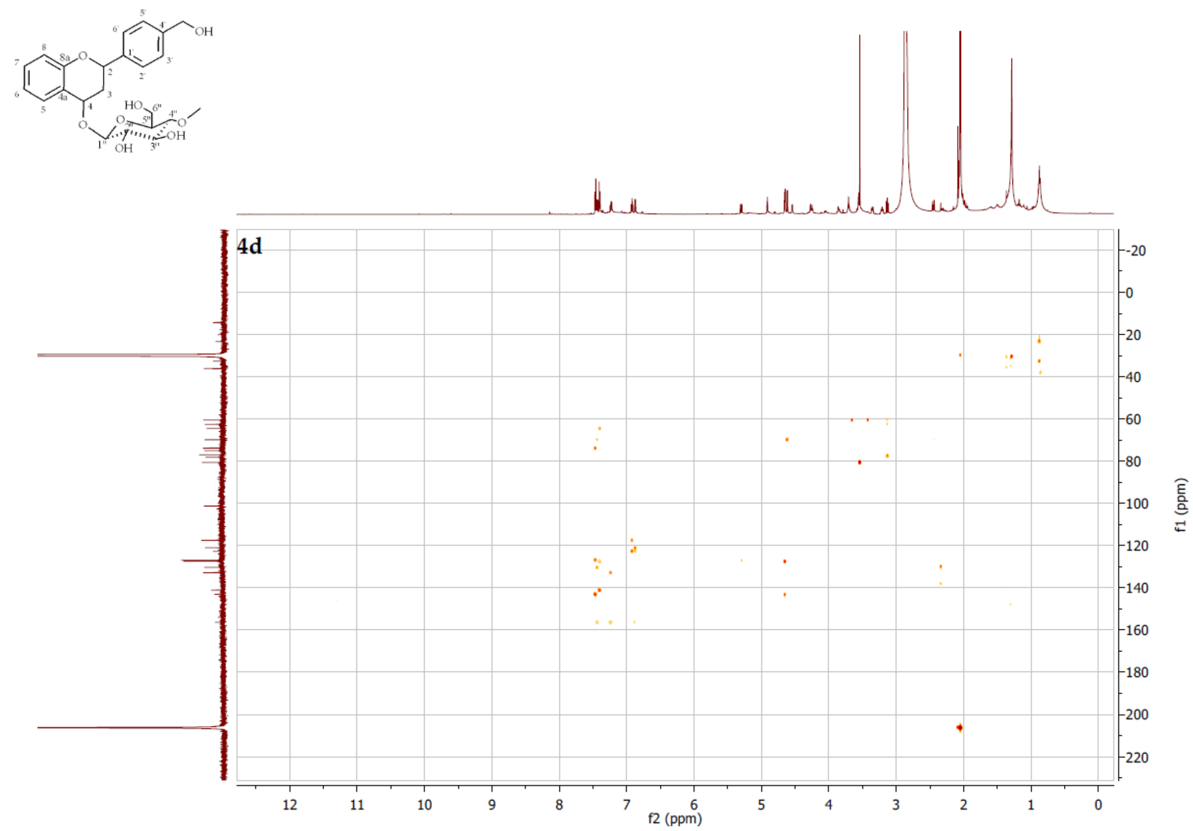

Figure S96. HMBC contour map – ^{1}H x ^{13}C of 4′-hydroxymethylflavanone 4-*O*-*β*-D-(4′′-*O*-methyl)-glucopyranoside (**4d**)

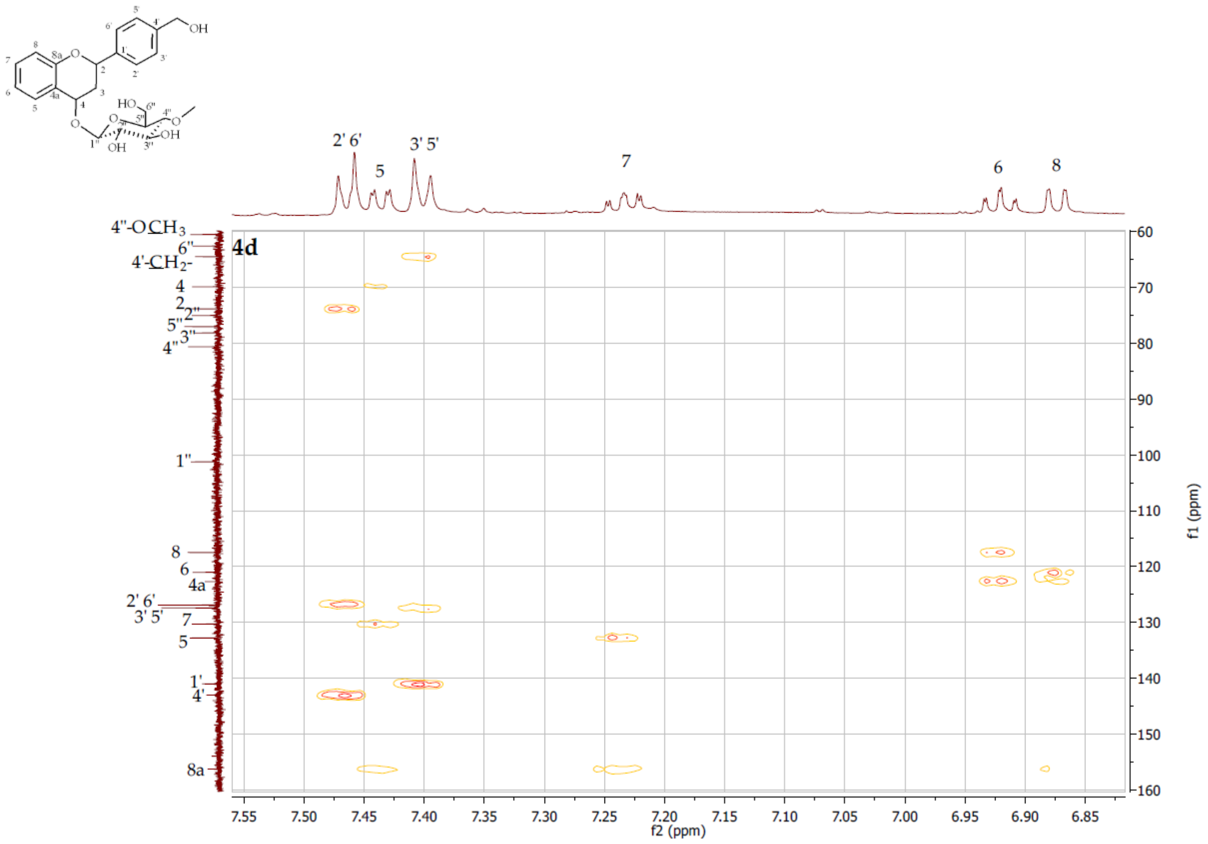

Figure S97. HMBC contour map – $^{1}H \times ^{13}C$ expansion of 4′-hydroxymethylflavanone 4-*O*-*β*-D-(4′′-*O*-methyl)-glucopyranoside (**4d**)

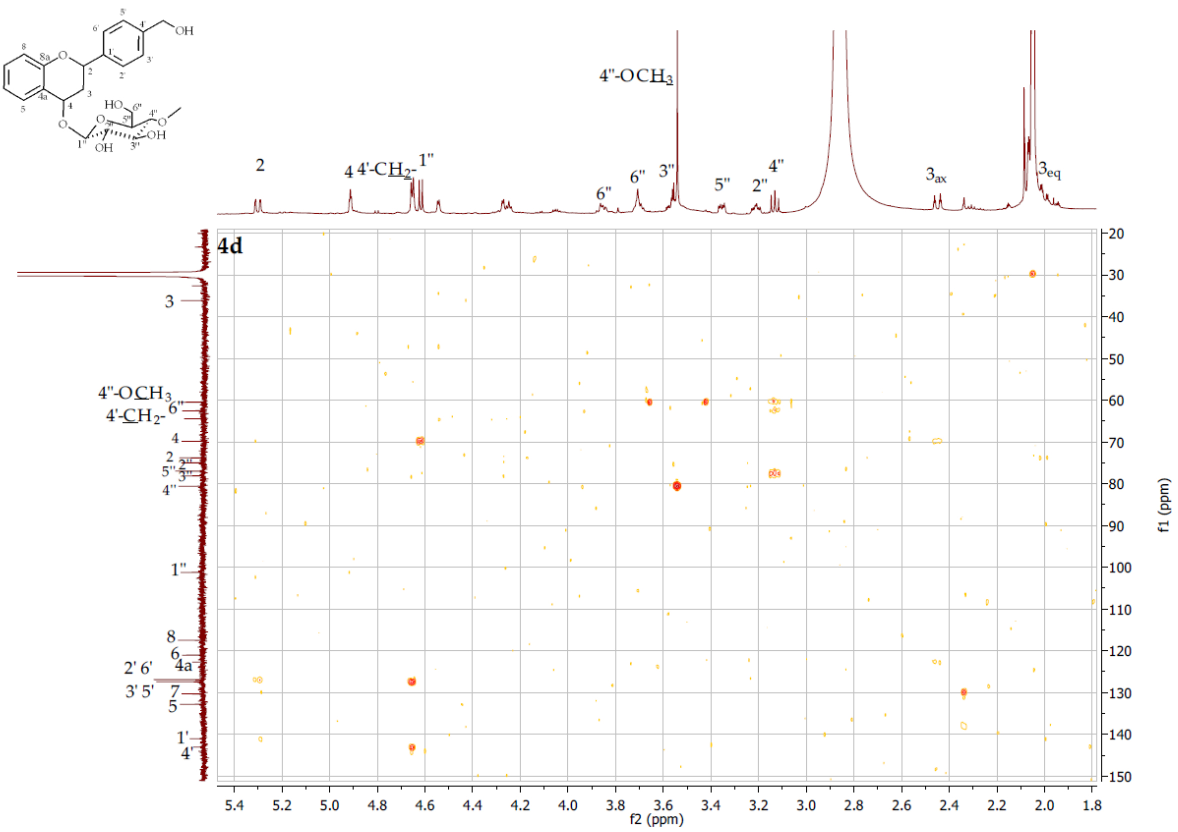

Figure S98. HMBC contour map – $^{1}H \times ^{13}C$ expansion of 4′-hydroxymethylflavanone 4-*O*-*β*-D-(4′′-*O*-methyl)-glucopyranoside (**4d**)

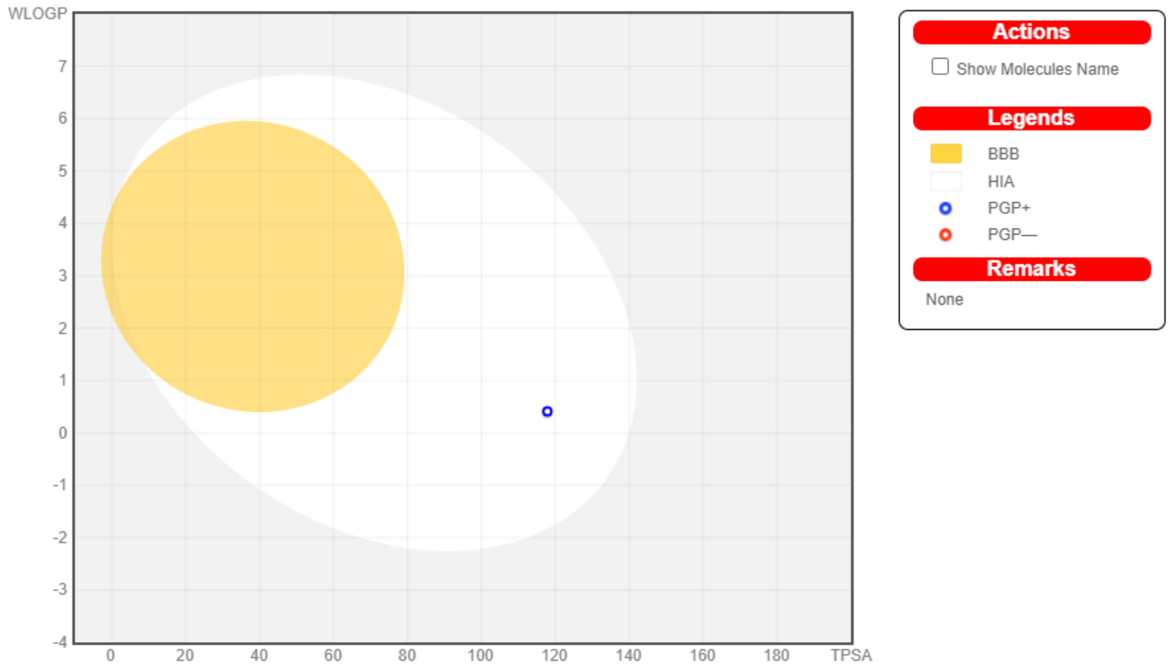

Molecule 1

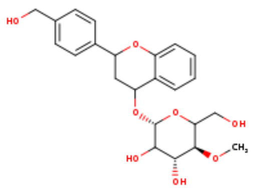

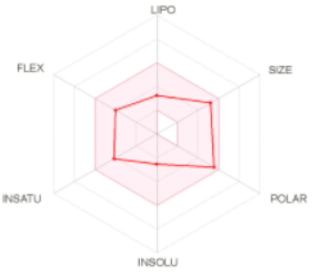

SMILES OCc1ccc(cc1)C1CC(O[C@@H]2OC(CO)[C@H]([C@@H](C2O)O)OC)c2c(O1)cccc2

Physicochemical Properties	
Formula	C23H28O8
Molecular weight	432.46 g/mol
Num. heavy atoms	31
Num. arom. heavy atoms	12
Fraction Csp3	0.48
Num. rotatable bonds	6
Num. H-bond acceptors	8
Num. H-bond donors	4
Molar Refractivity	109.48
TPSA	117.84 Å²

Lipophilicity	
Log $P_{o/w}$ (iLOGP)	2.24
Log $P_{o/w}$ (XLOGP3)	0.21
Log $P_{o/w}$ (WLOGP)	0.41
Log $P_{o/w}$ (MLOGP)	-0.16
Log $P_{o/w}$ (SILICOS-IT)	1.18
Consensus Log $P_{o/w}$	0.78

Water Solubility	
Log *S* (ESOL)	-2.54
Solubility	1.24e+00 mg/ml ; 2.86e-03 mol/l
Class	Soluble
Log *S* (Ali)	-2.24
Solubility	2.47e+00 mg/ml ; 5.70e-03 mol/l
Class	Soluble
Log *S* (SILICOS-IT)	-3.27
Solubility	2.32e-01 mg/ml ; 5.37e-04 mol/l
Class	Soluble

Pharmacokinetics	
GI absorption	High
BBB permeant	No
P-gp substrate	Yes
CYP1A2 inhibitor	No
CYP2C19 inhibitor	No
CYP2C9 inhibitor	No
CYP2D6 inhibitor	No
CYP3A4 inhibitor	No
Log K_p (skin permeation)	-8.79 cm/s

Druglikeness	
Lipinski	Yes; 0 violation
Ghose	Yes
Veber	Yes
Egan	Yes
Muegge	Yes
Bioavailability Score	0.55

Medicinal Chemistry	
PAINS	0 alert
Brenk	0 alert
Leadlikeness	No; 1 violation: MW>350
Synthetic accessibility	5.24

Figure S99. 4′-Hydroxymethylflavanone 4-*O*-*β*-D-(4′′-*O*-methyl)-glucopyranoside (**4d**) physicochemical and ADME parameters prediction using the SwissADME modelling

Pa	Pi	Activity
0,926	0,006	CDP-glycerol glycerophosphotransferase inhibitor
0,881	0,003	Anticarcinogenic
0,890	0,014	Membrane integrity agonist
0,846	0,004	Membrane integrity antagonist
0,844	0,011	Benzoate-CoA ligase inhibitor
0,838	0,008	Antineoplastic
0,833	0,013	CYP2H substrate
0,820	0,004	Hepatoprotectant
0,827	0,012	Sugar-phosphatase inhibitor
0,817	0,005	Caspase 3 stimulant

Figure S100. 4′-Hydroxymethylflavanone 4-*O*-*β*-D-(4′′-*O*-methyl)-glucopyranoside (**4d**) biological activity prediction using the Way2Drug Pass online modelling

Name	Confidence	ChEMBL ID
Clostridium ramosum	0.6571	CHEMBL614971
RESISTANT Acinetobacter pittii	0.6183	CHEMBL3140321
Mycobacterium mageritense	0.6065	CHEMBL612959
Actinomyces meyeri	0.6048	CHEMBL612289
RESISTANT Mycobacterium ulcerans	0.5940	CHEMBL612965
Clostridium cadaveris	0.5759	CHEMBL614970
Streptococcus oralis	0.4544	CHEMBL613305
Staphylococcus lugdunensis	0.4471	CHEMBL613303
Nocardia transvalensis	0.4462	CHEMBL613234
Yersinia pestis	0.4460	CHEMBL614597
RESISTANT Staphylococcus aureus subsp. aureus RN4220	0.4451	CHEMBL2366906
Lactobacillus plantarum	0.4440	CHEMBL614973
Clostridium sordellii	0.4165	CHEMBL613072
Listeria monocytogenes	0.4146	CHEMBL614974

Figure S101. 4′-Hydroxymethylflavanone 4-*O*-*β*-D-(4′′-*O*-methyl)-glucopyranoside (**4d**) antibacterial activity prediction using the Way2Drug AntiBac-Pred modelling

Name	Confidence	ChEMBL ID
Rhizopus oryzae	0.6079	CHEMBL612306
Candida dubliniensis	0.5754	CHEMBL613334
Absidia corymbifera	0.5538	CHEMBL612369
Trichophyton mentagrophytes	0.4124	CHEMBL613162
Mucor	0.3136	CHEMBL612521
Epidermophyton floccosum	0.3002	CHEMBL612386
Yarrowia lipolytica	0.2580	CHEMBL612844
Mucor hiemalis	0.2478	CHEMBL612949
Aspergillus niger	0.2428	CHEMBL358
Penicillium marneffei	0.2043	CHEMBL612994
Aspergillus terreus	0.1878	CHEMBL612277
Cryptococcus bacillisporus	0.1593	CHEMBL615035
Saccharomyces cerevisiae	0.1496	CHEMBL361
Trichosporon asahii	0.1394	CHEMBL613164
Candida rugosa	0.1094	CHEMBL612869

Figure S102. 4′-Hydroxymethylflavanone 4-*O*-*β*-D-(4′′-*O*-methyl)-glucopyranoside (**4d**) antifungal activity prediction using the Way2Drug AntiFun-Pred modelling

Virus	Protein target	Confidence
Severe acute respiratory syndrome coronavirus 2	Replicase polyprotein 1ab	0.8171
Influenza A virus	Neuraminidase	0.0170
Varicella-zoster virus (strain Dumas) (HHV-3) (Human herpesvirus 3)	Thymidine kinase	0.0123

Figure S103. 4′-Hydroxymethylflavanone 4-*O*-*β*-D-(4′′-*O*-methyl)-glucopyranoside (**4d**) antiviral activity prediction using the Way2Drug AntiVir-Pred modelling
